# Photocatalytic direct borylation of carboxylic acids

**DOI:** 10.1038/s41467-022-34833-1

**Published:** 2022-11-19

**Authors:** Qiang Wei, Yuhsuan Lee, Weiqiu Liang, Xiaolei Chen, Bo-shuai Mu, Xi-Yang Cui, Wangsuo Wu, Shuming Bai, Zhibo Liu

**Affiliations:** 1 Beijing National Laboratory for Molecular Sciences, Radiochemistry and Radiation Chemistry Key Laboratory of Fundamental Science, NMPA Key Laboratory for Research and Evaluation of Radiopharmaceuticals, Key Laboratory of Bioorganic Chemistry and Molecular Engineering of Ministry of Education, College of Chemistry and Molecular Engineering, Peking University, Beijing, 100871 China; 2 Beijing National Laboratory for Molecular Sciences, State Key Laboratory for Structural Chemistry of Unstable and Stable Species, Institute of Chemistry, Chinese Academy of Sciences, Beijing, 100190 China; 3 Radiochemistry Laboratory, School of Nuclear Science and Technology, Lanzhou University, Lanzhou, 730000 China; 4 Peking-Tsinghua Center for Life Sciences, Peking University, Beijing, 100871 China

**Keywords:** Synthetic chemistry methodology, Photocatalysis, Organocatalysis

## Abstract

The preparation of high value-added boronic acids from cheap and plentiful carboxylic acids is desirable. To date, the decarboxylative borylation of carboxylic acids is generally realized through the extra step synthesized redox-active ester intermediate or in situ generated carboxylic acid covalent derivatives above 150 °C reaction temperature. Here, we report a direct decarboxylative borylation method of carboxylic acids enabled by visible-light catalysis and that does not require any extra stoichiometric additives or synthesis steps. This operationally simple process produces CO_2_ and proceeds under mild reaction conditions, in terms of high step economy and good functional group compatibility. A guanidine-based biomimetic active decarboxylative mechanism is proposed and rationalized by mechanistic studies. The methodology reported herein should see broad application extending beyond borylation.

## Abstract (web summary)

Boronic acids are useful reagents in organic synthesis and thus methods to prepare these compounds under mild conditions are desirable. Here, the authors synthesize boronic acids via photocatalytic decarboxylation of benzoic acids followed by borylation; guanidine-type additives are used to assist the process.

## Introduction

Chemistry of boronic acids has expanded far beyond the Suzuki reaction and is multiplying, and has become important in medicinal chemistry, analytical chemistry, chemical biology, and materials science. For example, the clinically approved antifungal drugs Tavaborole and eczema treatment drugs Eucrisa, a series of antibacterial or antitumor molecules under investigation with excellent biological activity, boronate-responsive probes or prodrugs, and even the borono-phenylalanine for Boron Neutron Capture Therapy^1–6^.

Given the importance of boronic acids, the development of methods that enables the borylation of all common carbon-heteroatom bonds, including C-O, C-N (ammonium salt and aryl diazonium), and C-X (Cl, Br, I and hypervalent iodine reagents), C-S (sulfonium salts) bonds is desirable^7–19^. Despite their synthetic versatility, the preparation of these compounds can be challenging, and these compounds can be unstable, with limited structure, or their synthesis may require forcing reaction conditions that somewhat limit their application. Among them, mild phenyl/aryl radical reactions are more suitable for the development of borylation^20–26^.

**Fig. 1:**
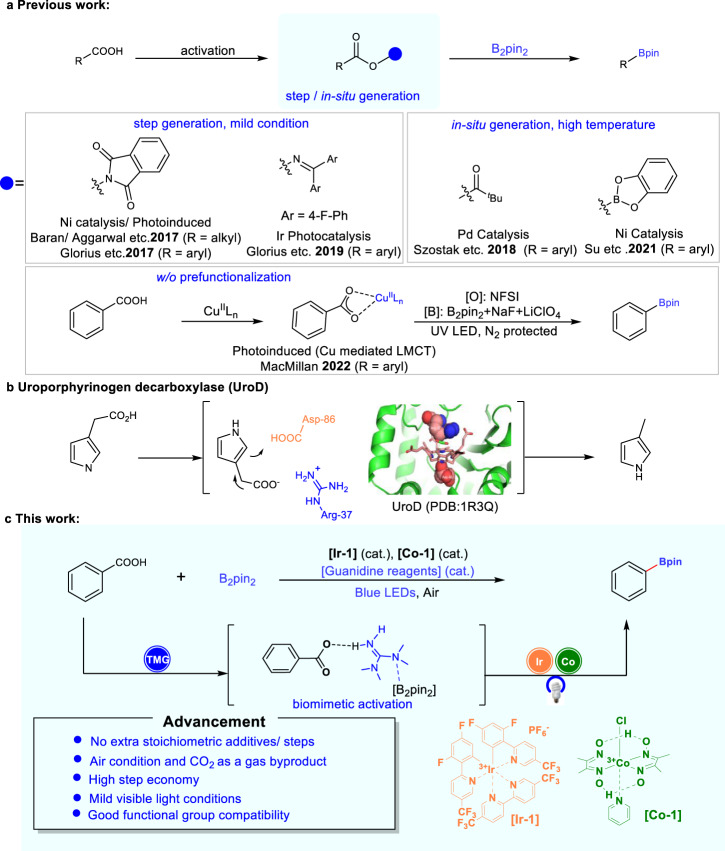
Introduction. **a** Previous reported decarboxylative borylation. **b** Mechanism of Uroporphyrinogen decarboxylase (UroD). **c** This work on the direct borylation. Cat., catalyst.

Stable and structurally diverse carboxylic acids exist in many natural products and are frequently accessible from commercial sources^27–29^. Carboxylic acids are well-established versatile building blocks for the construction of various chemical bonds, including C–H, C–O, C–N, C–S, and C–F bonds. However, it is curious that there were until recently no precedents for the construction of C-B bond from carboxylic acids^30^. Significant progress has been addressed indirectly through step-generated alkyl carboxylic acid *N*-Hydroxyphthalimide (NHPI) esters (redox-active esters) by the work of Baran, Aggarwal, etc.^31–33^. Glorius further completed the borylation of aryl carboxylic acid NHPI ester via a conceptually novel electron donor-acceptor complex^34,35^. Apart from the step synthesized redox-active ester strategy^23,36–43^, the group of Szostak realized the palladium-catalyzed decarbonylative borylation via in situ generated carboxylic acid anhydride intermediate in one-step synthesis^44^; Soon after, the group of Su developed the nickel-catalyzed decarbonylative borylation via in situ generated benzoic acid aryloxyboron intermediate under similar high-temperature condition^45^. Compared with metal-catalyzed borylation at high temperatures, the photocatalytic strategy with milder conditions has significant advantages in borylation reaction^34,35^ (Fig. 1a).

More recently, oxidative radical decarboxylation with a higher step economy has been studied because of the lower activation energy for the decarboxylation (8–9 kcal mol^−1^). However, the rate of direct decarboxylation of benzoic acids (k ≈ 10^6^ s^−1^) is not competitive with nondecarboxylative pathways, including the addition to arenes (k ≈ 10^8^ M^−1^s^−1^), hydrogen atom transfer (HAT) (k ≈ 10^7^ M^−1^ s^−1^), as well as back electron transfer (BET) between the generated aryl carboxyl radical and a reduced photocatalyst^46–49^. Moreover, even if the aryl radicals are formed, they may still be hard to be trapped by boronating reagents at rates that approach rates of its diffusion^50–53^, which further limits the direct decarboxylation borylation. Just recently, MacMillan published their latest work on the direct decarboxylative borylation without prefunctionalization of the native acid, which exploits UV photoinduced copper ligand-to-metal charge transfer (Cu-LMCT) to convert aryl acids into aryl radicals (Fig. 1a)^54^.

Inspired by the biomimetic approaches of enzymatic decarboxylation, scientists have been working on developing new decarboxylative functionalization strategies^55,56^. For example, based on the biosynthesis of polyketide, all kinds of decarboxylative reactions of malonic acid half thio/oxyester were developed by metal/organocatalysts^57^. Except for the biosynthesis of polyketide, uroporphyrinogen decarboxylase (UroD) may catalyze uroporphyrinogen decarboxylation for the biosynthesis of heme, chlorophyll, and the cytochromes in plants and animals^58^. It is worth noting that UroD can enhance the rate of substrate decarboxylation by a factor of 1.2 × 10^17^, which is one of the most significant rate enhancements and has been reported as an enzyme that acts without cofactors. The protonated basic residue (guanidino group of Arg-37) of UroD is thought to play a significant role in active carboxylic acid by furnishing a counterion, which assists the scissile carboxylate group of the substrate enter a relatively nonpolar environment and stabilizes the carbanion generated by the departure of CO_2_ (Fig. 1b). Inspired by this, we introduce a simplified guanidine-based molecule with both basic and nucleophilic properties to promote direct decarboxylative borylation. The guanidine can active the phenyl carboxyl acids, while promoting the generation and conversion of phenyl carboxyl radicals by balancing the capture rate and its diffusion rates. Herein we report a photocatalytic direct decarboxylative borylation method of aromatic carboxylic acids without extra stoichiometric additives or synthesis steps (Fig. 1c).

## Results and discussion

### Optimization of the reaction conditions

To test our hypothesis, we take 3-acetylbenzoic acid (**1n**) as a model compound to investigate the decarboxylation borylation. Due to the high oxidation potential (E_ox_ = 1.4 V) from benzoic acid to phenyl radical, the metal-based photocatalysts [Ir(dF(CF_3_ppy)_2_)(5,5′-CF_3_-bpy)]PF_6_ **[Ir-1]** (Ir^III*/II^ = 1.68 V versus SCE in MeCN)^59–61^ was chosen first in the presence of ethyl acetate solvent, boronating reagents B_2_pin_2_ and 440 nm blue light condition, the raw materials **1n** does not have any conversion. (Table 1, entry 1) To our delight, when the guanidine-based reagents tetramethylguanidine (TMG) was added, the borylated product **2n** was acquired in 15% yield, which is a preliminary confirmation of our biomimetic reaction design (entry 2). Besides, different transition-metal-catalysts were screened, and only when Co(dmgH)_2_pyCl (**Co-1**) was used in the state containing TMG, the yield of **2n** was surprisingly raised from 15 to 56% (entry 4 and Supplementary Table 4). Based on the condition in entry 4, we further screened other TMG analogs with imine protecting group or conformationally constrained cyclic molecules (entry 5–8, also see Supplementary Table 2), but no better results were acquired. Also, no product was obtained when other photocatalysts with higher or similar oxidation potential were tested, such as Mes-AcrClO_4_ (E_1/2 red_ = 2.06 V vs SCE), Ru(bpz)_3_PF_6_ (Ru^II*/I^ = 1.45 versus SCE in MeCN); then a series of screening on the Ir-based photocatalysts with different modification of the ligands was also conducted (entry 9–11, also see Supplementary Table 1), the change in yield is positively correlated with the oxidation potential of different photocatalysts. Interestingly, only the carboxylic acid ester solvents could promote the transformation, and the AcO^*t*^Bu solvent gained the best result with a 70% yield (entry 12, and Supplementary Table 7). Compared with the argon protection conditions at less than 10% yield, the influence of the air atmosphere on the reaction is huge, indicating that the oxygen in the air participates in the catalytic cycle (see Supplementary Table 5). After a detailed optimization of photosensitizer, guanidine-based reagents, cobalt (III) reagents, and boronating reagents were conducted, a 78% yield was acquired as the optimal condition (entry 13), which was provided in the Supplementary Table 10.

**Table 1:** Optimization of Direct Decarboxylative Borylation


Entry	Variation from standard conditions^a^	Result^b^
1	**[Ir-1]** (1 mol%)	n.d.
2	**[Ir-1]** (1 mol%), TMG (75 mol%)	15%
3	**[Ir-1]** (1 mol%), **[Co-1]** (10 mol%)	n.d.
4	**[Ir-1]** (1 %), TMG (75 %), **[Co-1]** (10 %)	56%
5	TBD instead of TMG in entry 4	32%
6	BTM instead of TMG in entry 4	27%
7	MTBD instead of TMG in entry 4	21%
8	HMPA instead of TMG in entry 4	n.d.
9	Mes-Acr-MeBF_4_ (20 mol%) instead of **[Ir-1]** in entry 4	n.d.
10	[Ru(bpz)_3_][PF_6_]_2_ (5 mol%) instead of **[Ir-1]** in entry 4	n.d.
11	[Ir(ppy)_2_(dtbbpy)]PF_6_ **[Ir-7]** (1 mol%) instead of **[Ir-1]** in entry 4	n.d.
12	AcO^*t*^Bu (2 mL) instead of Ethyl acetate in entry 4	70%
13^c^	**[Ir-1]** (3 mol%), **[Co-1]** (15 mol%), TMG (50 mol%)	78% (69%)^d^

^a^Standard conditions: **1n** (0.1 mmol), B_2_pin_2_ (2 equiv.), Ethyl acetate (1 mL), 440 nm LED, 24 h. **[Ir-1]**, [Ir(dF(CF_3_ppy)_2_)(5,5′-CF_3_-bpy)]PF_6_. **[Co-1]**, Co(dmgH)_2_pyCl. **[Ir-7]**, [Ir(ppy)_2_(dtbbpy)]PF_6_.

^b^Yield was determined by HPLC with 1,3,5-trimethoxybenzene as an internal standard. n.d., not detected.

^c^The optimized condition: **1n** (0.1 mmol), **B**_**2**_**pin**_**2**_ (1.5 equiv.), **[Ir-1]** (3 mol%), **[Co-1]** (15 mol%), TMG (50 mol%), AcOtBu (2 mL), 440 nm LED, 24 h. AcOtBu, *tert*-butyl acetyl ester.

^d^Isolated yield.

### Substrate scope

**Fig. 2:**
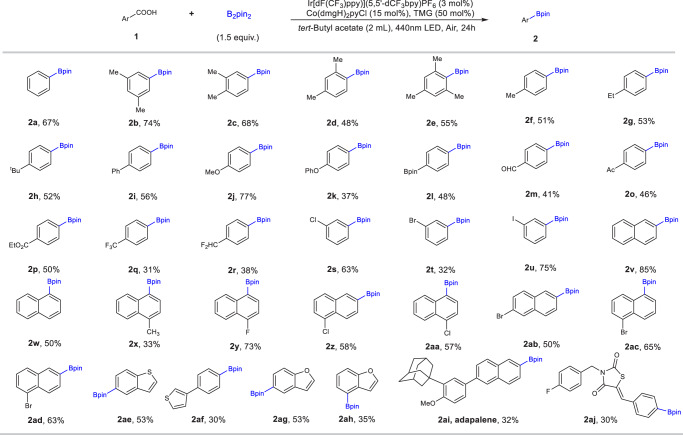
Substrate scope. Standard conditions: **1n** (0.1 mmol), B_2_pin_2_ (1.5 eq), **[Ir-1]** (3 mol%), **[Co-1]** (15 mol%), TMG (50 mol%), *tert*-Butyl acetate (2 mL), 440 nm LED, 24 h. Isolated yield of products. TMG, *N, N’*-Tetramethylguanidine.

We further evaluated the substrate scope of the photocatalytic direct decarboxylative borylation using a series of carboxylic acids substrates (Fig. 2). Benzoic acids with electron-donating groups (e.g. alkyl and alkoxy) were completely suitable substrates (**2a**-**2k**). Various substitution patterns (o-, m-, and p-) were tolerated (**2b**-**2e**). Similarly, benzoic acids with electron-withdrawing groups (ethyl carboxylate, acetyl, trifluoromethyl) were converted to the corresponding boronic esters in low to moderate yields (**2l**-**2r**). The high functional group compatibility was shown by successful decarboxylative borylation with functional groups, including acid-base sensitive difluoro methyl (**2r**), oxidation-sensitive aldehydes (**2** **m**), and thiophene ring (**2ae** and **2af**), enolizable ketones (**2o**), especially aryl halides (F, Cl, Br, I) (**2s**-**2ad**), which are commonly used and readily accessible precursors to boronic acids. The synthetic utility was further demonstrated by the borylation of the drugs and bioactive molecules (**2ai** and **2aj**). Borylation of activated carboxylic acids containing naphthalene and heteroaromatics, such as thiophene and furan, was also successful with a higher yield (**2v-2z**, **2aa-2ah**). Benzoic acids bearing strong coordinating or oxidizable functional groups, including amines and phenol failed to transform.

### Mechanistic considerations

**Fig. 3:**
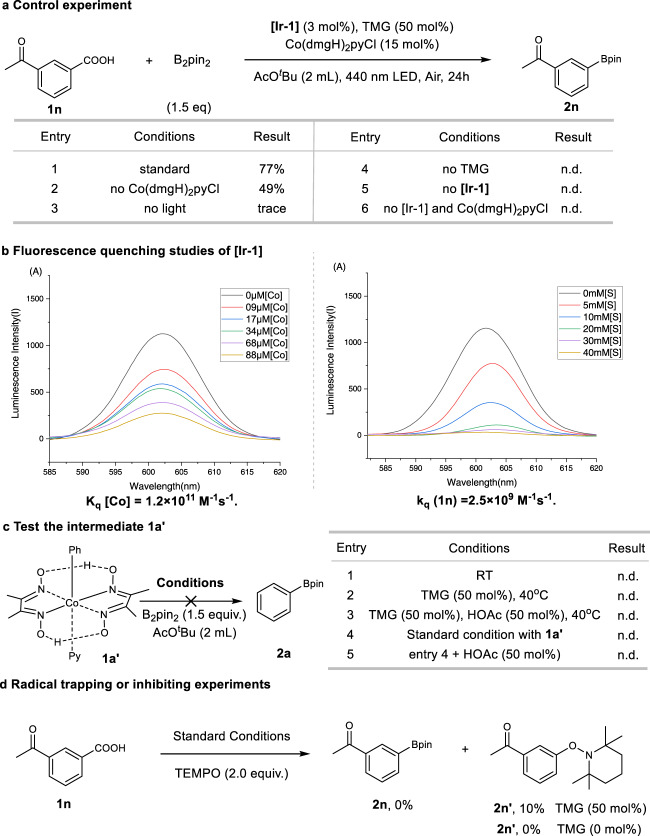
Control experiments for mechanistic investigation. For detailed conditions, see Supplementary Information including a control experiment to determine the key factors affecting the occurrence of reactions. **[Ir-1]**, [Ir(dF(CF_3_ppy)_2_)(5,5′-CF_3_-bpy)]PF_6._ AcO^*t*^Bu, *tert-*butyl acetyl ester. TMG, *N, N’*-Tetramethylguanidine. n.d., not detected. **a** Control experiments for mechanistic study. **b** Fluorescence quenching studies of photocatalysts **Ir-1** (the varied colors lines represent corresponding data at different quench concentrations). **[Co]**, Co(dmgH)_2_pyCl. **[S]**, **1n**. **c** To test if the **1a’** is the key intermediate in the reaction. Standard conditions: **1n** (0.1 mmol), B_2_pin_2_ (1.5 eq), **[Ir-1]** (3 mol%), **[Co-1]** (15 mol%), TMG (50 mol%), *tert*-Butyl acetate (2 mL), 440 nm LED, 24 h. RT, room temperature. **d** Radical trapping or inhibiting experiments. Yield was determined by HPLC with 1,3,5-trimethoxybenzene as an internal standard. TEMPO, 2,2,6,6-Tetramethyl-1-piperinedinyloxy.

We next turned our attention to the mechanism of this decarboxylation process. Based on the above results and further control experiments, the biomimetic active process of TMG and **[Ir**^**III**^**]** catalyst with specific high oxidation potential were all essential for the success of this new decarboxylative borylation protocol (entry 4–5, Fig. 3a). The presence of Co catalyst increases the reaction driving force, enhancing the reaction yield by 30% (entry 1–2, Fig. 3a). After the reaction, CO_2_ was detected by gas chromatography-mass spectrometry (see Supplementary Fig. 2). According to a Stern–Volmer analysis (Fig. 3b and Supplementary Figs. 3–5), oxidative quenching of the photoexcited **[Ir**^**III**^]* catalyst by **[Co**^**III**^**]** (k_q_(**Co-1**) = 1.2 × 10^11^ M^−1^s^−1^) is more likely than reductive quenching by carboxylate (k_q_ (**1n**) = 2.5 × 10^9^M^−1^s^−1^) in AcO^t^Bu, which is consistent with oxidation to **[Ir**^**IV**^**]**, rather than the oxidation of the carboxylate by the photoexcited state of the photocatalyst (see below).

Since cobalt phenyl complex **1a’** was not observed during the reaction, the cobalt phenyl complex **1a’** was prepared independently, and its reactivity has been investigated. The pinacol phenyl boronate product **2a** cannot be formed in the reaction whether under the same light conditions or heating conditions (Fig. 3c, Supplementary Table 12). The above results indicate that the Co catalyst **[Co-1]** may not participate in the borylation process, but only take part in the oxidative activation of the **[Ir**^**III**^**]** photosensitizer (Ir^III*/II^ = 1.68 V versus SCE in MeCN) to **[Ir**^**IV**^**]** (Ir^IV/III^ = 1.94 V versus SCE in MeCN)^60^ with higher oxidation potential.

Furthermore, the radical trapping reagents TEMPO completely suppresses the borylation of **1n** (Fig. 3d), which suggests the presence of free radicals in the reaction; Interestingly, only in the presence of TMG, the TEMPO-captured product **2n’** could be produced.

**Fig. 4:**
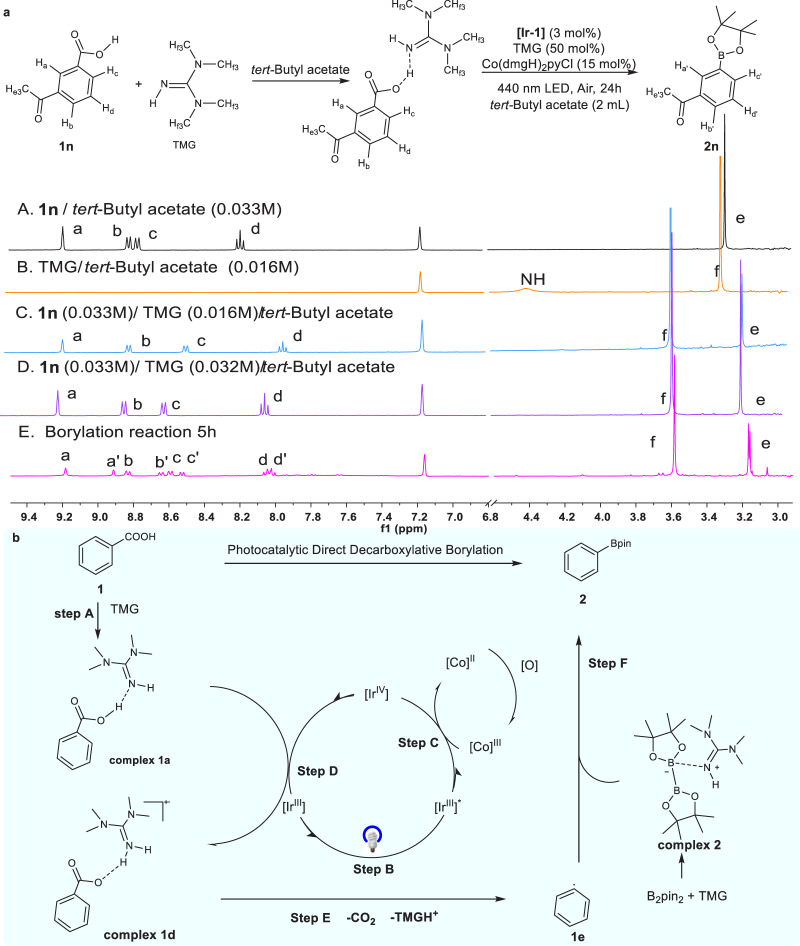
The proposed mechanism based on experimental exploration. **a** Condition of in situ ^1^HNMR analysis A: **1n** (0.33 M) in AcO^t^Bu (the date shown as the black line), B: TMG (0.016 M) in in AcO^*t*^Bu (the date shown as the orange line), C: **1n** (0.33 M), TMG (0.016 M) in AcO^*t*^Bu (the date shown as the blue line), D: **1n** (0.33 M), TMG (0.032 M) in AcO^*t*^Bu (the date shown as the purple line), E: Standard condition in 5 h (the date shown as the pink line). **[Ir-1]**, [Ir(dF(CF_3_ppy)_2_)(5,5′-CF_3_-bpy)]PF_6._ **b** Plausible mechanism.

The in situ ^1^H nuclear magnetic resonance (NMR) experiments were then carried out to investigate the mechanism. The reactions were conducted in screw-cap NMR tubes with AcO^*t*^Bu for convenient monitoring of the actual reaction progress. The mixing of substrate **1n** and TMG forms the corresponding **complex 1** based on the hydrogen bonding and makes the original *H* signal of **1n** move to the high field and the *H* signal of TMG move to the low field (Fig. 4a, c, d). Under standard conditions, the reaction proceeds with 50% **complex 1a** and 50% product **2n** after 5 h (Fig. 4a, e). This suggests that the **complex 1a** consisting of carboxylic acid substrate **1n** and TMG is the crucial intermediate for decarboxylation of the carboxylic acid and turnover of the photocatalytic cycle. The presence of TMG resulted in a significant reduction in the ^11^B NMR signal of B_2_pin_2_, which showed the possible activation of TMG on B_2_pin_2_ to form **complex 2** (Fig. 4b and Supplementary Fig. 7). Under the SET oxidation process of **[Ir**^**IV**^**]** (Step D, Fig. 4b), the stabilized phenyl carboxyl ion free radical **complex 1d** was generated and transferred to borylated product **2** quickly via the interaction between phenyl free radical **1e** and **complex 2** (Step E and F, Fig. 4b).

**Fig. 5:**
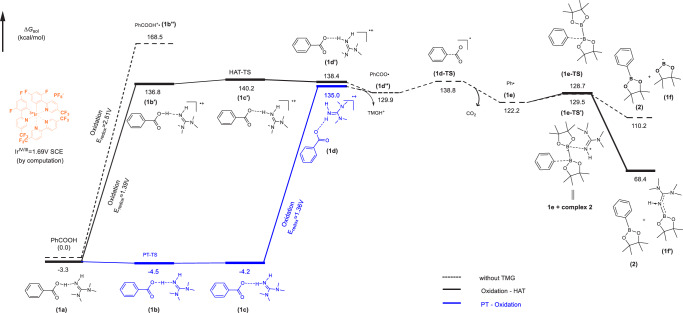
DFT-calculated reaction energy profile. Free energy profile for the activation of benzoic acid with (solid lines) and without (dashed lines) TMG computed with B3LYP-D3/6-311 + G(d,p) scrf = (smd, solvent = ethyl-ethanoate)^65,66^. HAT hydrogen atom transfer, PT proton transfer.

According to Garda’s work^62^, benzoic acid and TMG forms stable complexes by hydrogen bond interaction, with the changing of chemical properties. We computed the redox potential from benzoic acid to phenyl carboxyl radical using quantum chemical methods of density functional theory, and the result is +2.51 V in ethyl-ethanoate solvent. Meanwhile, the Ir^IV/III^ redox potential of metal-based photocatalysts **[Ir-1]** was computed as +1.69 V (which was measured as +1.94 V versus SCE in MeCN experimentally^60^), which is quite lower than the redox potential just mentioned. Although the redox potential of PhCOO^−^ is 0.54 V, it is hardly accessible in nonpolar organic solvent because of poor solubility, while for the whole sodium benzoate PhCOONa, the redox potential will increase to 1.82 V. When the TMG was added to PhCOOH (**1**) to form **complex 1a**, there are two allowed reaction paths: the one with oxidation followed by HAT (hydrogen atom transfer) (Oxidation-HAT), and the proton transfer followed by oxidation (PT-Oxidation), as shown in Fig. 5, the redox potential of benzoic acid drops significantly with the changed computation value to *E* = + 1.36 V in PT-Oxidation and +1.39 V in Oxidation-HAT, demonstrating the activation of carboxylic acids by TMG (see Supplementary Information for computational details).

The change of redox potential and the singly occupied molecular orbitals (SOMO) as the orbital where the redox process happens (see more details in Supplementary Table 18), tells the key roles of TMG in the oxidation process: First, by contributing the main component of the SOMO where the electron is transferred from as in **complex 1a** in Oxidation-HAT process, or being the proton acceptor as **complex 1c** in PT-Oxidation process, TMG has effectively lower the redox potential. Secondly, TMG and benzoic acids form hydrogen binding complex **1a**/**1c** to promote the redox reaction by maintaining good solubility in organic solvents.

After forming the free radical of benzoic acid-TMG complex **1d’/1d**, **1e** is then generated with the spontaneous dissociation of TMGH^+^ (ΔG = −5.1/−8.5 kcal/mol) and departure of CO_2_ gas (ΔG^‡^ = 8.9 kcal/mol). In addition to the activation in decarboxylation, the role of TMG in forming **complex 2** and then activating B_2_pin_2_ has also been computationally corroborated_._ Although it makes little change on the transition state energy barrier (ΔG^‡^ = 7.3 kcal/mol for **1e-TS’** vs 6.5 kcal/mol for **1e-TS**), the final state energy of borylation with TMG (68.4 kcal/mol for **2** + **1** **f’**) is much lower than the reaction without TMG (110.2 kcal/mol for **2** + **1** **f**). We thus conclude that TMG could stabilize the cleavage product of B_2_pin_2_ during borylation process, and increase the thermodynamic driving force.

In conclusion, we have described an approach that enables direct decarboxylative borylation from renewable carboxylic acids that does not require extra stoichiometric additives, showing a path that does not involve the step synthesis of redox-active ester or in situ synthesis of carboxylic acid covalent derivatives in harsh conditions. The scope and functional group tolerance of this method were further demonstrated on several representative carbocyclic substrates and medicinally relevant compounds. A guanidine-based biomimetic active decarboxylative strategy was introduced successfully to improve decarboxylation kinetics of aromatic carboxylic acids and the borylation process, as confirmed by systematic computational studies. This work expands the types of biomimetic decarboxylation catalysis and improves the step economy of decarboxylation borylation reactions, which should see broad application extending beyond borylation.

## Methods

### General methods

For ^1^H and ^13^CNMR spectra of compounds in this manuscript and details of the synthetic procedures, see Supplementary Information.

### General procedure for decarboxylative borylation

A 5 mL vial equipped with a magnetic stir bar was charged with 3- acetyl -carboxylic acid (16.5 mg, 0.1 mmol), [Ir(dF(CF_3_ppy)_2_)(5,5′-CF_3_-bpy)]PF_6_ (3.6 mg, 0.003 mmol), Co(dmgH)_2_pyCl (6 mg, 0.015 mmol), B_2_pin_2_ (39 mg, 0.15 mmol), AcO^*t*^Bu (2 mL) was then into the vial. The reaction mixture was stirred without irradiation for 10 min at ambient temperature, and then TMG (7 μL, 0.05 mmol) was gradually added to the vial under stirring. The reaction mixture was stirred without irradiation for another 10 min at ambient temperature and then irradiated for 24 h while maintaining the temperature at ~35 °C through cooling with a fan. The solvent was removed on a rotary evaporator under reduced pressure, and the residue was purified by preparative thin-layer chromatography.

### Computational study

All density functional theory (DFT) calculations were carried out with the Gaussian 16, Revision A.03. The B3LYP functional is mainly used in this research, and the 6–311 + g(d,p) basis set was used for organic molecules, For metal-containing molecules, light elements (C, H, N, O etc) were treated using the 6–31 G* basis set while metal atoms (Co and Ir) were treated using the LanL2DZ/SDD basis set. For all the calculations, Grimme-D3 correction was utilized for the Empirical Dispersion^63^, and the solvent effect of ethyl-ethanoate was included with Truhlar’s SMD model^64^. More details of the calculation were added to Supplementary Information.

## Supplementary information

Supplementary Information

## Data Availability

The authors declare that data relating to the characterization of materials and products, general methods, optimization studies, experimental procedures, mechanistic studies, HRMS data, and NMR spectra are available within the article and the Supplementary Information or from the corresponding author upon request.
