# Supplementary material for: Photocatalytic direct borylation of carboxylic acids
Source: Nat Commun. 2022 Nov 19;13:7112. doi: 10.1038/s41467-022-34833-1 (PMC9675845; doi:10.1038/s41467-022-34833-1)
Supplement: Supplementary file 1 — Supplementary Information [file 41467_2022_34833_MOESM1_ESM.pdf]

Supplementary Information

Photocatalytic Direct Borylation of Carboxylic Acids

Qiang Wei[1], Yuhsuan Lee[2], Weiqiu Liang[1], Xiaolei Chen[3], Bo-shuai Mu[1], Xi-Yang Cui[1], Wangsuo Wu[3], Shuming Bai[2], Zhibo Liu[1, 4*]

[1] Beijing National Laboratory for Molecular Sciences, Radiochemistry and Radiation Chemistry Key Laboratory of Fundamental Science, NMPA Key Laboratory for Research and Evaluation of Radiopharmaceuticals, Key Laboratory of Bioorganic Chemistry and Molecular Engineering of Ministry of Education, College of Chemistry and Molecular Engineering, Peking University, Beijing 100871, China. *Email: zbliu@pku.edu.cn

[2] Beijing National Laboratory for Molecular Sciences, State Key Laboratory for Structural Chemistry of Unstable and Stable Species, Institute of Chemistry, Chinese Academy of Sciences, Beijing 100190, China.

[3] Radiochemistry Laboratory, School of Nuclear Science and Technology, Lanzhou University, Lanzhou, 730000, China.

[4] Peking-Tsinghua Center for Life Sciences, Peking University, Beijing 100871, China.

Supplementary Methods

1. General Information

All available compounds were purchased from commercial suppliers and were used without further purification unless otherwise noted.

NMR spectra were recorded on Bruker Advance 400 (400 MHz for ^{1}H; 100 MHz for ^{13}C) spectrometer. The chemical shifts (*δ*) are given in parts per million relatives to $CDCl_3$ (7.26 ppm for ^{1}H) or TMS (0 ppm for ^{1}H), and $CDCl_3$ (77.0 ppm for ^{13}C). ^{1}H, ^{13}C multiplicities are reported as follows: singlet (s), doublet (d), doublet of doublets (dd), quartet (q), multiplet (m), and broad resonance (br).

Flash column chromatography and preparative thin-layer chromatography were performed on silica gel;

Ultra-performance liquid chromatography-mass spectrometry (UPLC-MS) was performed on an ACQUITY UPLC H-Class PLUS instrument equipped with a Waters PDA eλ Detector and a Waters Acquity QDA mass spectrometer. High-resolution mass spectroscopy was performed on a Bruker Fourier Transform Ion Cyclotron Resonance Mass Spectrometer. Fluorescence spectra were measured on an F-7000 spectrophotometer. (Hitachi, Japan)

Unless otherwise noted, all reactions were carried out under an air atmosphere in screw-capped vials. A LED (30 W, λ max = 440 nm) may be shared by 14 screw-capped vials for irradiation obtained from Xuzhou Aijia Electronic Technology Co., LTD. In each case, each LED was set up, and the light source was placed ~2 cm from the reaction vessels (Fig. 1). A box was placed over the lights to shield the light. The reaction temperature was measured using a contact thermometer to be 35 °C.

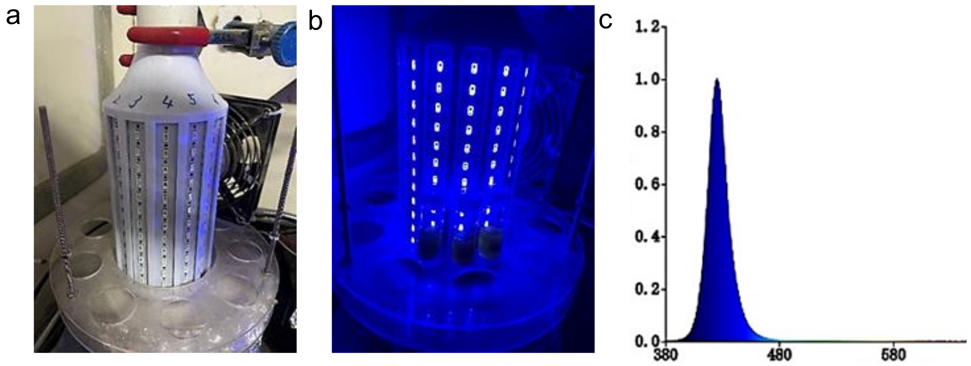

Supplementary Fig. 1 Photographs of the used light source in the reactions under irradiation of blue LED (λ = 440 nm). **a** reaction equipment with light source, air fans and magnetic stirrers. **b** Light reaction in progress. **c** The wavelength range of the light source, which is provided by Xuzhou Aijia Electronic Technology Co., LTD.

2. Procedure for Optimization Studies

A 5 mL vial equipped with a magnetic stir bar was charged with 3-acetyl-carboxylic acid (16.5 mg, 0.1 mmol), photocatalyst, [Co] catalyst, borylated reagent, or other additives; the anhydrous solvent was then into the vial. The reaction mixture was stirred without irradiation for 10 min at ambient temperature, and then a guanidine-based reagent was gradually added to the vial under stirring. The reaction mixture was stirred without irradiation for another 10 min at ambient temperature and then irradiated for 24 h while maintaining the temperature at approximately 35 °C through cooling with a fan. 1,3,5-trimethoxybenzene (HPLC internal standard quantification based on the standard internal 1,3,5-trimethoxybenzene, the peak area and relative correction factor of reaction product) was added then the mixture was analyzed by HPLC.

Supplementary Table 1. Evaluation of Photosensitizers.

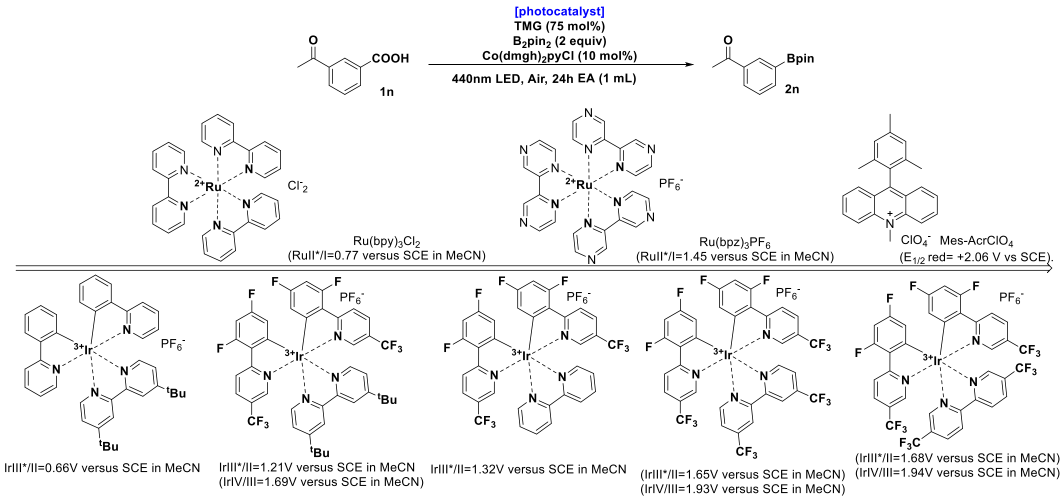

Conditions	Result[a]
1. $[Ir(dF(CF_3ppy)_2)(5,5'\text{-}CF_3\text{-}bpy)]PF_6$ **[Ir-1]** (1 mol%)	56%
2. $[Ir(dF(CF_3ppy)_2)(dtbbpy)]PF_6$ **[Ir-2]** (1 mol%)	8%
3. $[Ir(dF(CF_3ppy)_2)(bpy)]PF_6$ **[Ir-3]** (1 mol%)	5%
4. $[Ir(dF(CF_3ppy)_2)(4,4'\text{-}CF_3\text{-}bpy)]PF_6$ **[Ir-4]** (1 mol %)	23%
5. $[Ir(dCF_3(Fppy)_2)(dtbbpy)]PF_6$ **[Ir-5]** (1 mol %)	trace
6. $[Ir(dF(CF_3ppy)_2)(1,10\text{-phenanthroline})PF_6$ **[Ir-6]** (1 mol%)	trace
7. $[Ir(ppy)_2(dtbbpy)]PF_6$ **[Ir-7]** (1 mol%)	NR
8. Mes-Acr-$MeBF_4$ (20 mol%)	NR
9. [Ru(Tris(4,7-diphenyl-1,10-phenanthroline)]$(PF_6)_2$ (5 mol%)	NR
10. $[Ru(bpz)_3][PF_6]_2$ (5 mol%)	NR
11. [Ru(Tris(2,2'-bipyridine)]$(PF_6)_2$ (5 mol%)	NR

[a]Yield was determined by HPLC with 1,3,5-trimethoxybenzene as an internal standard.

Supplementary Table 2. Evaluation of Guanidine-based Cocatalysts

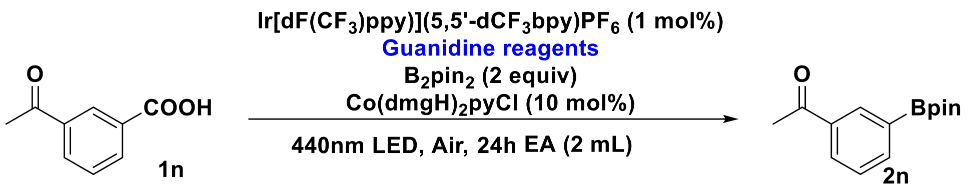

Conditions	Result[a]
1. No Guanidine	NR
2. TMG (0.75 equiv.)	56%
3. BTMG (0.75 equiv.)	32%
4. TBD (0.75 equiv.)	27%
5. MTBD (0.75 equiv.)	21%
6. HMPA (0.75 equiv.)	NR
7. TMG (0.25 equiv.)	15%
8. TMG (0.5 equiv.)	46%
9. TMG (0.75 equiv.)	56%
10. TMG (1 equiv.)	22%
11. TMG (2 equiv.)	2%

[a] Yield was determined by HPLC with 1,3,5-trimethoxybenzene as an internal standard.

Supplementary Table 3. Evaluation of Co Catalyst

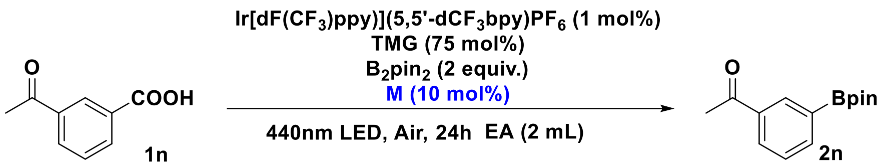

Conditions	Result[a]
1. No [Co]	15%
2. $Co(dmgH)_2Cl_2$	34%
3. $Co(dmgH)_2$pyCl	56%
4. $Co(dmgH)_2$(4-MeO)PyCl	51%
5. $Co(dmgH)_2$(4-diMeN)PyCl	51%
6. $Co(dmgH)_2$(4-tBu)PyCl	48%
7. $Co(dmgH)_2$(3,5-diMe)PyCl	44%
8. $Co(dmgH)_2$(4-CN)PyCl	32%
9. $Co(dmgH)_2$(4-CO_2Et)PyCl	31%
10. $Co(dmgH)_2$(4-Br)pyCl	22%
11. $Co(dmgH)_2$(3-Br)pyCl	26%
12. $Co(dmgH)_2$(4-I)pyCl	25%

[a]Yield was determined by HPLC with 1,3,5-trimethoxybenzene as an internal standard.

Supplementary Table 4. Evaluation of Other Metal Catalyst under Ar or Air conditions

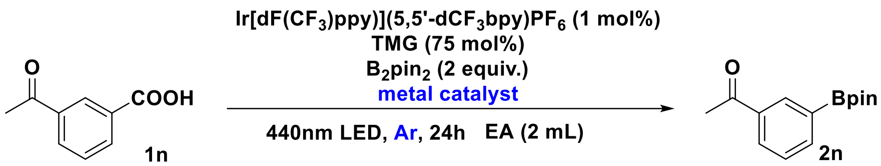

Conditions	Result[a] (Air condition)
1. No metal catalyst	Trace
2. Ag_2CO_3 (20%)	13% (no significant improvement)
3. AgI (20%)	11% (no significant improvement)
4. AgI (1 eq)	15% (no significant improvement)
5.$Cu(MeCN)_4BF_4$ (20%)	7% (no significant improvement)
6. $FeCl_2$ (20%)	3% (no significant improvement)
7. $NiCl_2$ (20%)	10% (no significant improvement)
8. $MnBr_2$ (20%)	2% (no significant improvement)
9. $Pd(OAc)_2$ (20%)	3% (no significant improvement)
10. $NiBr_2$(DME) (10%)	8% (no significant improvement)
11. $CoCl_2$ (10%)	8% (no significant improvement)
12. $Co(dmgH)_2$pyCl (10%)	9% (56%)
13. $Co(dmgH)_2$pyCl (100%)	28%

[a]Yield was determined by HPLC with 1,3,5-trimethoxybenzene as an internal standard.

Supplementary Table 5. Evaluation of Different Oxidants.

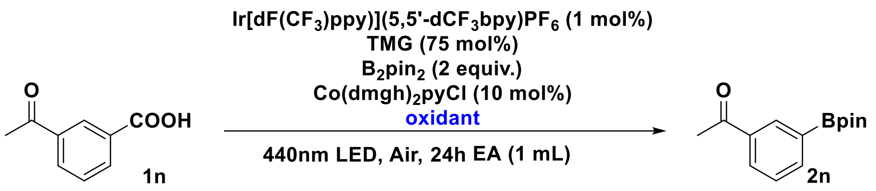

Conditions	Result[a]
1. conditions under Ar	9%
2. conditions under Air	56%
3. conditions under O_2	14%
4. conditions under Air + MnO_2 (1 equiv.)	19%
5. conditions under Air + $Na_2S_2O_8$ (1 equiv.)	47%
6. conditions under Air + p-BQ (1 equiv.)	NR
7. conditions under Air + H_2O (1 equiv.)	28%

[a] Yield was determined by HPLC with 1,3,5-trimethoxybenzene as an internal standard.

Supplementary Table 6. Evaluation of Borylated Reagent

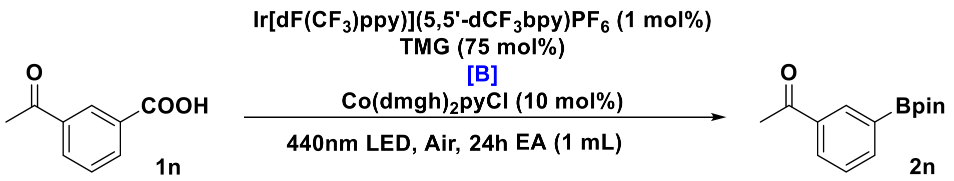

Conditions	Result[a]
1. B_2pin_2	56%
2. Bis(neopentyl glycolato)diboron	NR
3. Bis(catecholato)diborane	NR
4. Hypodiboric acid	NR
5. HBpin	NR
6. Trimethylamine borane	NR

[a] Yield was determined by HPLC with 1,3,5-trimethoxybenzene as an internal standard.

Supplementary Table 7. Evaluation of Solvents

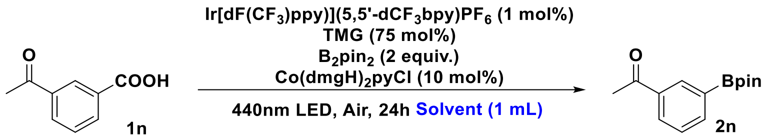

Conditions	Result[a]
1.MeCN	Trace
2. DMA	NR
3. DMSO	NR
5. MeOH	NR
6. Dioxane	Trace
7.Acetone	7%
8. THF	Trace
9. Toluene	Trace
10. Benzotrifluoride	<10%
11. HMPA	NR
12. CCl_4	NR
13. $MeNO_2$	NR
14. Trifluoroethanol	NR
15. EA	56%
16. *tert*-Butyl acetate	58%
17. Methyl acetate	13%
18. Cyclohexyl acetate	43%
19. Isopropyl acetate	52%
20. Propyl acetate	43%
21. 2,2,2-Trifluoroethyl acetate	18%
22. Ethyl propionate	30%
23. Methyl formate	Trace
24. Ethyl trifluoroacetate	NR
25. Ethyl bromoacetate	NR
26. Delta-Valerolactone	NR

27. *tert*-Butyl acetate (2 mL)	70%
28. *tert*-Butyl acetate (3 mL)	66%

Supplementary Table 8. Evaluation of TMG Loading

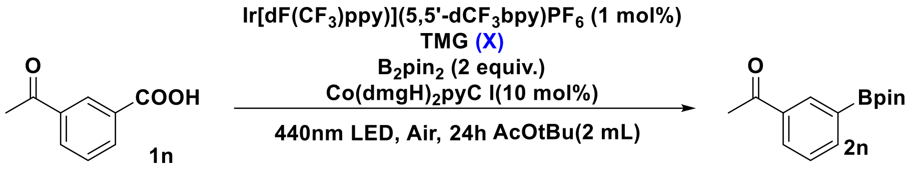

Conditions	Result[a]
1. TMG (75 mol%)	70%
2. TMG (100 mol%)	19%
3. TMG (50 mol%)	75%
4. TMG (25 mol%)	42%

[a]yield was determined by HPLC with 1,3,5-trimethoxybenzene as an internal standard.

Supplementary Table 9. Evaluation of Co Catalyst Loading

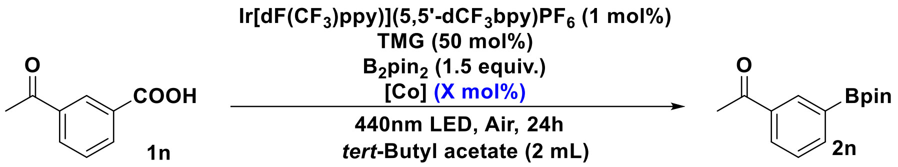

Conditions	Result[a]
1. Co(dmgH)$_2$pyCl (10 mol%)	70%
2. Co(dmgH)$_2$pyCl (5 mol%)	36%
3. Co(dmgH)$_2$pyCl (15 mol%)	72%

[a]Yield was determined by HPLC with 1,3,5-trimethoxybenzene as an internal standard.

Supplementary Table 10. Evaluation of Catalyst Loading

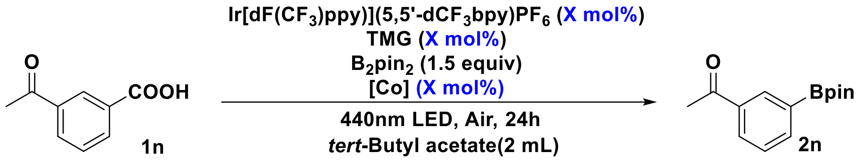

Conditions	Result[a]
1. [Ir] (3 mol%)	75%
2. [Ir] (3 mol%), TMG (50 mol%)	72%
3. [Ir] (3 mol%), [Co] (15 mol%), TMG (50 mol%)	78%

[a]Yield was determined by HPLC with 1,3,5-trimethoxybenzene as an internal standard.

3. Procedure for Decarboxylative Borylation.

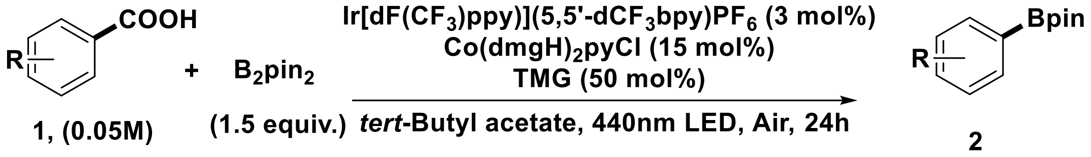

A 5 mL vial equipped with a magnetic stir bar was charged with 3- acetyl - carboxylic acid (16.5 mg, 0.1 mmol), $[Ir(dF(CF_3ppy)_2)(5,5'\text{-}CF_3\text{-}bpy)]PF_6$ (3.6 mg, 0.003 mmol), $Co(dmgH)_2pyCl$ (6 mg, 0.015mmol), B_2pin_2 (39mg, 0.15 mmol), AcO^{t}Bu (2 mL) was then into the vial. The reaction mixture was stirred without irradiation for 10 min at ambient temperature, and then TMG (7 µL, 0.05 mmol) was gradually added to the vial under stirring. The reaction mixture was stirred without irradiation for another 10 min at ambient temperature and then irradiated for 24 h while maintaining the temperature at approximately 35 °C through cooling with a fan. The solvent was removed on a rotary evaporator under reduced pressure, and the residue was purified by preparative thin-layer chromatography.

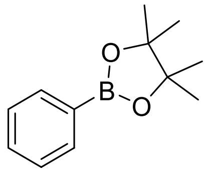

4,4,5,5-tetramethyl-2-phenyl-1,3,2-dioxaborolane (2a), The title compound **2a** was prepared according to the procedure and was purified by preparative thin-layer chromatography (PE/DCM: 1/1, Rf: 0.6). it was obtained as white solid (13 mg, 67%).^{1}**H NMR** (400 MHz, Chloroform-*d*) δ 7.81 (d, *J* = 6.9 Hz, 1H), 7.46 (t, *J* = 7.4 Hz, 1H), 7.37 (t, *J* = 7.4 Hz, 1H), 1.35 (s, 4H). ^{13}**C NMR** (101 MHz, Chloroform-*d*) δ 134.74, 131.25, 127.71, 83.77, 24.89. The carbon directly attached to the boron atom was not detected due to quadrupolar broadening. The NMR data were in consistent with the reported data.[1]

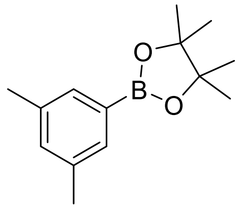

2-(3,5-dimethylphenyl)-4,4,5,5-tetramethyl-1,3,2-dioxaborolane (2b), The title compound **2b** was prepared according to the procedure and was purified by preparative thin-layer chromatography (PE/DCM: 1/1, Rf: 0.6). it was obtained as white solid (15 mg, 74%). ^{1}**H NMR** (400 MHz, Chloroform-*d*) δ 7.44 (s, 2H), 7.10 (s, 1H), 2.32 (s, 3H), 2.32 (s, 3H), 1.34 (s, 12H). ^{13}**C NMR** (101 MHz, Chloroform-*d*) δ 137.17, 132.99, 132.39, 83.69, 24.87, 21.15. The carbon directly attached to the boron atom was not detected due to quadrupolar broadening. The NMR data were in consistent with the reported data[2].

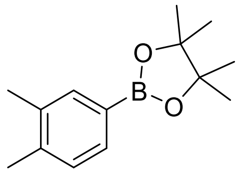

2-(3,4-dimethylphenyl)-4,4,5,5-tetramethyl-1,3,2-dioxaborolane (2c), The title compound **2c** was prepared according to the procedure and was purified by preparative thin-layer chromatography (PE/DCM: 1/1, Rf: 0.6). it was obtained as white solid (15 mg, 68%). ^{1}**H NMR** (400 MHz, Chloroform-*d*) δ 7.51

(s, 1H), 7.48 (d, J = 7.4 Hz, 1H), 7.08 (d, J = 7.4 Hz, 1H), 2.21 (s, 3H), 2.20 (s, 3H), 1.27 (s, 12H). ^{13}**C NMR** (101 MHz, Chloroform-*d*) δ 140.15, 135.93, 135.89, 132.40, 129.17, 83.59, 24.85, 20.03, 19.48. The carbon directly attached to the boron atom was not detected due to quadrupolar broadening. The NMR data were in consistent with the reported data[4].

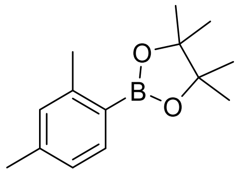

2-(2,4-dimethylphenyl)-4,4,5,5-tetramethyl-1,3,2-dioxaborolane (2d), The title compound **2d** was prepared according to the procedure and was purified by preparative thin-layer chromatography (PE/DCM: 1/1, Rf: 0.8). it was obtained as white solid (11 mg, 48%). ^{1}**H NMR** (400 MHz, Chloroform-*d*) δ 7.59 (d, J = 8.1 Hz, 1H), 6.94 – 6.88 (m, 2H), 2.43 (s, 3H), 2.24 (s, 3H), 1.26 (s, 12H). ^{13}**C NMR** (101 MHz, Chloroform-*d*) δ 144.94, 140.86, 136.09, 130.72, 125.53, 83.23, 24.89, 22.12, 21.50. The carbon directly attached to the boron atom was not detected due to quadrupolar broadening. The NMR data were in consistent with the reported data[2].

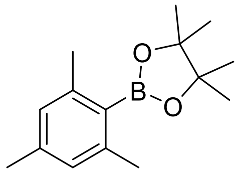

2-mesityl-4,4,5,5-tetramethyl-1,3,2-dioxaborolane (2e), The title compound **2e** was prepared according to the procedure and was purified by preparative thin-layer chromatography (PE/DCM: 1/1, Rf: 0.7). it was obtained as white solid (13 mg, 55%). ^{1}**H NMR** (400 MHz, Chloroform-*d*) δ 6.77 (s, 2H), 2.36 (s, 6H), 2.24 (s, 3H), 1.37 (s, 12H). ^{13}**C NMR** (101 MHz, Chloroform-*d*) δ 142.12, 138.92, 127.44, 83.46, 24.96, 22.20, 21.25. The carbon directly attached to the boron atom was not detected due to quadrupolar broadening. The NMR data were in consistent with the reported data[3].

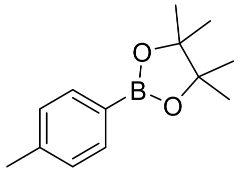

4,4,5,5-tetramethyl-2-(p-tolyl)-1,3,2-dioxaborolane (2f), The title compound **2f** was prepared according to the procedure and was purified by preparative thin-layer chromatography (PE/DCM: 1/1, Rf: 0.6). it was obtained as white solid (11 mg, 51%). **^{1}H NMR** (400 MHz, Chloroform-*d*) *δ* 7.70 (d, *J* = 7.6 Hz, 2H), 7.19 (d, *J* = 7.6 Hz, 2H), 2.36 (s, 3H), 1.34 (s, 12H). **^{13}C NMR** (101 MHz, Chloroform-*d*) δ 141.39, 134.81, 128.53, 83.62, 24.88, 21.74. The carbon directly attached to the boron atom was not detected due to quadrupolar broadening. The NMR data were in consistent with the reported data[1].

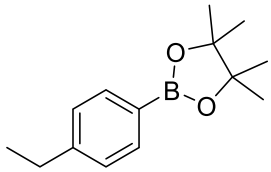

2-(4-ethylphenyl)-4,4,5,5-tetramethyl-1,3,2-dioxaborolane (2g), The title compound **2g** was prepared according to the procedure and was purified by preparative thin-layer chromatography (PE/DCM: 1/1, Rf: 0.5). it was obtained as white solid (12 mg, 53%). **^{1}H NMR** (400 MHz, Chloroform-*d*) *δ* 7.74 (d, *J* = 8.0 Hz, 2H), 7.21 (d, *J* = 7.9 Hz, 2H), 2.66 (q, *J* = 7.6 Hz, 2H), 1.34 (s, 12H), 1.23 (t, *J* = 7.7 Hz, 3H). **^{13}C NMR** (101 MHz, Chloroform-*d*) *δ* 147.74, 134.90, 127.36, 83.62, 29.12, 24.86, 15.49. The carbon directly attached to the boron atom was not detected due to quadrupolar broadening. The NMR data were in consistent with the reported data.[5]

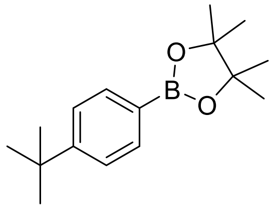

2-(4-(*tert*-butyl)phenyl)-4,4,5,5-tetramethyl-1,3,2-dioxaborolane (2h), The title compound **2h** was prepared according to the procedure and was purified by preparative thin-layer chromatography (PE/DCM: 1/1, Rf: 0.8). it was

obtained as white solid (13 mg, 52%). **^{1}H NMR** (400 MHz, Chloroform-*d*) δ 7.76 (d, *J* = 8.3 Hz, 2H), 7.41 (d, *J* = 8.3 Hz, 2H), 1.33 (s, 12H), 1.32 (s, 9H). **^{13}C NMR** (101 MHz, Chloroform-*d*) δ 154.50, 134.69, 124.71, 83.62, 34.91, 31.21, 24.85. The carbon directly attached to the boron atom was not detected due to quadrupolar broadening. The NMR data were in consistent with the reported data.[1]

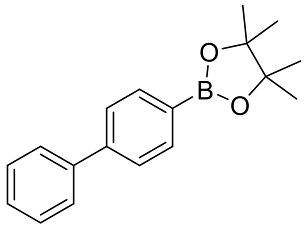

2-([1,1'-biphenyl]-4-yl)-4,4,5,5-tetramethyl-1,3,2-dioxaborolane (2i), The title compound **2i** was prepared according to the procudure and was purified by preparative thin-layer chromatography (PE/DCM: 1/1, Rf: 0.6). it was obtained as light yellow solid (15 mg, 56%). **^{1}H NMR** (400 MHz, Chloroform-*d*) δ 7.89 (d, *J* = 7.9 Hz, 2H), 7.64 – 7.59 (m, 4H), 7.44 (t, *J* = 7.6 Hz, 2H), 7.35 (t, *J* = 7.3 Hz, 1H), 1.36 (s, 12H). **^{13}C NMR** (101 MHz, Chloroform-*d*) δ 143.90, 141.04, 135.27, 128.78, 127.57, 127.25, 126.48, 83.83, 24.91. The carbon directly attached to the boron atom was not detected due to quadrupolar broadening. The NMR data were in consistent with the reported data[1].

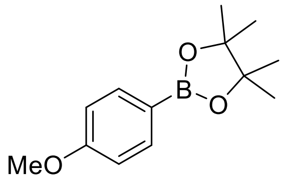

2-(4-methoxyphenyl)-4,4,5,5-tetramethyl-1,3,2-dioxaborolane (2j), The title compound **2j** was prepared according to the procedure and was purified by preparative thin-layer chromatography (PE/DCM: 1/1, Rf: 0.4). it was obtained as white solid (17 mg, 77%). **^{1}H NMR** (400 MHz, Chloroform-*d*) δ 7.75 (d, *J* = 8.6 Hz, 2H), 6.90 (d, *J* = 8.6 Hz, 2H), 3.83 (s, 3H), 1.33 (s, 12H). **^{13}C NMR** (101 MHz, Chloroform-*d*) δ 162.14, 136.51, 113.31, 83.55, 55.10, 24.87. The carbon

directly attached to the boron atom was not detected due to quadrupolar broadening. The NMR data were in consistent with the reported data[1].

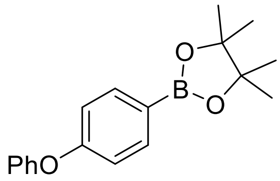

4,4,5,5-tetramethyl-2-(4-phenoxyphenyl)-1,3,2-dioxaborolane (2k), The title compound **2k** was prepared according to the procedure and was purified by preparative thin-layer chromatography (PE/DCM: 1/1, Rf: 0.7). it was obtained as white solid (10 mg, 37%). **^{1}H NMR** (400 MHz, Chloroform-*d*) *δ* 7.78 (d, *J* = 8.5 Hz, 2H), 7.38 – 7.32 (m, 2H), 7.13 (t, *J* = 7.4 Hz, 1H), 7.06 – 7.01 (m, 2H), 6.98 (d, *J* = 8.6 Hz, 2H), 1.34 (s, 12H). **^{13}C NMR** (101 MHz, Chloroform-*d*) *δ* 160.18, 156.54, 136.63, 129.80, 123.66, 119.47, 117.67, 83.75, 24.88. The carbon directly attached to the boron atom was not detected due to quadrupolar broadening. The NMR data were in consistent with the reported data[6].

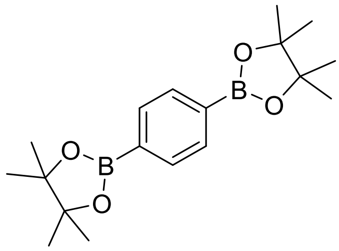

1,4-bis(4,4,5,5-tetramethyl-1,3,2-dioxaborolan-2-yl)benzene (2l), The title compound **2l** was prepared according to the procedure and was purified by preparative thin-layer chromatography (toluene/EA: 1/20, Rf: 0.6). it was obtained as white solid (15 mg, 48%). **^{1}H NMR** (400 MHz, Chloroform-*d*) *δ* 7.73 (s, 4H), 1.28 (s, 12H). **^{13}C NMR** (101 MHz, Chloroform-*d*) *δ* 133.88, 83.84, 24.88. The carbon directly attached to the boron atom was not detected due to quadrupolar broadening. The NMR data were in consistent with the reported data.[2]

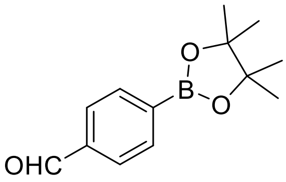

4-(4,4,5,5-tetramethyl-1,3,2-dioxaborolan-2-yl)benzaldehyde (2m), The title compound **2m** was prepared according to the procedure and was purified by preparative thin-layer chromatography (toluene/DCM: 1/1, Rf: 0.5). it was obtained as white solid (9 mg, 41%). **^{1}H NMR** (400 MHz, Chloroform-*d*) δ 9.98 (s, 1H), 7.89 (d, J = 8.2 Hz, 2H), 7.79 (d, J = 8.2 Hz, 2H), 1.29 (s, 12H). **^{13}C NMR** (101 MHz, Chloroform-*d*) δ 192.67, 138.11, 135.22, 128.71, 84.34, 24.90. The carbon directly attached to the boron atom was not detected due to quadrupolar broadening. The NMR data were in consistent with the reported data[2].

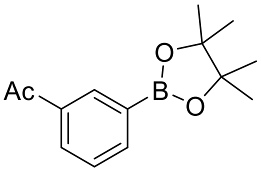

1-(3-(4,4,5,5-tetramethyl-1,3,2-dioxaborolan-2-yl)phenyl)ethan-1-one (2n), The title compound **2n** was prepared according to the procudure and was purified by preparative thin-layer chromatography ($CHCl_3$/DCM: 1/1, Rf: 0.5). it was obtained as white solid (16 mg, 69%). **^{1}H NMR** (400 MHz, Chloroform-*d*) δ 8.29 (s, 1H), 7.99 (d, J = 7.8 Hz, 1H), 7.92 (d, J = 7.3 Hz, 1H), 7.40 (t, J = 7.6 Hz, 1H), 2.57 (s, 3H), 1.29 (s, 12H). **^{13}C NMR** (101 MHz, Chloroform-*d*) δ 198.42, 139.41, 136.52, 134.81, 130.77, 128.04, 84.16, 26.77, 24.90. The carbon directly attached to the boron atom was not detected due to quadrupolar broadening. The NMR data were in consistent with the reported data[3].

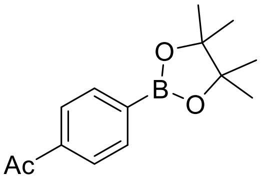

1-(4-(4,4,5,5-tetramethyl-1,3,2-dioxaborolan-2-yl)phenyl)ethan-1-one (2o),

The title compound **2o** was prepared according to the procedure and was purified by preparative thin-layer chromatography ($CHCl_3$/DCM: 1/1, Rf: 0.5). it was obtained as white solid (11 mg, 46%). **^{1}H NMR** (400 MHz, Chloroform-*d*) δ 7.84 (q, *J* = 8.3 Hz, 1H), 2.55 (s, 3H), 1.29 (s, 12H). **^{13}C NMR** (101 MHz, Chloroform-*d*) δ 198.46, 139.00, 134.92, 127.28, 84.22, 26.77, 24.88. The carbon directly attached to the boron atom was not detected due to quadrupolar broadening. The NMR data were in consistent with the reported data[7].

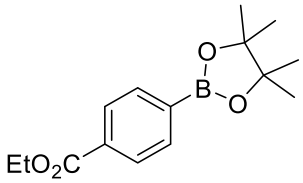

ethyl 4-(4,4,5,5-tetramethyl-1,3,2-dioxaborolan-2-yl)benzoate (2p), The title compound **2p** was prepared according to the procudure and was purified by preparative thin-layer chromatography ($CHCl_3$/DCM: 1/1, Rf: 0.5). it was obtained as white solid (14 mg, 50%). **^{1}H NMR** (400 MHz, Chloroform-*d*) δ 8.02 (d, *J* = 7.8 Hz, 2H), 7.87 (d, *J* = 7.8 Hz, 1H), 4.38 (q, *J* = 7.1 Hz, 2H), 1.40 (t, *J* = 7.1 Hz, 3H), 1.36 (s, 12H). **^{13}C NMR** (101 MHz, Chloroform-*d*) δ 166.67, 134.62, 132.67, 128.55, 84.16, 61.04, 24.89, 14.33. The carbon directly attached to the boron atom was not detected due to quadrupolar broadening. The NMR data were in consistent with the reported data[2].

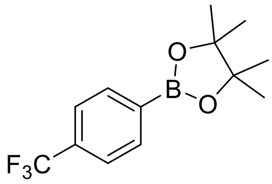

4,4,5,5-tetramethyl-2-(4-(trifluoromethyl)phenyl)-1,3,2-dioxaborolane (2q), The title compound **2q** was prepared according to the procudure and was purified by preparative thin-layer chromatography (toluene/DCM: 1/1, Rf: 0.5). it was obtained as white solid (8 mg, 31%). **^{1}H NMR** (400 MHz, Chloroform-*d*) δ 7.91 (d, *J* = 7.8 Hz, 2H), 7.61 (d, *J* = 7.8 Hz, 1H), 1.36 (s, 12H). **^{13}C NMR** (101 MHz, Chloroform-*d*) δ 135.00, 132.82(q, *J* = 32.1 Hz), 124.13 (d, *J* = 272.1 Hz),

124.31 (q, J = 3.6 Hz), 84.27, 77.33, 24.87. The carbon directly attached to the boron atom was not detected due to quadrupolar broadening. The NMR data were in consistent with the reported data[1].

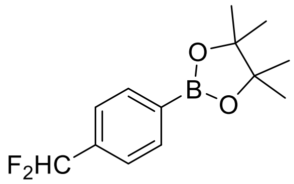

2-(4-(difluoromethyl)phenyl)-4,4,5,5-tetramethyl-1,3,2-dioxaborolane (2r), The title compound **2r** was prepared according to the procudure and was purified by preparative thin-layer chromatography (PE/DCM: 1/1, Rf: 0.5). it was obtained as white solid (9 mg, 38%). **^{1}H NMR** (400 MHz, Chloroform-*d*) δ 7.82 (d, J = 7.6 Hz, 2H), 7.43 (d, J = 7.7 Hz, 2H), 6.58 (t, J = 56.4 Hz, 1H), 1.28 (s, 12H). **^{13}C NMR** (101 MHz, Chloroform-*d*) δ 136.79 (t, J = 22.1 Hz), 135.02, 124.72 (t, J = 6.0 Hz), 114.66 (t, J = 238.8 Hz), 84.11, 24.88. The carbon directly attached to the boron atom was not detected due to quadrupolar broadening. The NMR data were in consistent with the reported data[8].

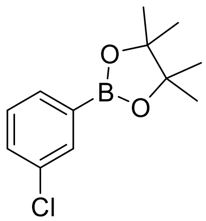

2-(3-chlorophenyl)-4,4,5,5-tetramethyl-1,3,2-dioxaborolane (2s), The title compound **2s** was prepared according to the procedure and was purified by preparative thin-layer chromatography (PE/DCM: 1/1, Rf: 0.8). it was obtained as white solid (15 mg, 63%). **^{1}H NMR** (400 MHz, Chloroform-*d*) δ 7.78 (s, 1H), 7.66 (d, J = 7.3 Hz, 1H), 7.42 (d, J = 8.0 Hz, 1H), 7.30 (t, J = 7.7 Hz, 1H), 1.34 (s, 12H). **^{13}C NMR** (101 MHz, Chloroform-*d*) δ 134.56, 134.03, 132.64, 131.27, 129.19, 84.14, 24.86. The carbon directly attached to the boron atom was not detected due to quadrupolar broadening. The NMR data were in consistent with the reported data[1].

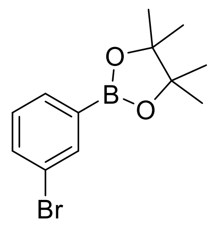

2-(3-bromophenyl)-4,4,5,5-tetramethyl-1,3,2-dioxaborolane (2t), The title compound **2t** was prepared according to the procedure and was purified by preparative thin-layer chromatography (PE/DCM: 1/1, Rf: 0.8). it was obtained as a light yellow solid (9 mg, 32%). **^{1}H NMR** (400 MHz, Chloroform-*d*) δ 7.86 (d, *J* = 1.5 Hz, 1H), 7.64 (d, *J* = 7.3 Hz, 1H), 7.53 – 7.49 (m, 1H), 7.17 (t, *J* = 8.7 Hz, 2H), 1.27 (s, 12H). **^{13}C NMR** (101 MHz, Chloroform-*d*) δ 137.47, 134.19, 133.08, 129.49, 122.45, 84.16, 24.86. The carbon directly attached to the boron atom was not detected due to quadrupolar broadening. The NMR data were in consistent with the reported data[9].

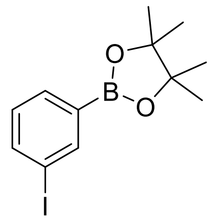

2-(3-iodophenyl)-4,4,5,5-tetramethyl-1,3,2-dioxaborolane (2u), The title compound **2u** was prepared according to the procedure and was purified by preparative thin-layer chromatography (PE/DCM: 1/1, Rf: 0.7). it was obtained as yellow solid (25 mg, 75%). **^{1}H NMR** (400 MHz, Chloroform-*d*) δ 8.07 (t, *J* = 1.5 Hz, 1H), 7.74 – 7.70 (m, 1H), 7.70 – 7.66 (m, 1H), 7.04 (t, *J* = 7.6 Hz, 1H), 1.27 (s, 12H). **^{13}C NMR** (101 MHz, Chloroform-*d*) δ 143.41, 140.10, 133.64, 129.65, 94.53, 84.15, 24.86. The carbon directly attached to the boron atom was not detected due to quadrupolar broadening. The NMR data were in consistent with the reported data[10].

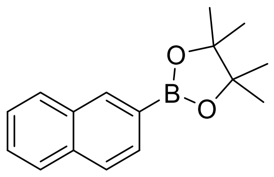

4,4,5,5-tetramethyl-2-(naphthalen-2-yl)-1,3,2-dioxaborolane (2v), The title compound **2v** was prepared according to the procudure and was purified by preparative thin-layer chromatography (PE/DCM: 1/1, Rf: 0.7). it was obtained as light yellow solid (21 mg, 85%). **^{1}H NMR** (400 MHz, Chloroform-*d*) δ 8.76 (d, J = 8.4 Hz, 1H), 8.08 (dd, J = 6.8, 1.4 Hz, 1H), 7.93 (d, J = 8.2 Hz, 1H), 7.83 (d, J = 8.4 Hz, 1H), 7.56 – 7.50 (m, 1H), 7.47 (dd, J = 8.1, 6.8 Hz, 2H), 1.42 (s, 12H). **^{13}C NMR** (101 MHz, Chloroform-*d*) δ 136.92, 135.65, 133.20, 131.61, 128.41, 128.35, 126.34, 125.48, 124.97, 83.74, 24.99. The carbon directly attached to the boron atom was not detected due to quadrupolar broadening. The NMR data were in consistent with the reported data[3].

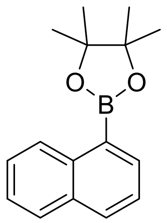

4,4,5,5-tetramethyl-2-(naphthalen-1-yl)-1,3,2-dioxaborolane (2w), The title compound **2w** was prepared according to the procudure and was purified by preparative thin-layer chromatography (PE/DCM: 1/1, Rf: 0.7). it was obtained as light yellow solid (13 mg, 50%). **^{1}H NMR** (400 MHz, Chloroform-*d*) δ 8.76 (d, J = 8.3 Hz, 1H), 8.08 (dd, J = 6.9, 1.4 Hz, 1H), 7.93 (d, J = 8.2 Hz, 1H), 7.83 (d, J = 7.8 Hz, 1H), 7.56 – 7.50 (m, 1H), 7.49 – 7.45 (m, 2H), 1.42 (s, 12H). **^{13}C NMR** (101 MHz, Chloroform-*d*) δ 136.91, 135.64, 133.20, 131.61, 128.41, 128.34, 126.34, 125.48, 124.97, 83.74, 24.99. The carbon directly attached to the boron atom was not detected due to quadrupolar broadening. The NMR data were in consistent with the reported data[1].

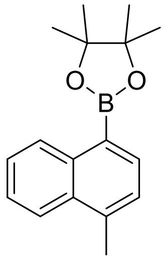

4,4,5,5-tetramethyl-2-(4-methylnaphthalen-1-yl)-1,3,2-dioxaborolane (2x), The title compound **2x** was prepared according to the procudure and was purified by preparative thin-layer chromatography (PE/DCM: 1/1, Rf: 0.8). it was obtained as light yellow solid (9 mg, 33%). **^{1}H NMR** (400 MHz, Chloroform-*d*) *δ* 8.75 – 8.69 (m, 1H), 7.96 – 7.92 (m, 1H), 7.90 (d, *J* = 7.0 Hz, 1H), 7.50 – 7.40 (m, 2H), 7.25 (d, *J* = 7.0 Hz, 1H), 2.64 (s, 3H), 1.35 (s, 12H). **^{13}C NMR** (101 MHz, Chloroform-*d*) *δ* 138.15, 136.96, 135.62, 132.89, 132.43, 128.99, 125.99, 125.33, 124.18, 83.59, 24.96, 19.95. The carbon directly attached to the boron atom was not detected due to quadrupolar broadening. The NMR data were in consistent with the reported data[11].

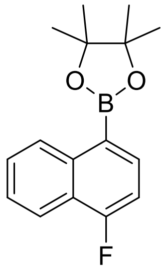

2-(4-fluoronaphthalen-1-yl)-4,4,5,5-tetramethyl-1,3,2-dioxaborolane (2y), The title compound **2y** was prepared according to the procudure and was purified by preparative thin-layer chromatography (PE/DCM: 1/1, Rf: 0.8). it was obtained as light yellow solid (20 mg, 73%). **^{1}H NMR** (400 MHz, Chloroform-*d*) δ 8.72 (d, *J* = 8.4 Hz, 1H), 8.05 (d, *J* = 8.0 Hz, 1H), 7.96 (dd, *J* = 7.5, 6.4 Hz, 1H), 7.54 – 7.49 (m, 1H), 7.49 – 7.43 (m, 1H), 7.06 (dd, *J* = 10.5, 7.8 Hz, 1H), 1.35 (s, 12H). **^{13}C NMR** (101 MHz, Chloroform-*d*) δ 161.21 (d, *J* = 256.5 Hz), 138.76 (d, *J* = 4.8 Hz), 136.16 (d, *J* = 8.8 Hz), 128.30 (d, *J* = 2.9 Hz), 127.30, 125.81 (d, *J* = 2.1 Hz), 123.53 (d, *J* = 14.9 Hz), 120.57 (d, *J* = 6.3 Hz), 108.80 (d, *J* = 18.9 Hz), 83.77, 24.97. The carbon directly attached to the boron atom

was not detected due to quadrupolar broadening. The NMR data were in consistent with the reported data[2].

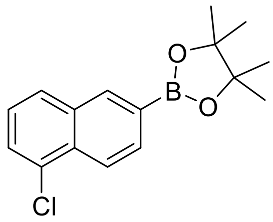

2-(5-chloronaphthalen-2-yl)-4,4,5,5-tetramethyl-1,3,2-dioxaborolane (2z), The title compound **2z** was prepared according to the procudure and was purified by preparative thin-layer chromatography (PE/DCM: 1/1, Rf: 0.7). it was obtained as light yellow solid (17 mg, 58%). **^{1}H NMR** (400 MHz, Chloroform-*d*) δ 8.30 (s, 1H), 8.16 (d, J = 8.5 Hz, 1H), 7.88 (d, J = 8.4 Hz, 1H), 7.74 (d, J = 8.2 Hz, 1H), 7.53 (d, J = 7.1 Hz, 1H), 7.31 (t, J = 7.8 Hz, 1H), 1.33 (s, 12H). **^{13}C NMR** (101 MHz, Chloroform-*d*) δ 136.41, 133.92, 132.31, 131.82, 131.66, 127.87, 127.20, 125.66, 123.43, 84.13, 24.94. The carbon directly attached to the boron atom was not detected due to quadrupolar broadening. **HRMS** (EI): m/z calculated for [$C_{16}H_{18}BClO_2$] [M+H]$^+$: 288.1083, found: 288.1081.

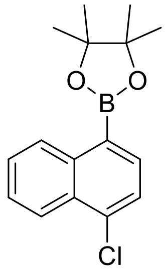

2-(4-chloronaphthalen-1-yl)-4,4,5,5-tetramethyl-1,3,2-dioxaborolane (2aa), The title compound **2aa** was prepared according to the procudure and was purified by preparative thin-layer chromatography (PE/DCM: 1/1, Rf: 0.8). it was obtained as light yellow solid (19 mg, 57%). **^{1}H NMR** (400 MHz, Chloroform-*d*) δ 8.76 – 8.70 (m, 1H), 8.26 – 8.21 (m, 1H), 7.90 (d, J = 7.5 Hz, 1H), 7.54 – 7.47 (m, 3H), 1.35 (s, 12H). **^{13}C NMR** (101 MHz, Chloroform-*d*) δ 138.09, 135.75, 135.40, 130.51, 128.81, 127.10, 126.63, 125.48, 124.55, 83.94, 24.96. The carbon directly attached to the boron atom was not detected due to quadrupolar

broadening. **HRMS** (EI): m/z calculated for [$C_{16}H_{18}BClO_2$] [M+H]$^+$: 288.1083, found: 288.1082.

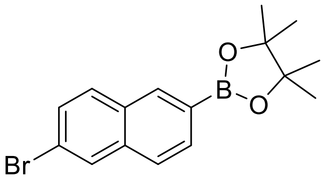

2-(6-bromonaphthalen-2-yl)-4,4,5,5-tetramethyl-1,3,2-dioxaborolane (2ab), The title compound **2ab** was prepared according to the procudure and was purified by preparative thin-layer chromatography (PE/DCM: 1/1, Rf: 0.8). it was obtained as light yellow solid (17 mg, 50%). **^{1}H NMR** (400 MHz, Chloroform-*d*) δ 8.25 (s, 1H), 7.92 (s, 1H), 7.78 (d, *J* = 8.2 Hz, 1H), 7.66 (dd, *J* = 8.4, 6.1 Hz, 2H), 7.47 (dd, *J* = 8.7, 1.9 Hz, 1H), 1.31 (s, 12H). **^{13}C NMR** (101 MHz, Chloroform-*d*) δ 136.07, 135.99, 131.51, 131.19, 130.28, 129.83, 129.27, 126.07, 121.15, 84.08, 24.94. The carbon directly attached to the boron atom was not detected due to quadrupolar broadening. **HRMS** (EI): m/z calculated for [$C_{16}H_{18}BBrO_2$] [M+H]$^+$: 332.0578, found: 332.0574.

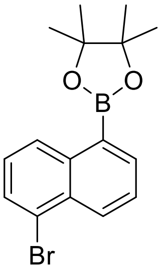

2-(5-bromonaphthalen-1-yl)-4,4,5,5-tetramethyl-1,3,2-dioxaborolane (2ac), The title compound **2ac** was prepared according to the procudure and was purified by preparative thin-layer chromatography (PE/DCM: 1/1, Rf: 0.8). it was obtained as light yellow solid (21 mg, 65%). **^{1}H NMR** (400 MHz, Chloroform-*d*) δ 8.70 (d, *J* = 8.5 Hz, 1H), 8.30 (d, *J* = 8.6 Hz, 1H), 8.05 (dd, *J* = 6.8, 1.3 Hz, 1H), 7.71 (dd, *J* = 7.4, 1.1 Hz, 1H), 7.50 (dd, *J* = 8.5, 6.9 Hz, 1H), 7.29 (dd, *J* = 8.5, 7.4 Hz, 1H), 1.35 (s, 12H). **^{13}C NMR** (101 MHz, Chloroform-*d*) δ 137.19, 135.39, 130.65, 129.53, 128.67, 127.40, 125.54, 125.37, 122.08, 82.93, 23.92.

The carbon directly attached to the boron atom was not detected due to quadrupolar broadening. **HRMS** (EI): m/z calculated for [$C_{16}H_{18}BBrO_2$] $[M+H]^+$: 332.0578, found: 332.0574.

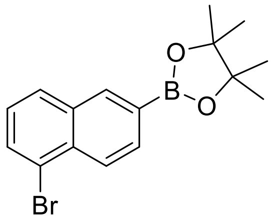

2-(5-bromonaphthalen-2-yl)-4,4,5,5-tetramethyl-1,3,2-dioxaborolane (2ad), The title compound **2ad** was prepared according to the procudure and was purified by preparative thin-layer chromatography (PE/DCM: 1/1, Rf: 0.8). it was obtained as light yellow solid (21 mg, 63%). **^{1}H NMR** (400 MHz, Chloroform-*d*) δ 8.27 (s, 1H), 8.12 (d, J = 8.5 Hz, 1H), 7.87 (d, J = 8.5 Hz, 1H), 7.78 (d, J = 8.2 Hz, 1H), 7.74 (d, J = 7.4 Hz, 1H), 7.25 (t, J = 7.8 Hz, 1H), 1.33 (s, 12H). **^{13}C NMR** (101 MHz, Chloroform-*d*) δ 136.54, 134.03, 133.52, 131.95, 130.94, 128.64, 126.15, 126.11, 122.70, 84.13, 24.94. The carbon directly attached to the boron atom was not detected due to quadrupolar broadening. **HRMS** (EI): m/z calculated for [$C_{16}H_{18}BBrO_2$] $[M+H]^+$: 332.0578, found: 332.0574.

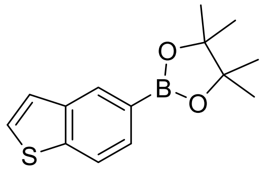

2-(benzo[b]thiophen-5-yl)-4,4,5,5-tetramethyl-1,3,2-dioxaborolane (2ae), The title compound **2ae** was prepared according to the procudure and was purified by preparative thin-layer chromatography (PE/DCM: 1/1, Rf: 0.5). it was obtained as white solid (14 mg, 53%). **^{1}H NMR** (400 MHz, Chloroform-*d*) δ 8.24 (s, 1H), 7.82 (d, J = 8.1 Hz, 1H), 7.68 (d, J = 8.0 Hz, 1H), 7.35 (d, J = 5.4 Hz, 1H), 7.28 (d, J = 5.4 Hz, 1H), 1.31 (s, 12H). **^{13}C NMR** (101 MHz, Chloroform-*d*) δ 142.70, 139.18, 130.73, 129.71, 126.01, 124.13, 121.83, 83.86, 24.92. The carbon directly attached to the boron atom was not detected due to quadrupolar broadening. The NMR data were in consistent with the reported data[12].

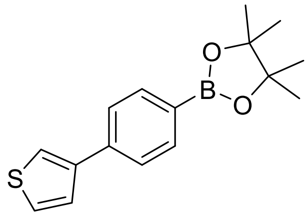

4,4,5,5-tetramethyl-2-(4-(thiophen-3-yl)phenyl)-1,3,2-dioxaborolane (2af), The title compound **2af** was prepared according to the procudure and was purified by preparative thin-layer chromatography (PE/DCM: 1/1, Rf: 0.5). it was obtained as white solid (9 mg, 30%). **^{1}H NMR** (400 MHz, Chloroform-*d*) *δ* 7.84 (d, *J* = 8.2 Hz, 1H), 7.61 (d, *J* = 8.2 Hz, 1H), 7.53 – 7.49 (m, 1H), 7.43 (dd, *J* = 5.0, 1.3 Hz, 1H), 7.39 (dd, *J* = 5.0, 2.9 Hz, 1H), 1.36 (s, 12H). **^{13}C NMR** (101 MHz, Chloroform-*d*) *δ* 142.22, 138.37, 135.33, 129.92, 129.89, 126.33, 126.25, 125.68, 120.93, 83.81, 24.89. The carbon directly attached to the boron atom was not detected due to quadrupolar broadening. The NMR data were in consistent with the reported data[3].

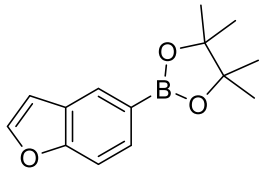

2-(benzofuran-5-yl)-4,4,5,5-tetramethyl-1,3,2-dioxaborolane (2ag), The title compound **2ag** was prepared according to the procudure and was purified by preparative thin-layer chromatography (PE/DCM: 1/1, Rf: 0.8). it was obtained as white solid (13 mg, 53%). **^{1}H NMR** (400 MHz, Chloroform-*d*) δ 8.11 (s, 1H), 7.75 (dd, *J* = 8.3, 1.3 Hz, 1H), 7.61 (d, *J* = 2.2 Hz, 1H), 7.50 (dt, *J* = 8.3, 0.9 Hz, 1H), 6.77 (dd, *J* = 2.2, 1.0 Hz, 1H), 1.37 (s, 12H). **^{13}C NMR** (101 MHz, Chloroform-*d*) *δ* 157.01, 144.92, 130.74, 128.62, 110.89, 106.65, 99.99, 83.76, 24.91. The carbon directly attached to the boron atom was not detected due to quadrupolar broadening. The NMR data were in consistent with the reported data[2].

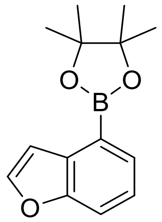

2-(benzofuran-4-yl)-4,4,5,5-tetramethyl-1,3,2-dioxaborolane (2ah), The title compound **2ah** was prepared according to the procudure and was purified by preparative thin-layer chromatography (PE/DCM: 1/1, Rf: 0.8). it was obtained as white solid (8 mg, 35%). **^{1}H NMR** (400 MHz, Chloroform-*d*) *δ* 7.71 (dd, *J* = 7.1, 1.0 Hz, 1H), 7.65 (d, *J* = 2.1 Hz, 1H), 7.59 (dt, *J* = 8.2, 1.0 Hz, 1H), 7.29 (dd, *J* = 8.2, 7.2 Hz, 1H), 7.22 (dd, *J* = 2.2, 1.0 Hz, 1H), 1.38 (s, 14H). **^{13}C NMR** (101 MHz, Chloroform-*d*) *δ* 154.24, 145.20, 132.60, 130.10, 123.56, 114.04, 108.45, 83.69, 24.99. The carbon directly attached to the boron atom was not detected due to quadrupolar broadening. The NMR data were in consistent with the reported data[13].

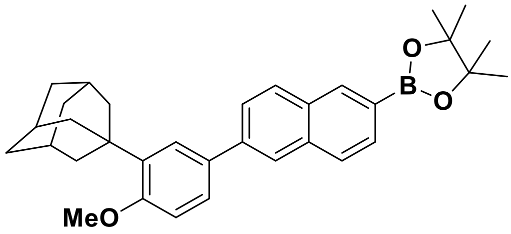

2-(6-(3-((3r,5r,7r)-adamantan-1-yl)-4-methoxyphenyl)naphthalen-2-yl)-4,4,5,5-tetramethyl-1,3,2-dioxaborolane (2ai), The title compound **2ai** was prepared according to the procudure and was purified by preparative thin-layer chromatography (PE/DCM: 1/1, Rf: 0.9). it was obtained as white solid (15 mg, 32%). **^{1}H NMR** (400 MHz, Chloroform-*d*) δ 8.30 (s, 1H), 7.90 (s, 1H), 7.85 (d, *J* = 8.5 Hz, 1H), 7.81 – 7.75 (m, 3H), 7.65 (dd, *J* = 8.5, 1.8 Hz, 1H), 7.53 (d, *J* = 2.3 Hz, 1H), 7.47 (dd, *J* = 8.4, 2.3 Hz, 1H), 6.92 (d, *J* = 8.4 Hz, 1H), 3.83 (s, 3H), 2.11 (d, *J* = 2.9 Hz, 6H), 2.03 (br, 3H), 1.76 – 1.70 (m, 6H), 1.33 (s, 12H). **^{13}C NMR** (101 MHz, Chloroform-*d*) δ 158.66, 140.01, 138.86, 135.97, 135.40, 133.06, 131.61, 130.74, 129.01, 127.13, 125.96, 125.63, 124.81, 112.07, 83.90,

55.17, 40.63, 37.20, 37.16, 29.14, 24.96. The carbon directly attached to the boron atom was not detected due to quadrupolar broadening. The NMR data were in consistent with the reported data[1].

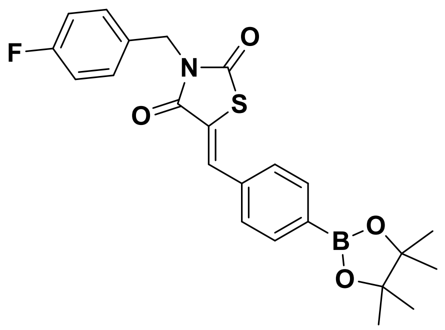

(Z)-3-(4-fluorobenzyl)-5-(4-(4,4,5,5-tetramethl-1,3,2-dioxaborolan-2-yl)benzylidene)thiazolidine-2,4-dione (2aj), The title compound **2aj** was prepared according to the procudure and was purified by preparative thin-layer chromatography (DCM/$CHCl_3$: 1/1, Rf: 0.4). it was obtained as white solid (13 mg, 30%). **^{1}H NMR** (400 MHz, Chloroform-*d*) δ 7.91 (s, 1H), 7.88 (d, J = 8.1 Hz, 2H), 7.48 (d, J = 8.1 Hz, 2H), 7.43 (dd, J = 8.6, 5.3 Hz, 2H), 7.01 (t, J = 8.7 Hz, 2H), 4.86 (s, 2H), 1.35 (s, 12H). **^{13}C NMR** (101 MHz, Chloroform-*d*) δ 167.75, 166.03, 162.65 (d, J = 246.7 Hz), 135.42, 134.10, 130.93 (d, J = 8.3 Hz), 129.27, 122.21, 115.66 (d, J = 21.6 Hz), 84.21, 44.52, 24.88. **HRMS** (ESI): m/z calculated for [$C_{23}H_{24}BFNO_4S$] [M+H]$^+$: 440.1498, found: 440.1490.

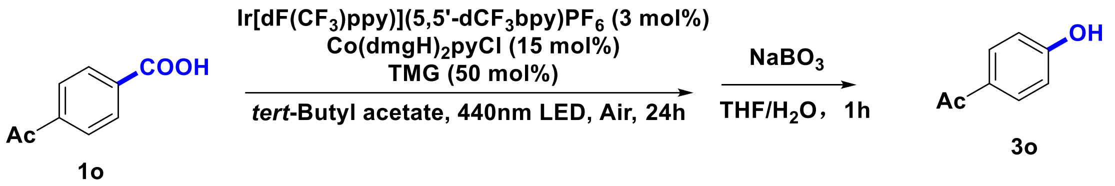

A 5 mL vial equipped with a magnetic stir bar was charged with 4- acetyl -carboxylic acid (16.5 mg, 0.1 mmol), [Ir(dF(CF_3ppy)$_2$)(5,5'-CF_3-bpy)]PF_6 (3.6 mg, 0.003 mmol), Co(dmgH)$_2$pyCl (6 mg, 0.015mmol), B_2pin_2 (39mg, 0.15 mmol), AcO^{t}Bu (2 mL) was then into the vial. The reaction mixture was stirred without irradiation for 10 min at ambient temperature, and then TMG (7 μL, 0.05 mmol) was gradually added to the vial under stirring. The reaction mixture was

stirred without irradiation for another 10 min at ambient temperature and then irradiated for 24 h while maintaining the temperature at approximately 35 °C through cooling with a fan. The solvent was removed on a rotary evaporator under reduced pressure, then $NaBO_3$ $4H_2O$ and 1:1 THF/H_2O (2 mL), and the resulting mixture was stirred at room temperature under air for 1 hour. After 1 hour, the reaction was quenched with saturated NH_4Cl (4 mL), and the contents of the round-bottom flask were transferred to a separatory funnel containing 5 mL brine using Et_2O (5 mL×2). The layers were separated, and the aqueous layer was extracted with additional Et_2O (2 x 5 mL). The combined organics were dried over Na_2SO_4, then purified by preparative thin-layer chromatography (MeOH/DCM: 1/20, Rf: 0.6). **2o'** was obtained as light yellow solid (9 mg, 64%). **^{1}H NMR** (600 MHz, Chloroform-*d*) δ 7.84 (d, *J* = 8.9 Hz, 2H), 6.83 (d, *J* = 8.9 Hz, 2H), 2.50 (s, 3H). **^{13}C NMR** (151 MHz, Chloroform-*d*) δ 197.41, 160.40, 131.04, 130.24, 115.37, 26.36.

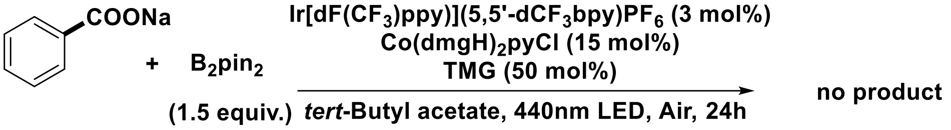

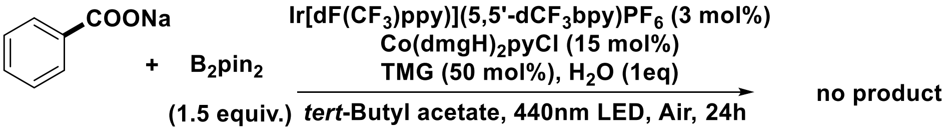

When replacing benzoic acid with sodium benzoate, no any product was monitored according to the procudure above.

4. Mechanistic Studies

4.1 Control Experiment

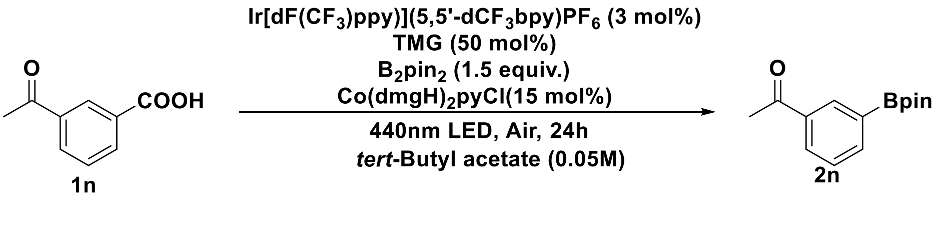

Following the above same condition but only without $Co(dmgH)_2pyCl$, or

Light, or TMG or Ir[dF(CF_3)ppy)](5,5'-dCF_3bpy)PF_6, the corresponding results are listed in the Table 11.

Supplementary Table 11. Control experiment

Conditions[a]	Result
1.Conditions	77%
2. No [Co]	49%
3. No Lght	Trace
4. No [TMG]	NR
5. No [Ir]	NR

[a]Yield was determined by HPLC with 1,3,5-trimethoxybenzene as an internal standard.

4.2 Detection of CO_2

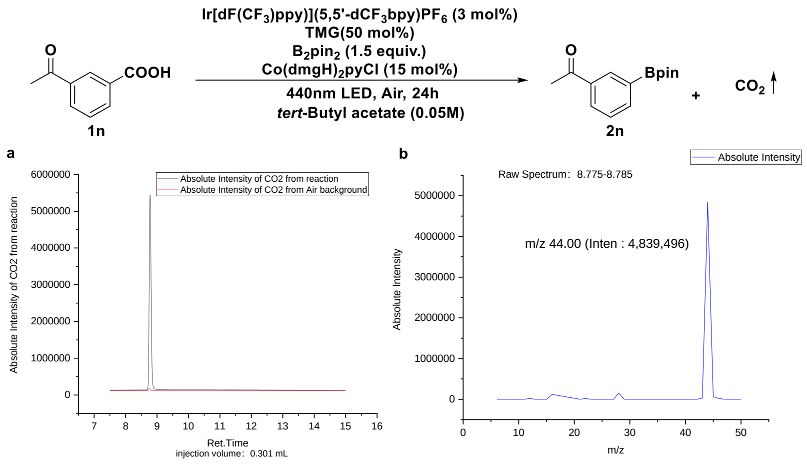

Supplementary Fig. 2. Conditions: **1** (0.1 mmol), B_2pin_2 (1.5 equiv.), [Ir-1] (3 mol%), $Co(dmgH)_2Cl_2$ (15 mol%), AcO^{t}Bu (2 mL), 440nm LED. After 24 hours, a gas sample was taken from the headspace of the sealed reaction vial via a syringe with a valve for measurement. **a** Gas chromatographic retention time of carbon dioxide. **b** Mass spectrum of carbon dioxide.

4.3 Stern-Volmer Quenching Experiment

The quenching rate k_q was determined using the Stern-Volmer relationship:

I/I_0=$\mathbf{k_q}\times\tau_0$*[quncher]*+1 (1)

Where I_0 is the fluorescence intensity without the quencher, I is the fluorescence intensity with the quencher, and ι_0 is the lifetime of the photoexcited state of the photocatalyst. The excited-state lifetime of $Ir[dF(CF_3)ppy)](5,5'\text{-}dCF_3bpy)PF_6$ in DCM is 279ns.[14]

Evaluation of 3-acetyl-benzoic acid (1n) as potential quencher for photoexcited $Ir[dF(CF_3)ppy)](5,5'\text{-}dCF_3bpy)PF_6$

Fluorescence quenching studies were carried out using a solution of $Ir[dF(CF_3)ppy)](5,5'\text{-}dCF_3bpy)PF_6$ (1×10^{-5} M), 3-acetyl-benzoic acid with different concentration (0-40 mM) in AcO*ᵗ*Bu. The samples were prepared in 4 mL quartz cuvettes, which were irradiated at 300 nm, and emission was detected at 602 nm. As shown in Fig. 3 the fluorescence intensity gradually decreased with the concentration increase of 3-acetyl-benzoic acid from 0 mM to 40 mM.

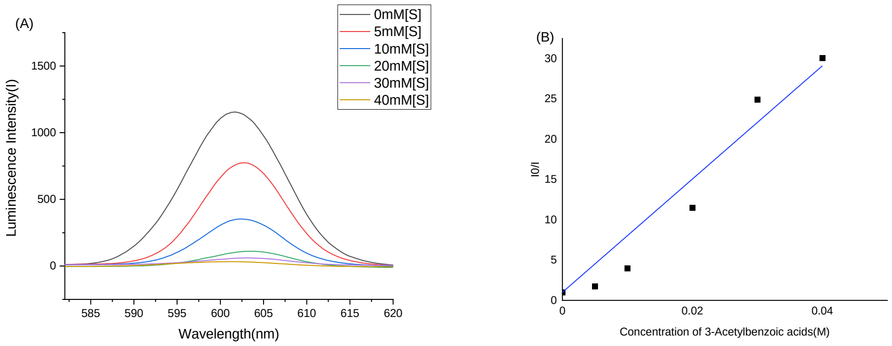

Supplementary Fig. 3. (A) The fluorescence emission spectra of $^*Ir^{(III)}$ with different concentrations of 3-acetyl-benzoic acid (**1n**). (B) Combined Stern−Volmer emission quenching data. For carboxylate, $\mathbf{k_q}$ (**1n**) $=2.5\times10^{9}$ $M^{-1}s^{-1}$.

Evaluation of $Co(dmgh)_2pyCl$ (Co-1) as potential quencher for photoexcited $Ir[dF(CF_3)ppy)](5,5'\text{-}dCF_3bpy)PF_6$

Fluorescence quenching studies were carried out using a solution of $Ir[dF(CF_3)ppy)](5,5'\text{-}dCF_3bpy)PF_6$ (1×10^{-5} M), $Co(dmgh)_2pyCl$ with different concentration (0-88 μM) in AcOᵗBu. The samples were prepared in 4 mL quartz cuvettes, which were irradiated at 300 nm, and emission was detected at 602

nm. As shown in Fig. 4, the fluorescence intensity dramatically decreased with the concentration increase of $Co(dmgh)_2pyCl$ from 0 mM to 88 μM.

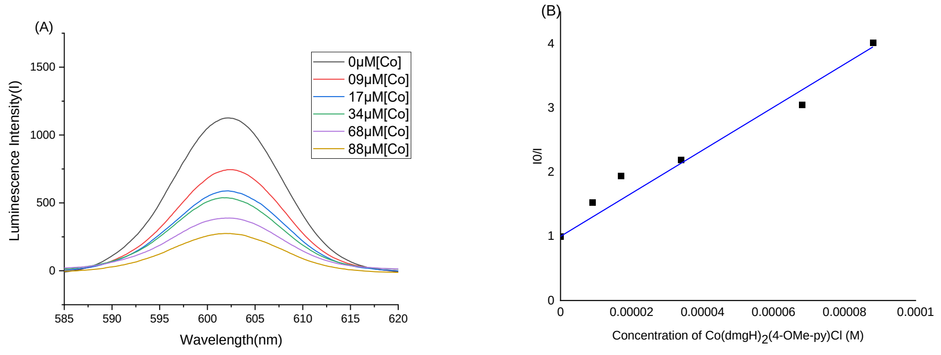

Supplementary Fig. 4. (A) The fluorescence emission spectra of *Ir(III) with different concentration of $Co(dmgh)_2pyCl$; (B) Combined Stern−Volmer emission quenching data. For $Co(dmgh)_2pyCl$, $\mathbf{k_q}$ (**Co-1**) $=1.2\times10^{11} M^{-1}s^{-1}$.

Evaluation of TMG as potential quencher for photoexcited Ir[dF(CF$_3$)ppy)](5,5'-dCF$_3$bpy)PF$_6$

Fluorescence quenching studies were carried out using a solution of Ir[dF(CF$_3$)ppy)](5,5'-dCF$_3$bpy)PF$_6$ (1×10^{-5} M), TMG with different concentration (0-60 μM, 0-0.6 mM) in AcO^{t}Bu. The samples were prepared in 4 mL quartz cuvettes, which were irradiated at 300 nm, and emission was detected at 602 nm. As shown in Fig. 5, no quenching was observed.

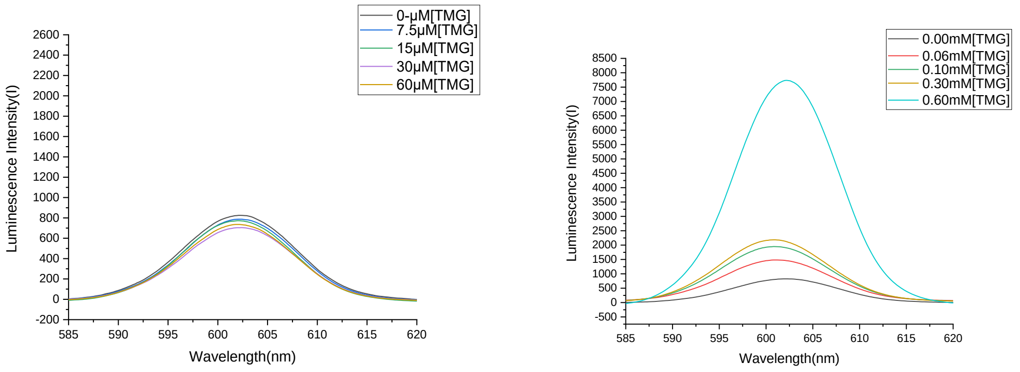

Supplementary Fig. 5. The fluorescence emission spectra of *Ir$^{(III)}$ with different concentrations of TMG; No quenching was observed.

4.4 Synthesis and conversion of the intermediate R-Co(III)

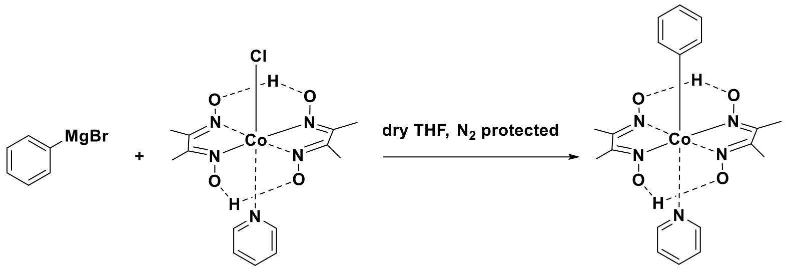

PhCo(dmgH)$_2$py was synthesized according to literature.[15] ^{1}**H NMR** (400 MHz, Chloroform-*d*) δ 8.71 – 8.66 (m, 2H), 7.68 (tt, J = 7.6, 1.6 Hz, 1H), 7.35 – 7.26 (m, 4H), 6.91 – 6.78 (m, 3H), 3.42 (s, 2H), 1.96 (s, 12H). The ^{1}**HNMR** data were in consistent with the reported data.

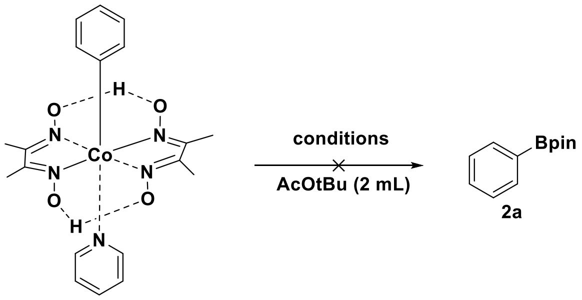

A 5 mL vial equipped with a magnetic stir bar was charged with PhCo(dmgH)$_2$py (22 mg, 0.05 mmol), anhydrous AcO^{t}Bu solvent (2 mL), B_2pin_2 (1.5 equiv.), and different conditions (listed below table) were conducted, no related product **2a** was detected, excluding the possible intermediates.

Supplementary Table 12. Condition screening on the conversion of the intermediate Ph-Co(III)

Conditions^d	Result
1. B_2pin_2 (1.5 equiv.), RT	NR
2. B_2pin_2 (1.5 equiv.), 40°C	NR
3. B_2pin_2 (1.5 equiv.), TMG (50 mol%), 40°C	NR
4. B_2pin_2 (1.5 equiv.), TMG (50 mol%), [Ir] (3 mol%), Light (440nM LED)	NR
5. B_2pin_2 (1.5 equiv.), TMG (50 mol%), HOAc (50 mol%), 40°C	NR
6. B_2pin_2 (1.5 equiv.), TMG (50 mol%), HOAc (50 mol%), [Ir] (3 mol%), Light (440nM LED)	NR

4.5 Radical Trapping or Inhibiting Experiments

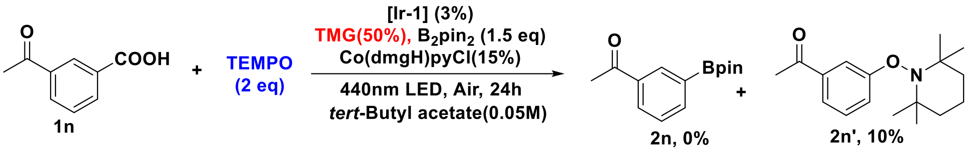

Following the standard borylated condition with TEMPO (31 mg, 0.2 mmol), the **2ai'** was acquired with a 10% yield, and no **2n** was detected.

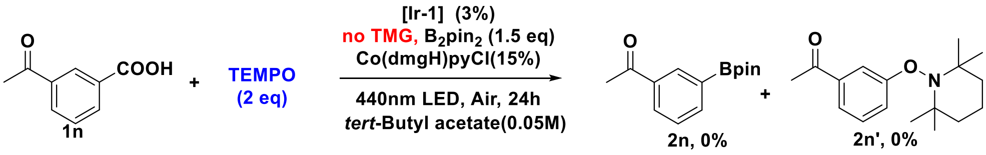

Following the above same radical inhibiting experiments without TMG, no **2n** and the captured product of TEMPO **2n'** were detected.

4.6 *in situ* ^{1}HNMR Analysis

Condition 1: A 5 mL vial equipped with a magnetic stir bar was charged with 3-acetyl-benzoic acid (5 mg, 0.033 mmol), anhydrous AcO^{t}Bu solvent (1 mL); The vial was sealed and shook for 1 minute to make solid dissolved. 0.5 ml of the above were conducted in screw-cap NMR tubes; a Capillary tube containing pure C_6D_6 was placed in each of the NMR tubes to allow locking and shimming for NMR experiments. The data was shown as curve 1 in Fig. 6.

Condition 2: A 5 mL vial equipped with a magnetic stir bar was charged with TMG (2 μL, 0.016 mmol), anhydrous AcO^{t}Bu solvent (1 mL); The vial was sealed and shook for 1 minute to make solid dissolved. The ^{1}H NMR analysis was conducted with the same condition as condition 1 and the data was shown in curve 2 in Fig. 6.

Condition 3: A 5 mL vial equipped with a magnetic stir bar was charged with 3-acetyl-benzoic acid (5 mg, 0.033 mmol), TMG (2 μL, 0.016 mmol), anhydrous AcO^{t}Bu solvent (1 mL); The vial was sealed and shaken for 1 minute to make solid dissolved. The ^{1}H NMR analysis was conducted with the same

condition as condition 1 and the data was shown in curve 3 in Fig. 6.

Condition 4: A 5 mL vial equipped with a magnetic stir bar was charged with 3-acetyl-benzoic acid (5 mg, 0.033 mmol), TMG (4 μL, 0.032 mmol), anhydrous AcO^tBu solvent (1 mL); The vial was sealed and shook for 1 minute to make solid dissolved. The ^{1}H NMR analysis was conducted with the same condition as condition 1 and the data was shown in curve 4 in Fig. 6.

Condition 5: A 5 mL vial equipped with a magnetic stir bar was charged with 3- acetyl -carboxylic acid (16.5 mg, 0.1 mmol), $[Ir(dF(CF_3ppy)_2)(5,5'-CF_3-bpy)]PF_6$ (3.6 mg, 0.003 mmol), $Co(dmgh)_2pyCl$ (6 mg, 0.015mmol), B_2pin_2 (39mg, 0.15 mmol), AcO^tBu (2 mL) was then into the vial. The reaction mixture was stirred without irradiation for 10 min at ambient temperature, and then TMG (7 μL, 0.05 mmol) was gradually added to the vial under stirring. The reaction mixture was stirred without irradiation for another 10 min at ambient temperature and then irradiated for 5 h while maintaining the temperature at approximately 35 °C through cooling with a fan. The ^{1}H NMR analysis was conducted with the same condition as condition 1 and the data was shown in curve 5 in Fig. 6.

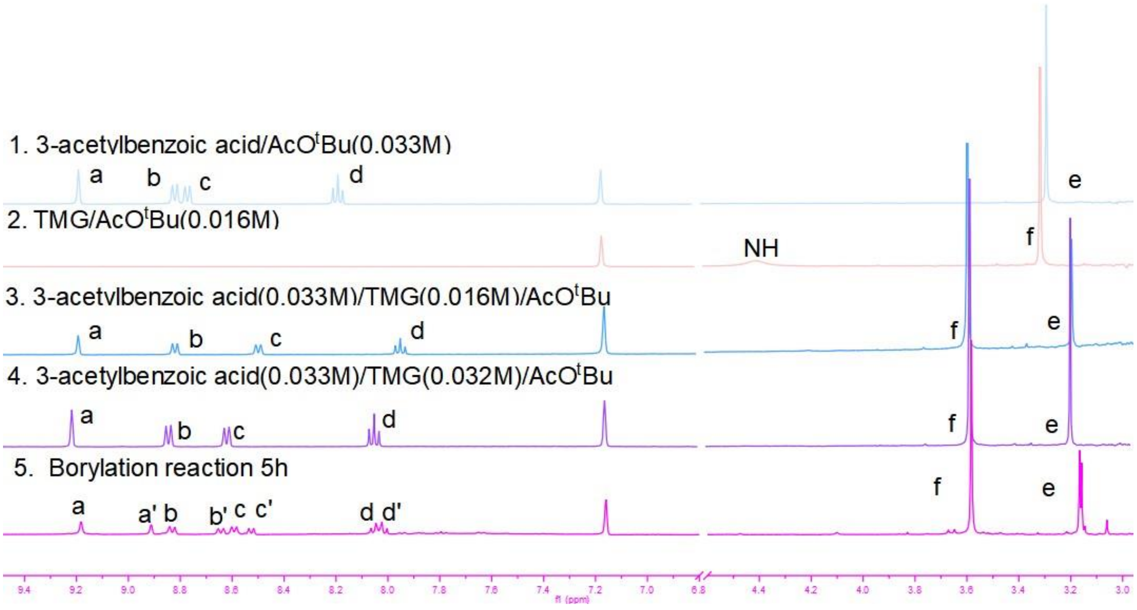

Supplementary Fig. 6. *in situ* ^{1}HNMR analysis

4.6 *in situ* ^{11}BNMR Analysis

Condition 1: A 5 mL vial equipped with a magnetic stir bar was charged with B_2pin_2 (12.5 mg, 0.1 mmol), anhydrous MeCN solvent (1 mL); The vial was sealed and shook for 1 minute to make solid dissolved. 0.5 ml of the above were conducted in screw-cap NMR tubes; a Capillary tube containing pure C_6D_6 was placed in each of the NMR tubes to allow locking and shimming for NMR experiments. The data was shown as curve 1 in Fig. 7.

Condition 2: A 5 mL vial equipped with a magnetic stir bar was charged with B_2pin_2 (12.5 mg, 0.1 mmol), anhydrous Ethyl acetate solvent (1 mL); The ^{11}B NMR analysis was conducted with the same condition as condition 1 and the data was shown as curve 2 in Fig. 7.

Condition 3: A 5 mL vial equipped with a magnetic stir bar was charged with B_2pin_2 (12.5 mg, 0.1 mmol), TMG (2 μL, 0.016 mmol), anhydrous AcO^tBu (1 mL); The ^{11}B NMR analysis was conducted with the same condition with condition 1 and the data was shown as curve 3 in Fig. 7.

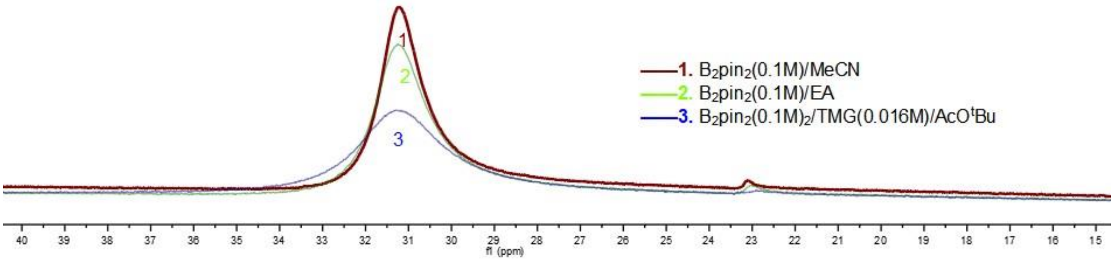

Supplementary Fig. 7. *in situ* ^{11}B NMR analysis

4.7 Determination of Quantum Yield (standard ferrioxalate actinometry)

4.7.1 Determination of Photon Flux of the Reactor

The photon flux of blue LED was determined by standard ferrioxalate actinometry[16-18].

1. A 0.15 M solution of ferrioxalate was prepared by dissolving potassium ferrioxalate hydrate (368.4 mg, 0.750 mmol) in 5.0 mL of 0.20 M aqueous

sulfuric acid. A 0.15 M buffered solution of 1,10-phenanthroline was prepared by dissolving 1,10-phenanthroline (541 mg, 3.00 mmol) and sodium acetate (1.23 g, 15.0 mmol) in 20 mL of 0.20 M aqueous sulfuric acid.

2. To 4 mL, a quartz cuvette equipped with a magnetic stir bar was added 0.50 mL of the ferrioxalate solution. The quartz cell was sealed and placed 2 cm away from the blue LEDs. After irradiation for different seconds, 1.5 mL of the aqueous sulfuric acid and 2.0 mL of the buffered solution were added to the vial (V_1=0.5+1.5+2 mL). after 1 hour, 45 μL (V_2) of the above solution was taken as an aliquot and diluted with 3.0 mL (V_3) of 0.20 M aqueous sulfuric acid; then 1mL (V_4) of the resulting solution was taken as an aliquot and diluted with 3.0 mL (V_5) of 0.20 M aqueous sulfuric acid.

3. The absorbance of the resulting solution in a quartz cuvette (l = 1.0 cm) at 510 nm was measured by a UV-Vis spectrometer. A non-irradiated sample and other samples with 5s, 15s, 30s, 45s, 60s, and 90s irradiation time were also prepared, and the absorbance at 510 nm was measured.

The amount of ferrous ion formed was calculated as follows: *Mol* Fe^{2+} = $V_1V_3V_5\Delta A_{510} / \varepsilon_{510} l\ V_2V_4$ (2)

Supplementary Table 13. Raw chemical actinometry data to determine the photon flux

irradiation time(s)	Absorbance	ΔA	Mol Fe^{2+} (mol)
non-irradiation	0.005		
5	0.011	0.006	4.33×10^{-7}
15	0.021	0.016	1.15×10^{-6}
30	0.030	0.025	1.80×10^{-6}
45	0.037	0.032	2.31×10^{-6}
60	0.051	0.046	3.32×10^{-6}
90	0.069	0.064	4.61×10^{-6}

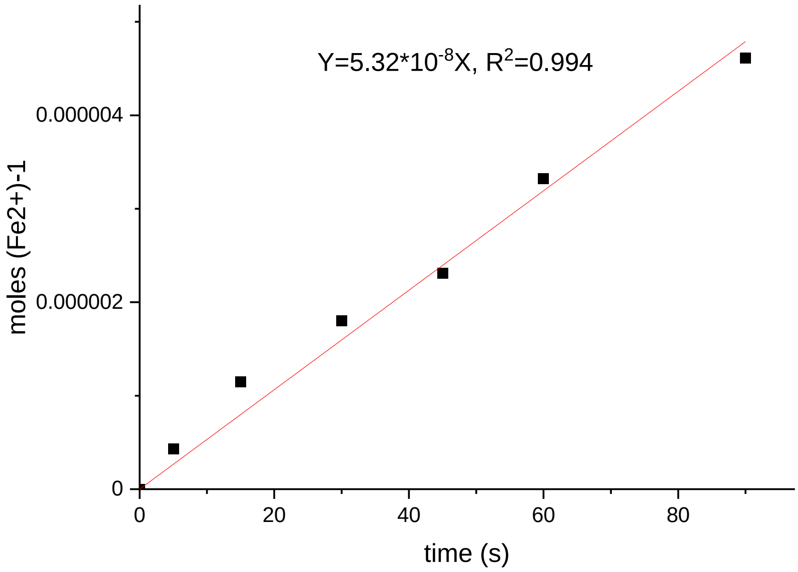

Supplementary Fig. 8. Compiled linear fits for the photon flux

Photon flux may be determined by: *photon flux = Mol* Fe^{2+}/Φ t f (3)

where **Φ** is the quantum yield for the ferrioxalate actinometer (approximated as 0.845, which was reported for a 0.15 M solution at λ = 457.9 nm)[19], t is the irradiation time, and f is the fraction of light absorbed at 440 nm (the mean fraction of light absorbed by the ferrioxalate solution approximately equal to 1 at 440 nm at 0.15 M ferrioxalate).

The value of the slope collected is 5.32×10^{-8}; division by the known quantum yield **Φ** = 0.845 yields a photon flux of 6.3×10^{-8} einsteins s^{-1}.

4.7.2 Determination of the Reaction Quantum Yield

To a 4 mL a quartz cuvette equipped with a magnetic stir bar was charged with 3-acetyl -carboxylic acid (16.5 mg, 0.1 mmol), [Ir(dF(CF_3ppy)$_2$)(5,5'-CF_3-bpy)]PF_6 (3.6 mg, 0.003 mmol), Co(dmgh)$_2$pyCl (6 mg, 0.015mmol), B_2pin_2 (39mg, 0.15 mmol), AcO^{t}Bu (2 mL). The reaction mixture was stirred without irradiation for 10 min at ambient temperature and then TMG (7 μL, 0.05 mmol) was gradually added to the vial under stirring. The reaction mixture was stirred without irradiation for another 10 min at ambient temperature, and then placed 2 cm away from the blue LEDs and irradiated for 1 h while maintaining the

temperature at approximately 35 °C through cooling with a fan. 1,3,5-trimethoxybenzene (HPLC internal standard quantification based on the internal standard 1,3,5-trimethoxybenzene, the peak area and relative correction factor of reaction product) was added then the mixture was analyzed by HPLC. and the 32% yield of product **2n** (3.2×10^{-5} mol) was determined.

the absorbance of the solution at 450 nm was 3, f = $1.0000 - 10^{-A} \approx 1$ (A = 3.987)

Φ = mol product/flux×t×f = 3.2×10^{-5} mol/ 3600s × 6.3×10^{-8} Einstein/s ×1=0.14.

4.8. Computational Study

All density functional theory (DFT) calculations were carried out with the Gaussian 16, Revision A.03. The B3LYP functional is mainly used in our research (CAM-B3LYP, M06, PBE1PBE are also used to check the validity of the computational method), and the 6-311+g(d,p) basis set was used for organic molecules, For metal-containing molecules, light elements (C,H,N,O etc) were treated using the 6-31G* basis set while metal atoms (Co and Ir) were treated using the LanL2DZ/SDD basis set. For all the calculations, Grimme-D3 correction was utilized for the Empirical Dispersion, [S. Grimme, J. Antony, S. Ehrlich and H. Krieg, *J. Chem. Phys.*, **132** (2010) 154104] and the solvent effect of ethyl-ethanoate was included with Truhlar's SMD model [A. V. Marenich, C. J. Cramer, and D. G. Truhlar, *J. Phys. Chem. B*, **113** (2009) 6378-96].

4.8.1. Calculation of redox potential

The redox potentials were obtained from the computation of oxidation-reduction half-reactions, according to

$$\Delta G = -\mathrm{n}\mathrm{F}E, \quad (4)$$

$$E^0\ (\text{versus SCE}) = -(\Delta G_{red}/nF) - E(\text{SCE}), \quad (5)$$

$E(\mathrm{SCE}) = 4.6812\mathrm{V}$.[20]

Where ΔG is the free energy change (J/mol), n is the number of electrons transfer in oxidation-reduction half-reaction, F = 96485 J mol^{-1} V^{-1} is the Faraday constant, E is the oxidation-reduction half-reactions' potential. $E(\mathrm{SCE})$ is the potential of Saturated Calomel Electrode.

The Gibbs free energies were computed through the DFT and TDDFT calculation with frequency analysis.

Supplementary Table 14. The computed ΔG and $E^0_{\mathrm{red}}(\mathrm{versus\ SCE})$ for the redox half-reactions of different molecules and complexes

	ΔG(kcal/mol)	$E^0_{\mathrm{red}}(\mathrm{versus\ SCE})(\mathrm{V})$
**1b''**/ PhCOOH	-165.9	2.51
$PhCOONa^{+}$/ PhCOONa	-149.9	1.82
PhCOO•/ $PhCOO^{-}$	-120.4	0.54
**1b'/ 1a** in Oxidation-HAT	-140.0	1.39
**1d'/ 1c** in PT-Oxidation	-139.2	1.36
**1b'-1/ 1a-1** in Oxidation-HAT	-141.7	1.46
**1d'-1/ 1c-1** in PT-Oxidation	-140.3	1.40
**1b'-2/ 1a-2**	-134.1	1.13
**1b'-3/ 1a-3**	-140.4	1.41
$[\mathrm{Ir\text{-}1}]^{\mathrm{IV/III}}$	-146.9	1.69
$[\mathrm{Ir\text{-}7}]^{\mathrm{IV/III}}$	-131.0	1.00
$[\mathrm{Ir\text{-}1}]^{\mathrm{IV/III*}}$	-98.4	-0.42
$[\mathrm{Ir\text{-}7}]^{\mathrm{IV/III*}}$	-78.8	-1.27
$[\mathrm{Ir\text{-}1}]^{\mathrm{III/II}}$	-82.8	-1.09
$[\mathrm{Ir\text{-}7}]^{\mathrm{III/II}}$	-65.5	-1.84
$[\mathrm{Ir\text{-}1}]^{\mathrm{III*/II}}$	-131.3	1.01
$[\mathrm{Ir\text{-}7}]^{\mathrm{III*/II}}$	-113.6	0.25
$[\mathrm{Co\text{-}1}]^{\mathrm{III/II}}$	-83.3	-1.07

Note: Ir^{III*} is computed on Ir^{III} 's lowest triplet excited state T_1 optimization structure by TDDFT.

Note: **1a-1**, **1a-2**, **1a-3** are other possible conformers of the complex of benzoic acid and TMG, details are listed in 4.8.6.

Discussion

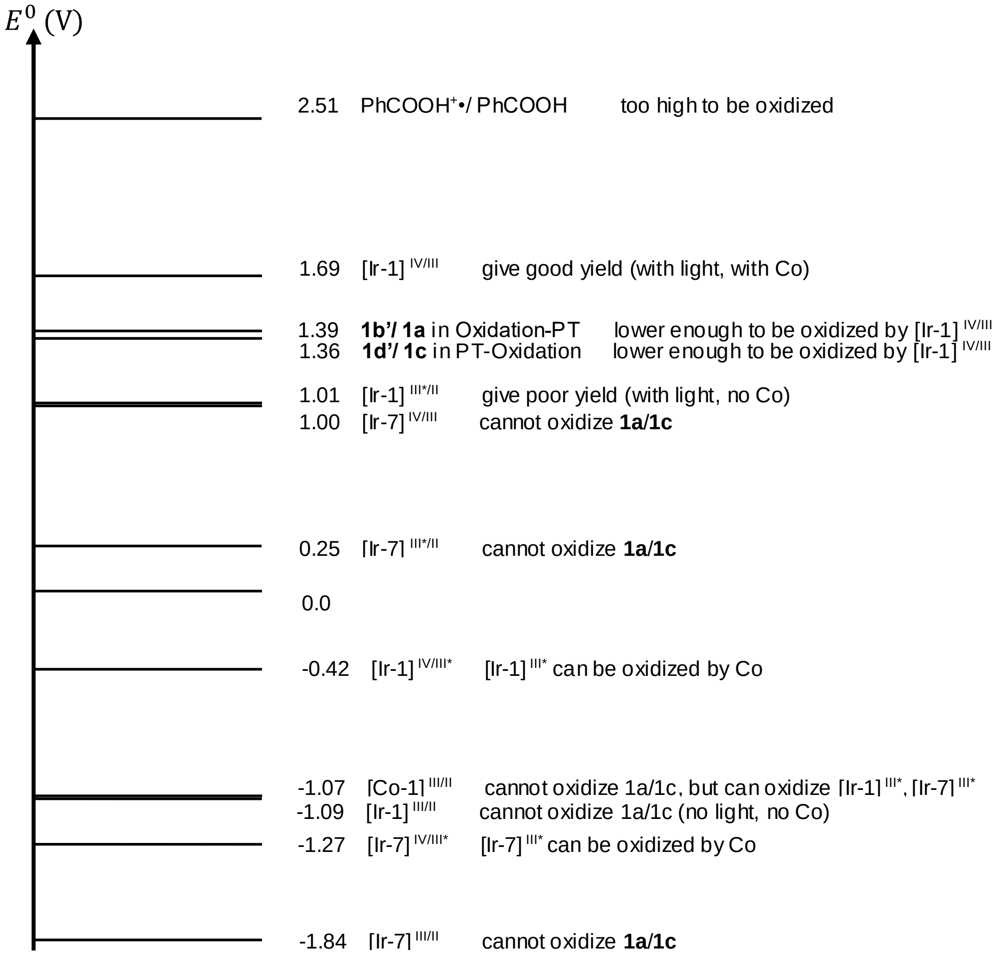

The computation data above does tell the effects of TMG, which is lowering the redox potential of benzoic acid to **1a**/**1c** for about 1.12V/1.15V, and the effects of photo-inducing by lowering the redox potential of [Ir-1] $^{IV/III}$ to [Ir-1] $^{IV/III*}$ for about 2.1V, which drives the [Co-1] ^{III} oxidize [Ir-1]^{III*} to [Ir-1]^{IV} for further reaction.

To check the validity of the computational method, we have utilized other functionals (CAM-B3LYP, M06, PBE1PBE) and bigger basis (SDD), and received similar results (showed in Table 15 below).

Supplementary Table 15. $E^0_{\mathrm{red}}(\mathrm{versus\ SCE})$ for the redox half-reactions of [Ir-1], [Ir-7], and [Co-1], utilizing various functionals and LanL2DZ, SDD basis sets for metal elements, 6-31g* for other elements.

$E^0_{\mathrm{red}}(\mathrm{V})$	B3LYP		CAM-B3LYP		M06		PBE1PBE	
	LanL2DZ	SDD	LanL2DZ	SDD	LanL2DZ	SDD	LanL2DZ	SDD
$[\mathrm{Ir\text{-}1}]^{\mathrm{IV/III}}$	1.69	1.71	1.95	1.97	1.73	1.77	1.77	1.79
$[\mathrm{Ir\text{-}1}]^{\mathrm{IV/III*}}$	-0.42	-0.41	-0.32	-0.30	-0.47	-0.50	-0.50	-0.51
$[\mathrm{Ir\text{-}1}]^{\mathrm{III/II}}$	-1.09	-1.07	-1.17	-1.15	-1.10	-1.10	-1.08	-1.08
$[\mathrm{Ir\text{-}1}]^{\mathrm{III*/II}}$	1.01	1.05	1.11	1.12	1.10	1.17	1.19	1.21
$[\mathrm{Ir\text{-}7}]^{\mathrm{IV/III}}$	1.00	1.17	1.14	1.17	1.04	1.08	1.10	1.12
$[\mathrm{Ir\text{-}7}]^{\mathrm{IV/III*}}$	-1.27	-1.21	-1.23	-1.21	-1.30	-1.32	-1.33	-1.33
$[\mathrm{Ir\text{-}7}]^{\mathrm{III/II}}$	-2.02	-2.06	-2.07	-2.06	-1.95	-1.94	-1.95	-1.94
$[\mathrm{Ir\text{-}7}]^{\mathrm{III*/II}}$	0.25	0.31	0.30	0.31	0.39	0.46	0.49	0.51
$[\mathrm{Co\text{-}1}]^{\mathrm{III/II}}$	-1.07	-1.19	-1.00	-1.13	-1.14	-1.14	-1.12	-1.22

4.8.2. Calculation of the activation energy of the redox reaction

According to Marcus theory, the energy barrier of the reaction, i.e. the activation energy can be calculated as following:

$$\Delta G^{\ddagger} = \frac{(\lambda_0 + \Delta G^0)^2}{4\lambda_0}, \qquad (6)$$

Where λ_0 is the reorganization energy and ΔG^0 is the energy change of reaction. λ_0 and ΔG^0 can be calculated from adiabatic state energies at the optimized structure of the initial and final states. Because of the complexity to treat the whole redox reaction with the donor-acceptor complex, we computed the two half reactions separately first, and then combined the results to get the approximate result. (showed in Table 16 and 17 below)

Supplementary Table 16. Reorganization energy λ_0, energy change ΔG^0, and the activation energy $\Delta G^{\ddagger}$ of the half redox reactions are listed below

	Reduction		Oxidization		
	$[\mathrm{Ir\text{-}1}]^{\mathrm{IV}}$	$[\mathrm{Ir\text{-}7}]^{\mathrm{IV}}$	PhCOOH	**1a**	**1c**
λ_0(a.u.)	0.0030	0.0033	0.0073	0.0176	0.0218
ΔG^0(a.u.)	-0.2300	-0.2047	0.2686	0.2193	0.2262

Supplementary Table 17. Reorganization energy λ_0, energy change ΔG^0, and the activation energy $\Delta G^‡$ of the the combined whole redox reactions are listed below.

	[Ir-1] ^{IV}			[Ir-7] ^{IV}		
	PhCOOH	**1a**	**1c**	PhCOOH	**1a**	**1c**
λ_0(a.u.)	0.0103	0.0206	0.0248	0.0106	0.0209	0.0252
ΔG^0(a.u.)	0.0387	-0.0106	-0.0038	0.0639	0.0146	0.0214
ΔG^0(kcal/mol)	24.27	-6.66	-2.38	40.10	9.16	13.44
$\Delta G^‡$(a.u.)	0.058	0.001	0.004	0.131	0.015	0.022
$\Delta G^‡$(kcal/mol)	36.50	0.76	2.80	81.93	9.46	13.53

The computed results shown above once again confirms that TMG group is essential to promote the redox reaction, by lowering both ΔG^0 (thermodynamics) and $\Delta G^‡$ (kinetics). In particular, the $\Delta G^‡$ of the [Ir-1] ^{IV} **1a/1c** redox reaction is much smaller than the ones of [Ir-7] ^{IV} (0.76/2.80 kcal/mol vs 9.46/13.53 kcal/mol), agreeing well with the big difference of yield between these two catalysts.

Since the rate of proton transfer (**1a** to **1c**) is fast, and the reverse proton transfer (**1c** to **1a**) rate is also fast, at ground state **1a** and **1c** could reach a Boltzmann distribution first, and then proceed the redox reaction. According to the low activation energy of both redox paths (0.76 kcal/mol for **1a**, 2.80 kcal/mol for **1c**) and the limitation of computational accuracy, we conclude that Oxidation-HAT process and PT-Oxidation process could both contribute to the TMG-assisted benzoic acid oxidation reaction effectively.

4.8.3. Molecular orbital analyses on radical forming

To further understand the role of TMG to reduce the redox potential, we compare the involved molecular orbitals (MOs) of the benzoic acid and benzoic acid-TMG complex.

From the comparison of the MOs, it is clear that the main difference comes from the Singly-Occupied MOs (SOMOs) for the corresponding radical. For complex 1a and 1c, the SOMO is much delocalized to the TMG molecules, and the orbital energies are decreased comparing to the one of isolated benzoic

acid.

Supplementary Table 18. Comparing the orbital of SOMO that one electron excited, and their corresponding electron energy (eV)

	SOMO	Orbital energy /eV
PhCOOH	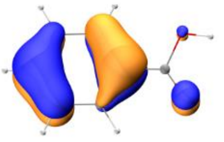	-7.72
Complex **1c**	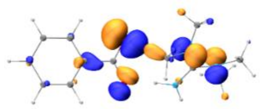	-6.78
Complex **1a**	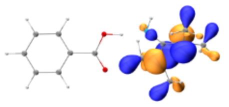	-6.53
TMG	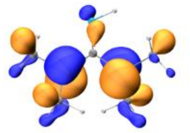	-6.34

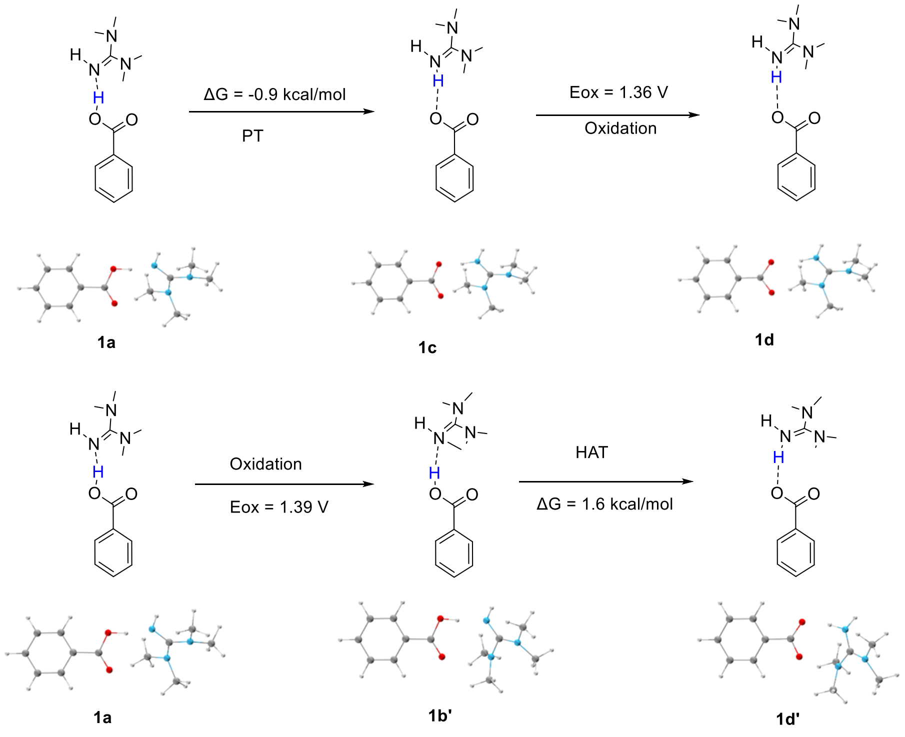

Supplementary Fig. 9. The energy profile for the activation of benzoic acid by TMG

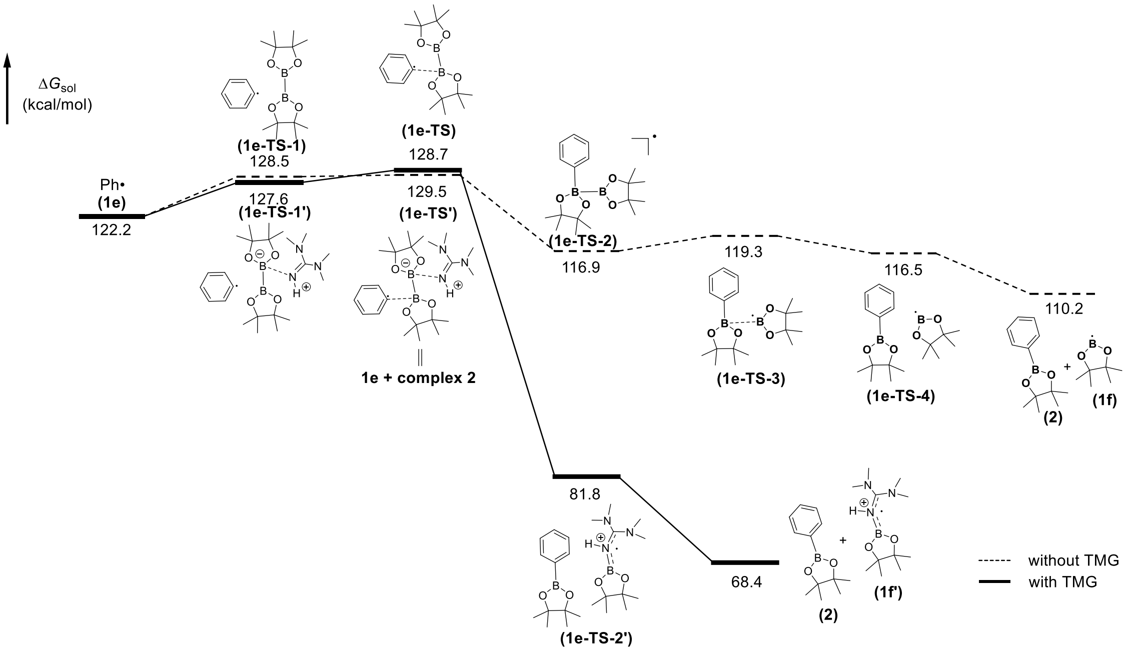

Supplementary Fig. 10. Energy profile for complete borylation

Supplementary Table 19. The possible conformers of borylation

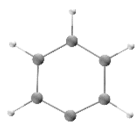 **1e**	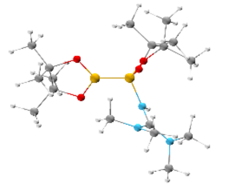 **complex2**	
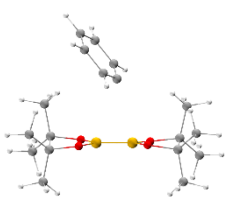 **1e-TS-1**	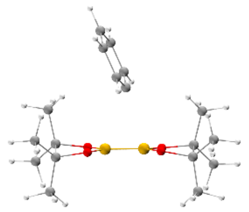 **1e-TS**	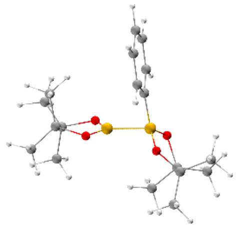 **1e-TS-2**
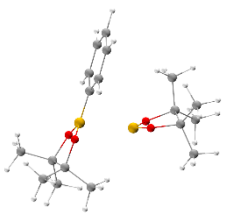 **1e-TS-3**	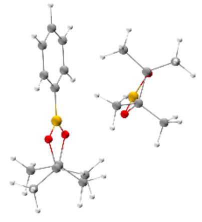 **1e-TS-4**	

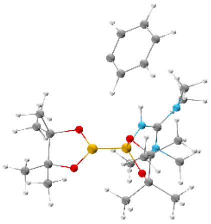 **1e-TS-1’**	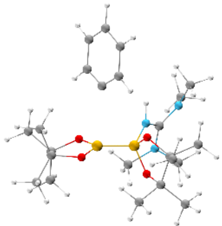 **1e-TS’**	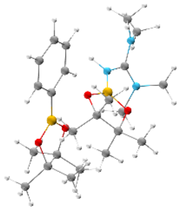 **1e-TS-2’**
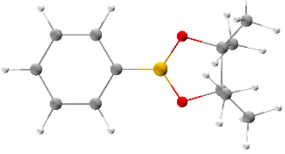 **2**	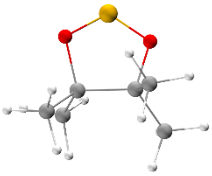 **1f**	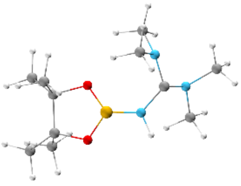 **1f’**

Supplementary Table 20. Other possible conformers and binding energy.

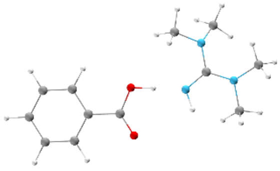 **1a-1**	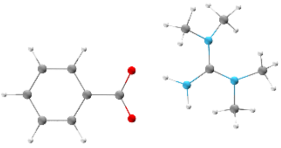 **1c-1**	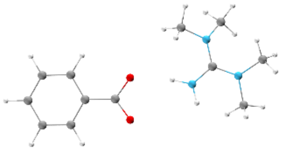 **1d-1**
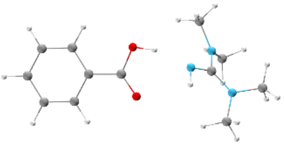 **1b’-1**	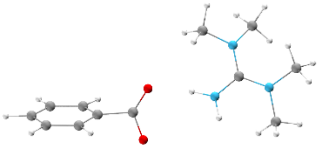 **1d’-1**	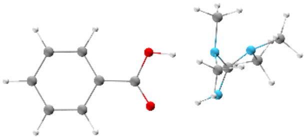 **1a-2**
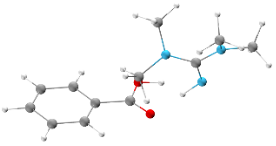 **1b’-2**	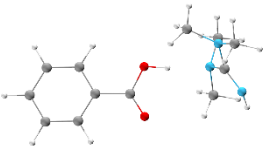 **1a-3**	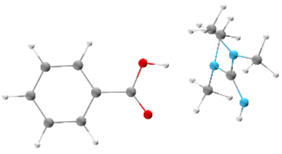 **1b’-3**

Supplementary Table 21. Binding energy of potential reactants of the decarboxylation reaction

	binding energy (kcal/mol)
**1a**	-3.3

**1c**	-13.7
**1b'**	3.4
**1d**	5.1
**1d'**	8.5
PhCOONa	-39.2
**1a-1**	-4.2
**1c-1**	-14.6
**1a-2**	6.5
**1a-3**	3.4

According to the data above, complex **1a**,**1a-1** are able to form, and then proceed proton transfer to form **1c**, **1c-1** respectively. There is only small structure and energy difference between **1a-1**,**1c-1** and **1a**,**1c,** caused by the rotation of the TMG group.

Since the redox potential of **1a**, **1c** (1.39V, 1.36V) are both lower than those of **1a-1**, **1c-1** (1.46V, 1.40V), implying more improvement caused by TMG. We picked **1a**, **1c** as the representatives of the mechanism in our research.

For complex **1a-2** and **1a-3**, they are not easy to form because of their positive binding energy.

Spectra

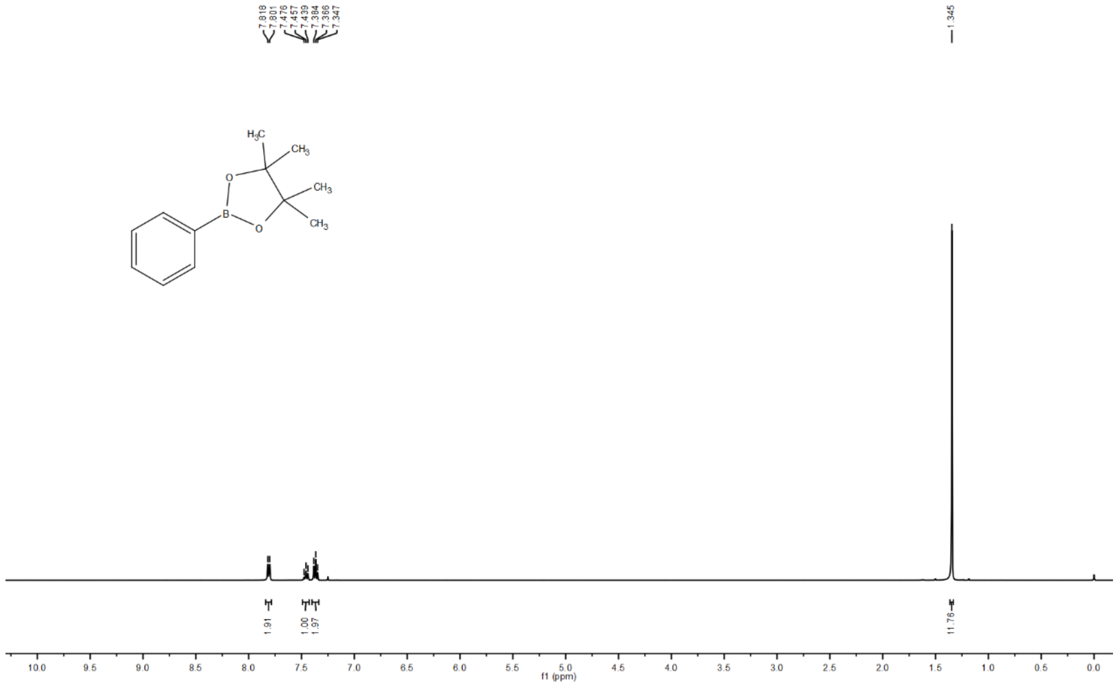

Supplementary Fig. 11. ^{1}H NMR (400 MHz, 298 K, $CDCl_3$) of **2a**

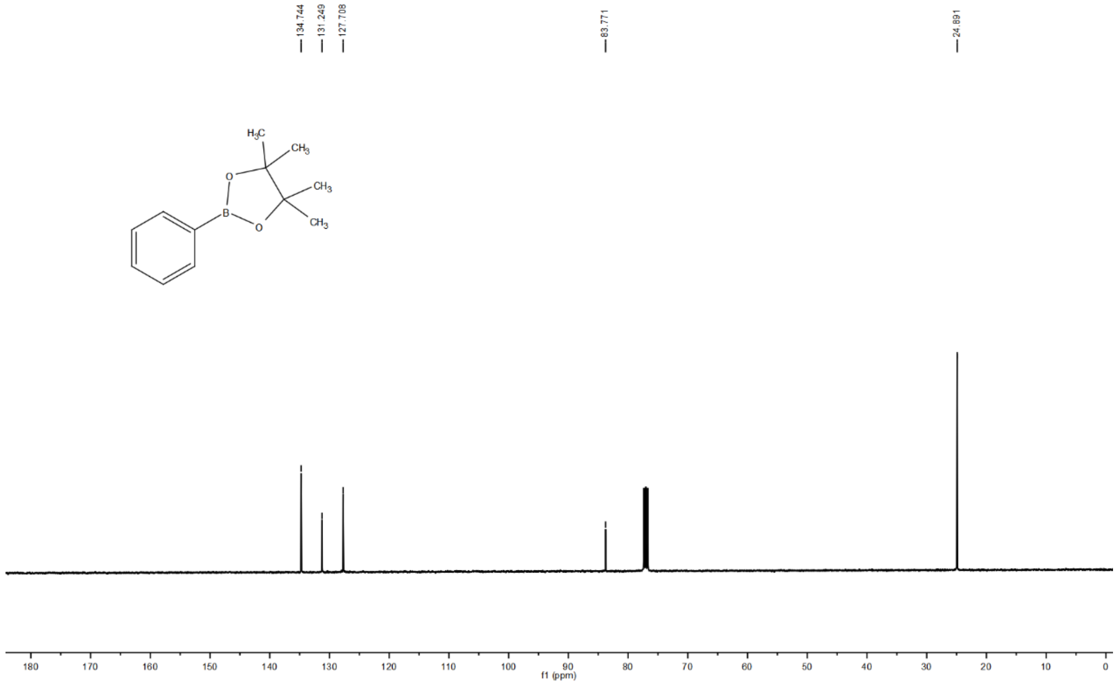

Supplementary Fig. 12. ^{13}C NMR (101 MHz, 298 K, $CDCl_3$) of **2a**

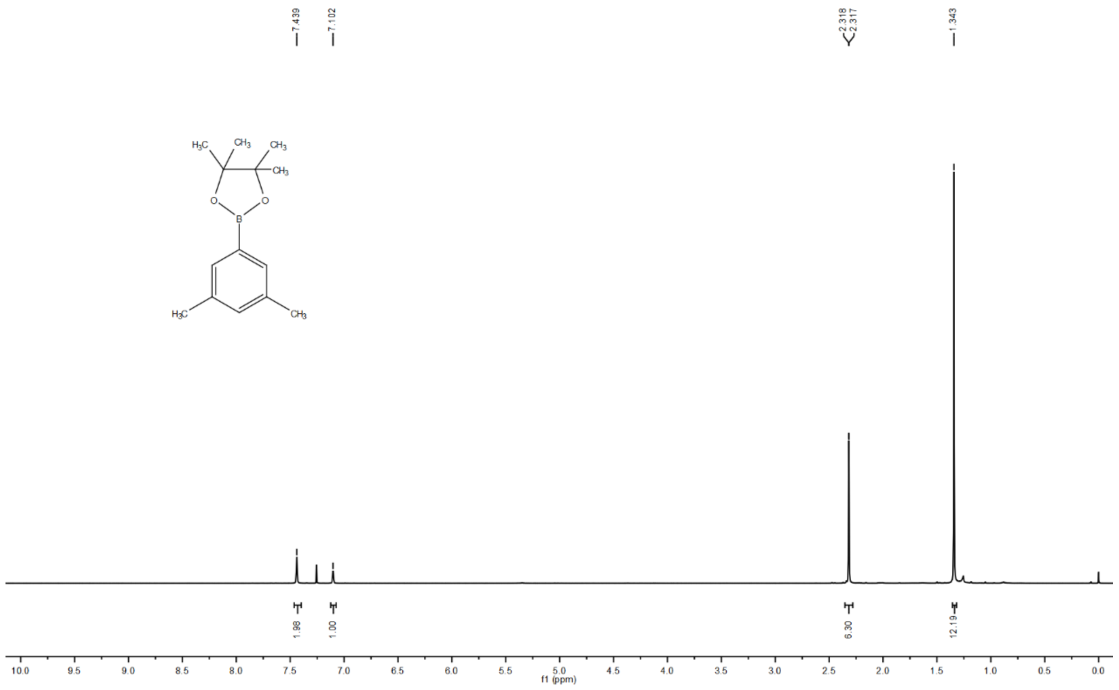

Supplementary Fig. 13.¹H NMR (400 MHz, 298 K, $CDCl_3$) of **2b**

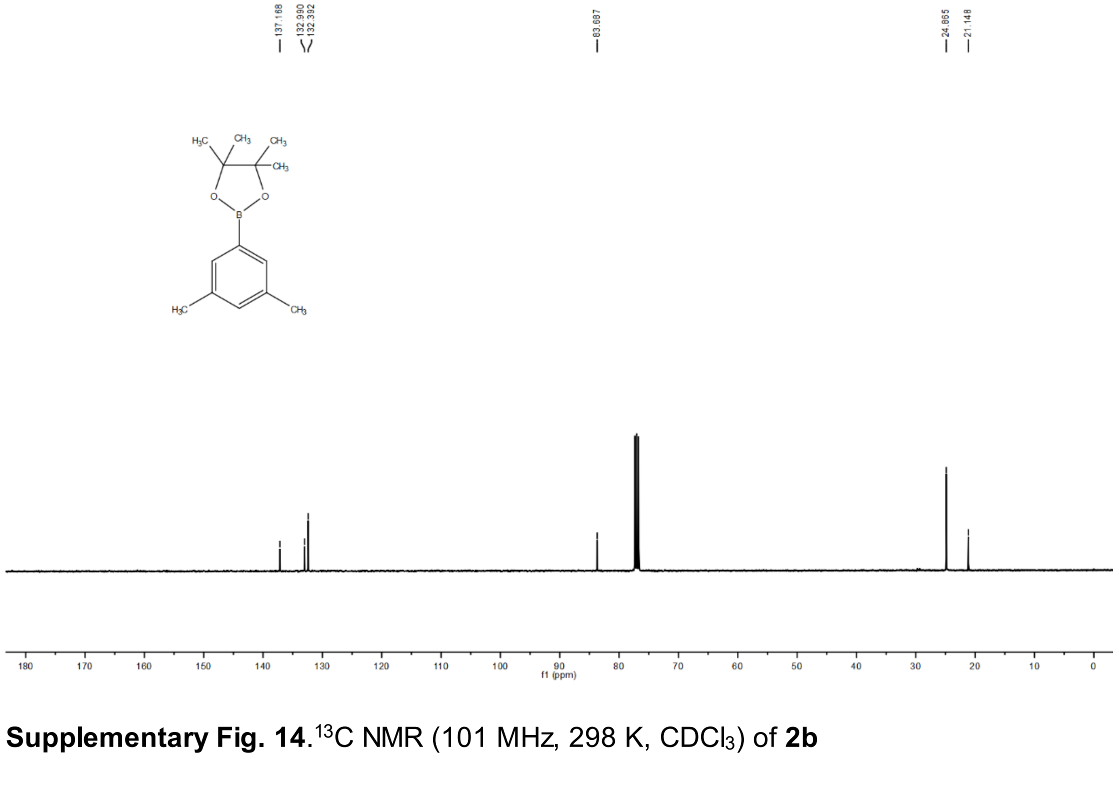

Supplementary Fig. 14.¹³C NMR (101 MHz, 298 K, $CDCl_3$) of **2b**

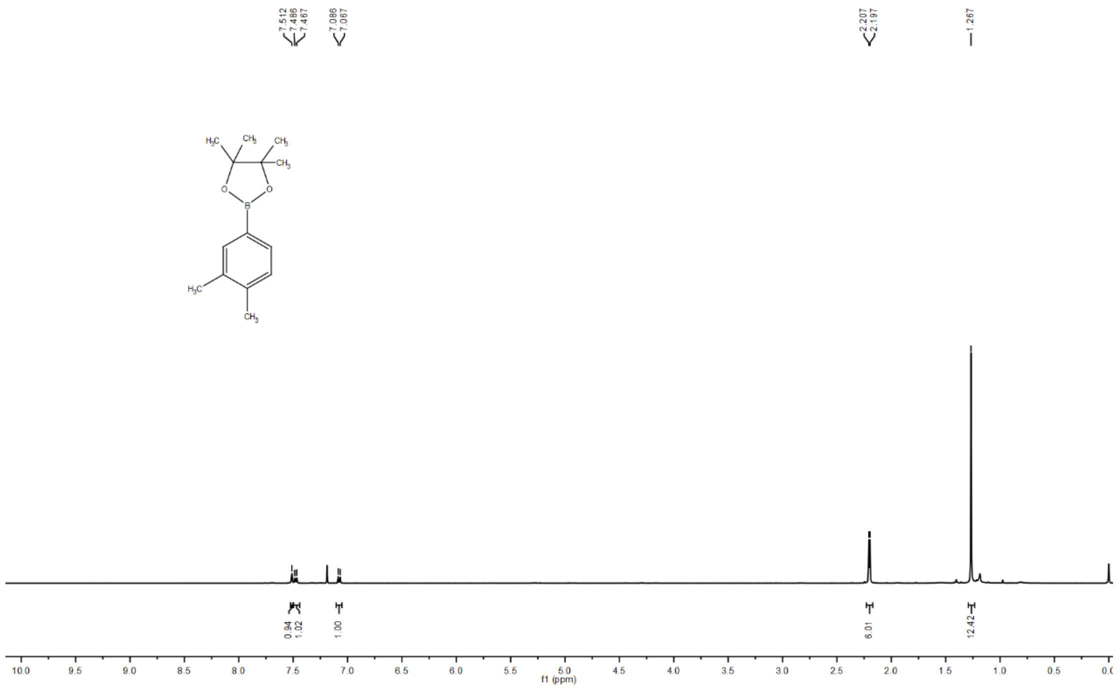

Supplementary Fig. 15.^{1}H NMR (400 MHz, 298 K, $CDCl_3$) of **2c**

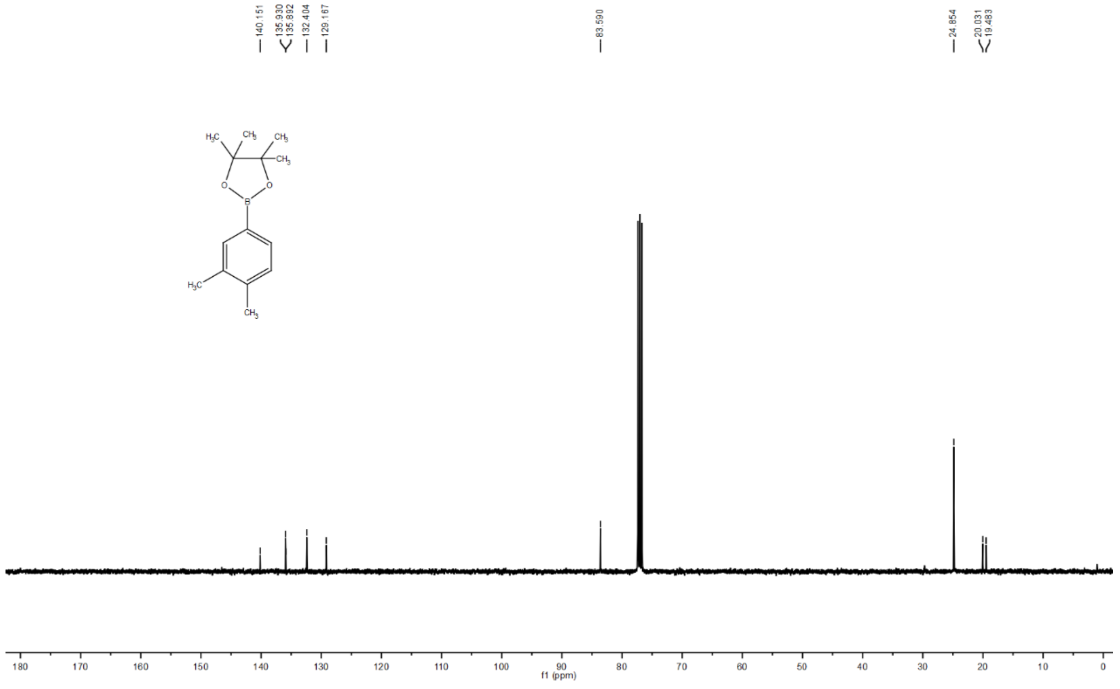

Supplementary Fig. 16.^{13}C NMR (101 MHz, 298 K, $CDCl_3$) of **2c**

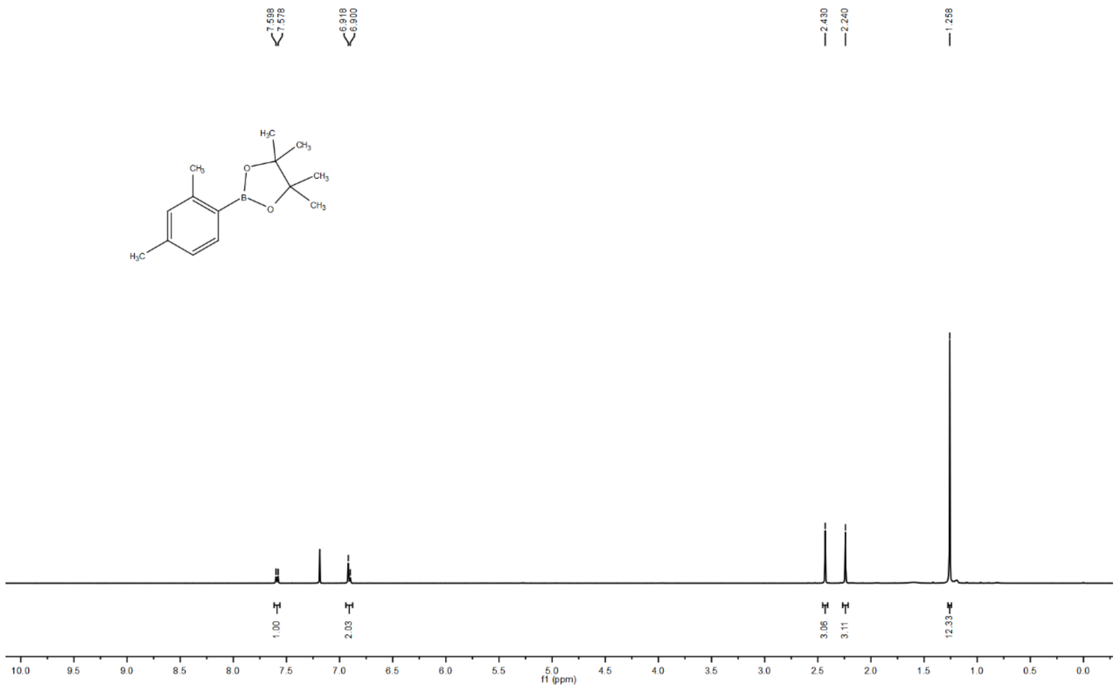

Supplementary Fig. 17.^{1}H NMR (400 MHz, 298 K, $CDCl_3$) of **2d**

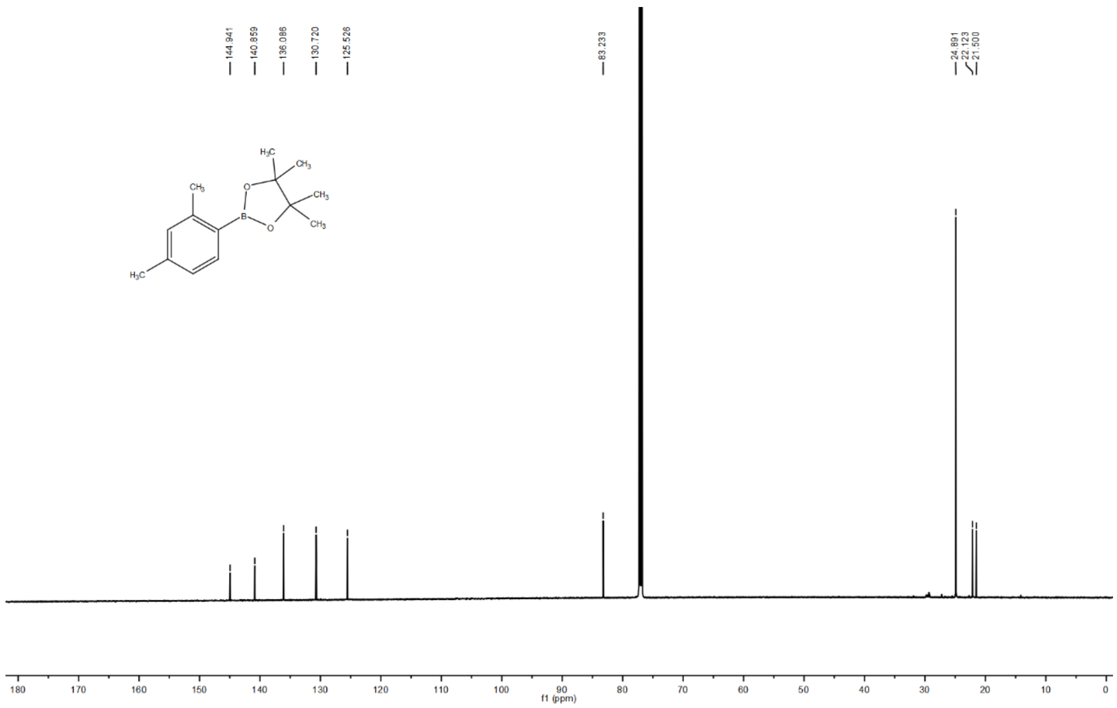

Supplementary Fig. 18.^{13}C NMR (101 MHz, 298 K, $CDCl_3$) of **2d**

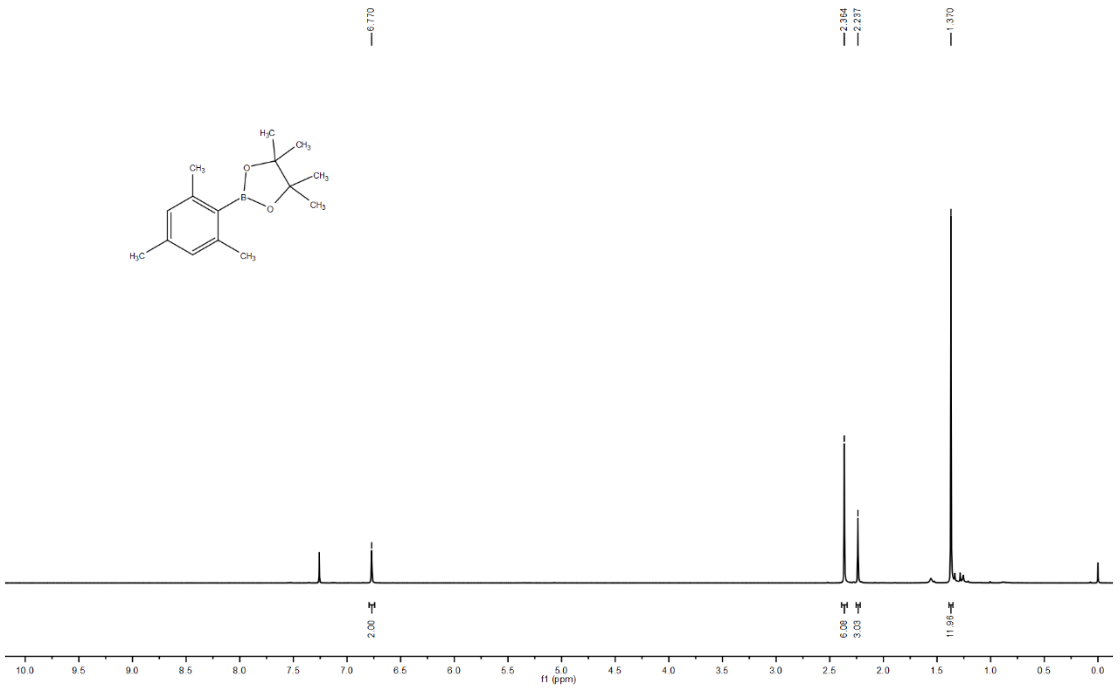

Supplementary Fig. 19.^{1}H NMR (400 MHz, 298 K, $CDCl_3$) of **2e**

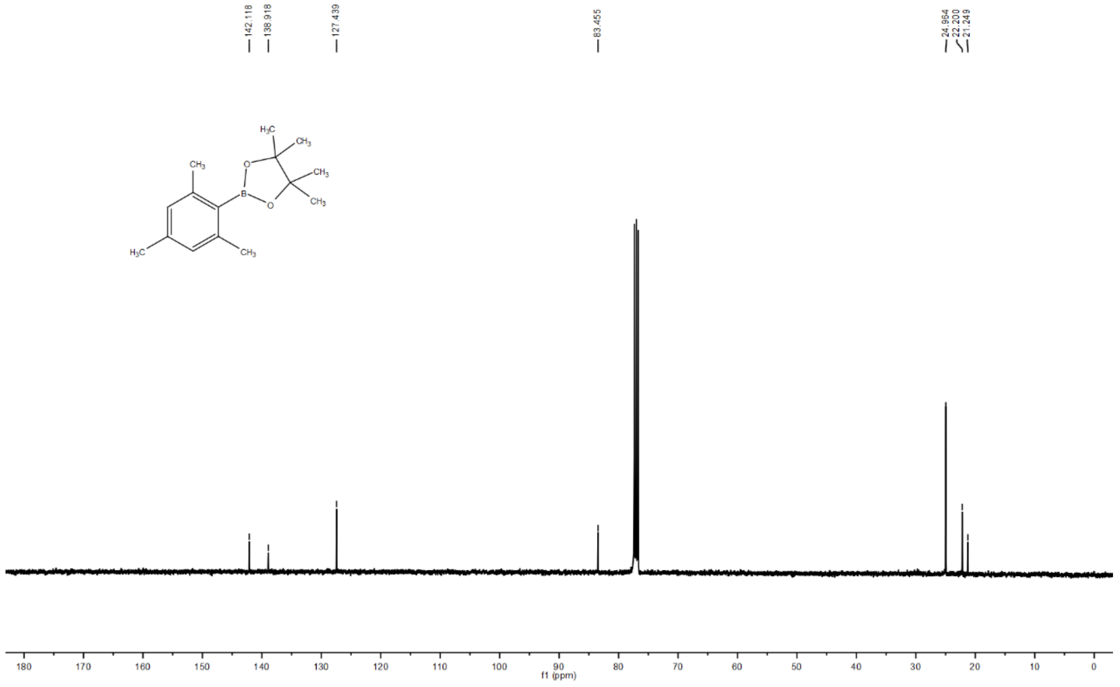

Supplementary Fig. 20.^{13}C NMR (101 MHz, 298 K, $CDCl_3$) of **2e**

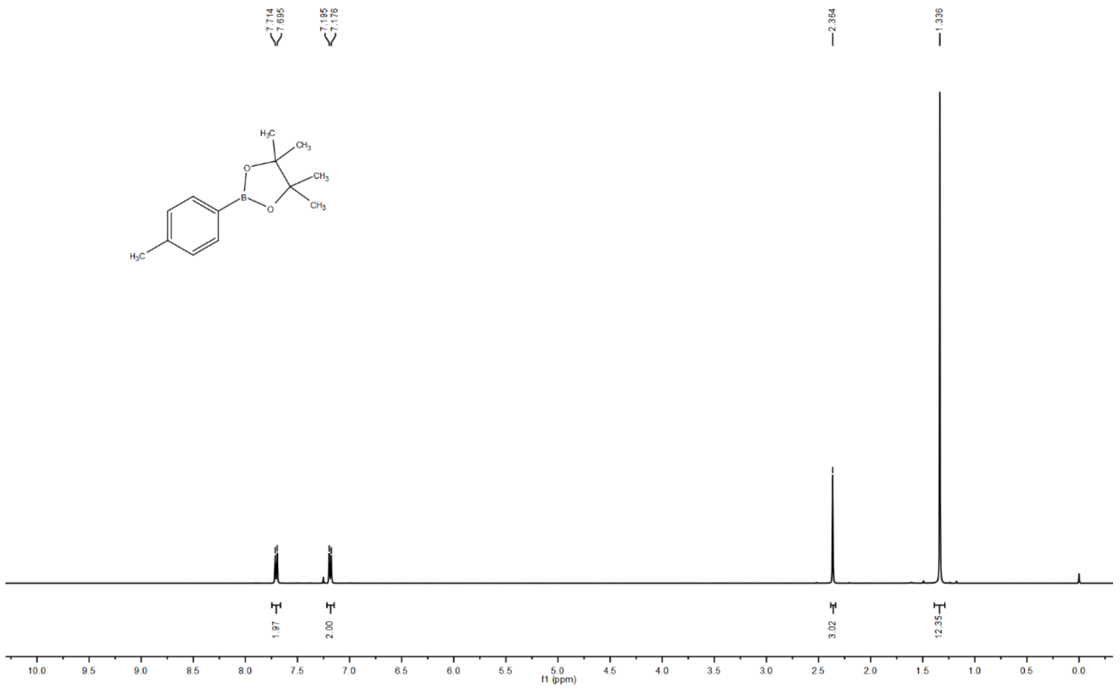

Supplementary Fig. 21.^{1}H NMR (400 MHz, 298 K, $CDCl_3$) of **2f**

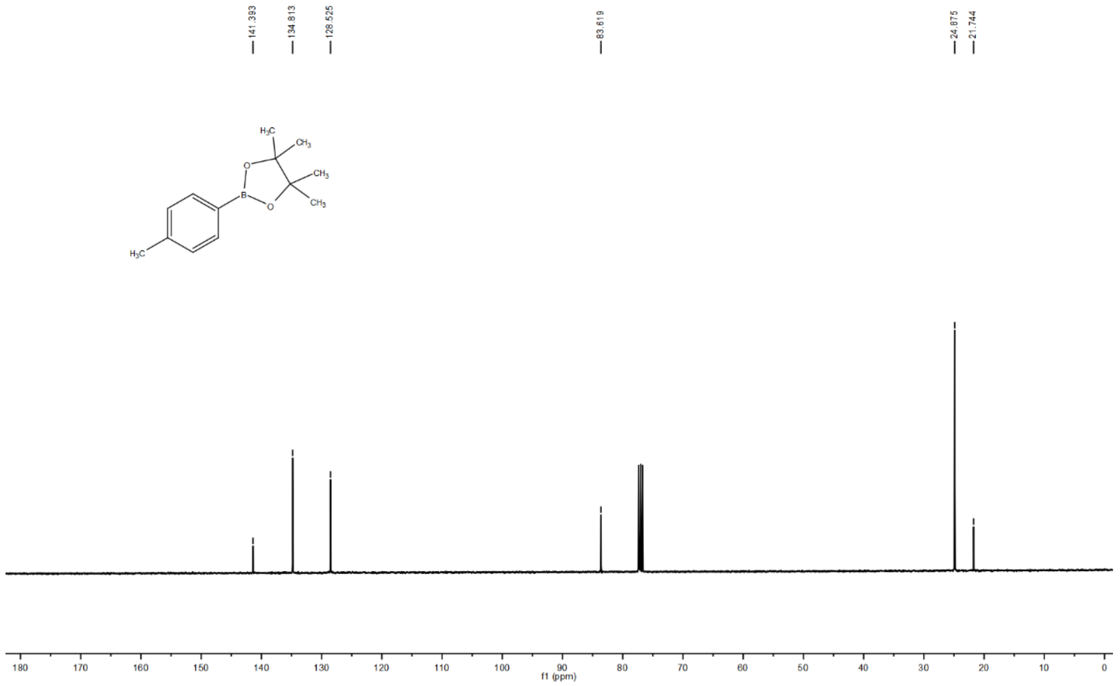

Supplementary Fig. 22.^{13}C NMR (101 MHz, 298 K, $CDCl_3$) of **2f**

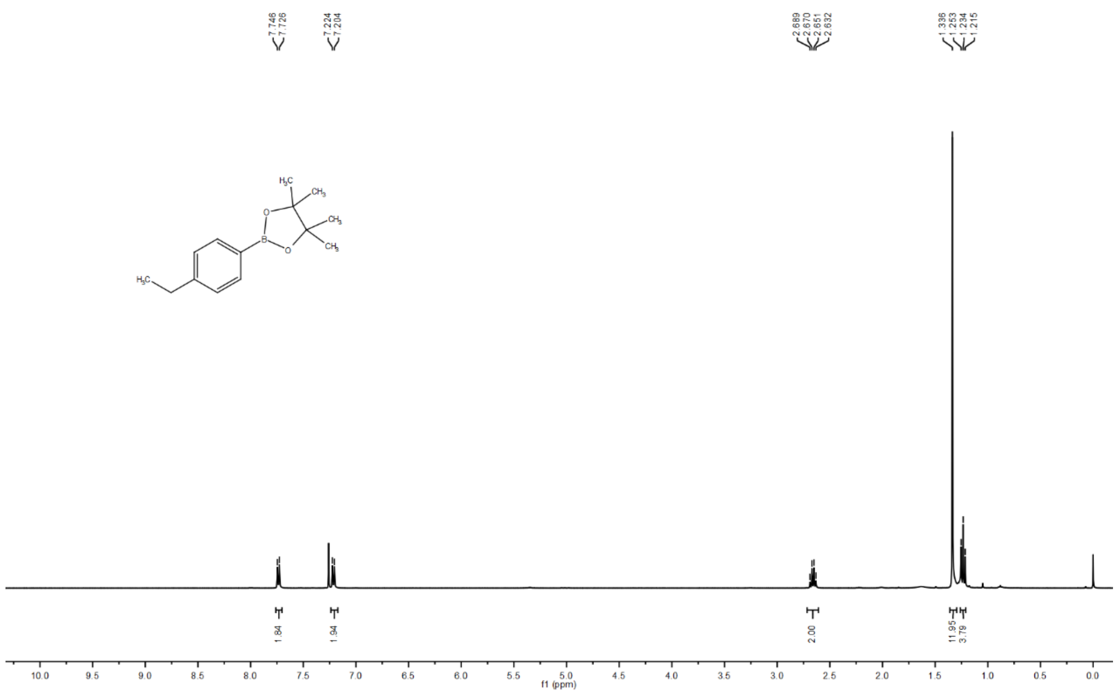

Supplementary Fig. 23.^{1}H NMR (400 MHz, 298 K, $CDCl_3$) of **2g**

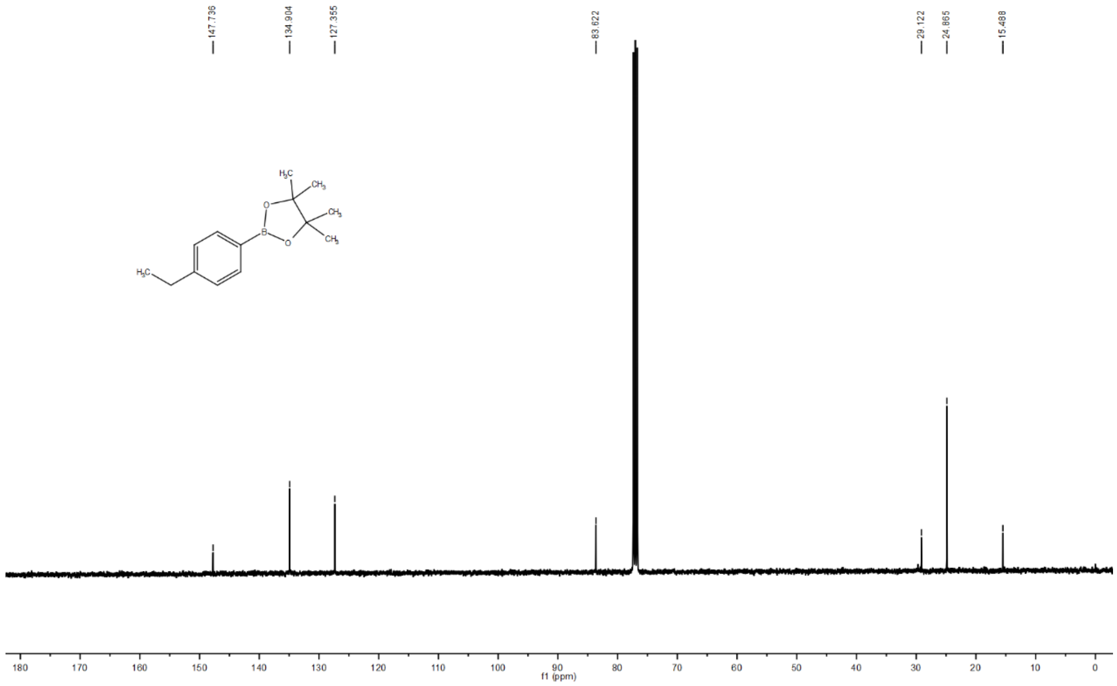

Supplementary Fig. 24.^{13}C NMR (101 MHz, 298 K, $CDCl_3$) of **2g**

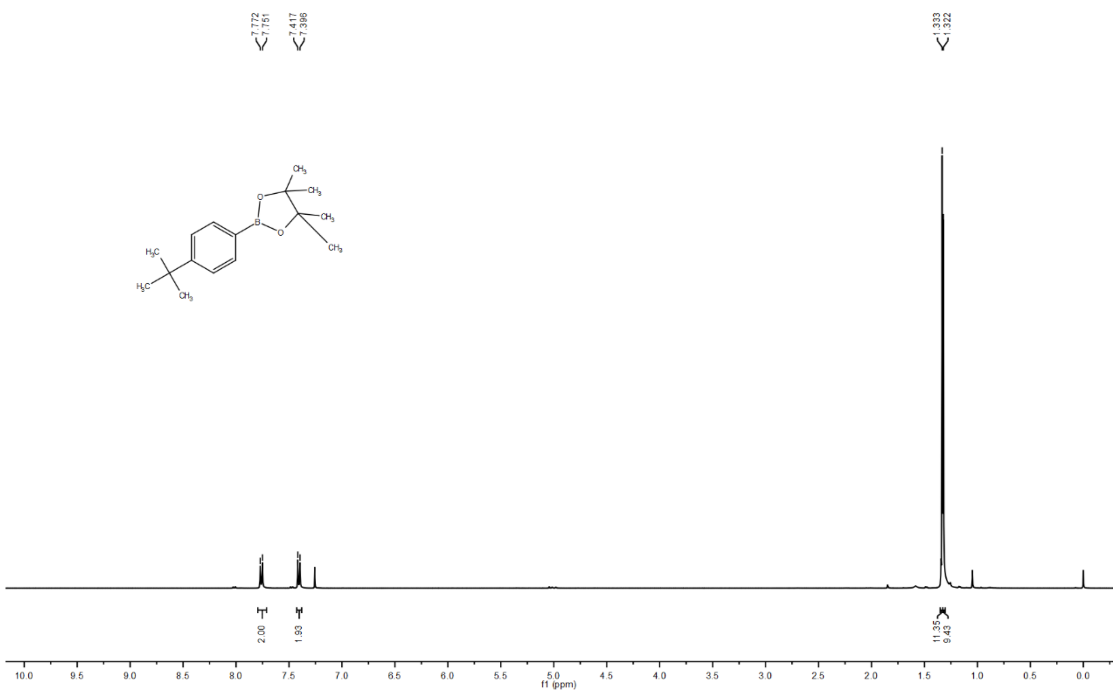

Supplementary Fig. 25.^{1}H NMR (400 MHz, 298 K, $CDCl_3$) of **2h**

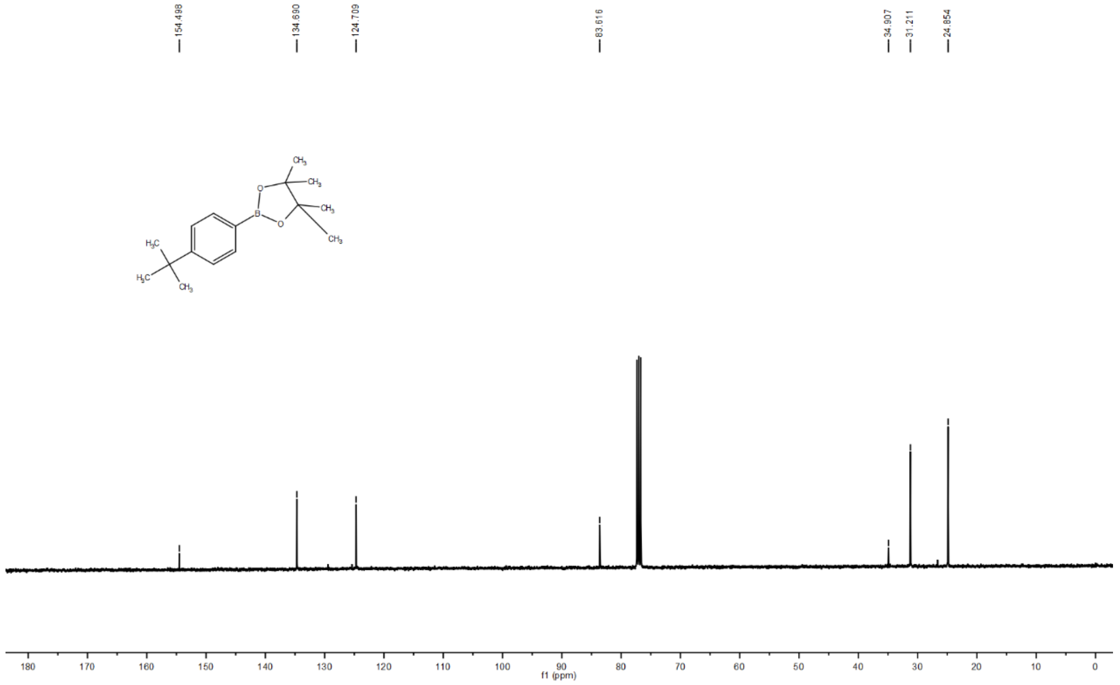

Supplementary Fig. 26.^{13}C NMR (101 MHz, 298 K, $CDCl_3$) of **2h**

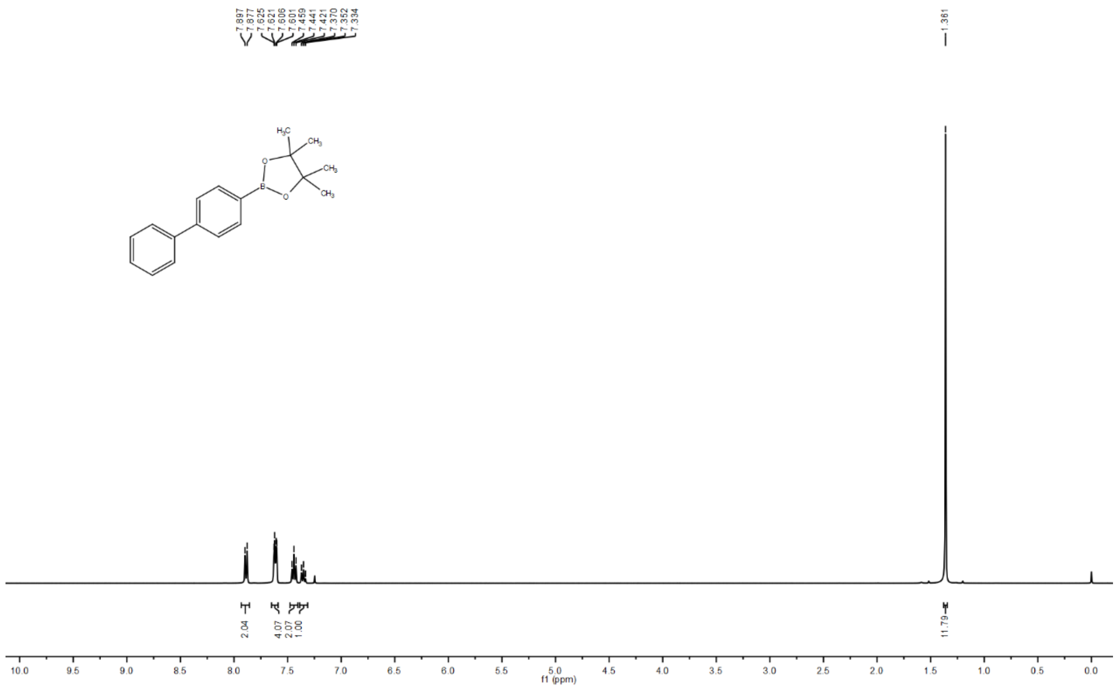

Supplementary Fig. 27.^{1}H NMR (400 MHz, 298 K, $CDCl_3$) of **2i**

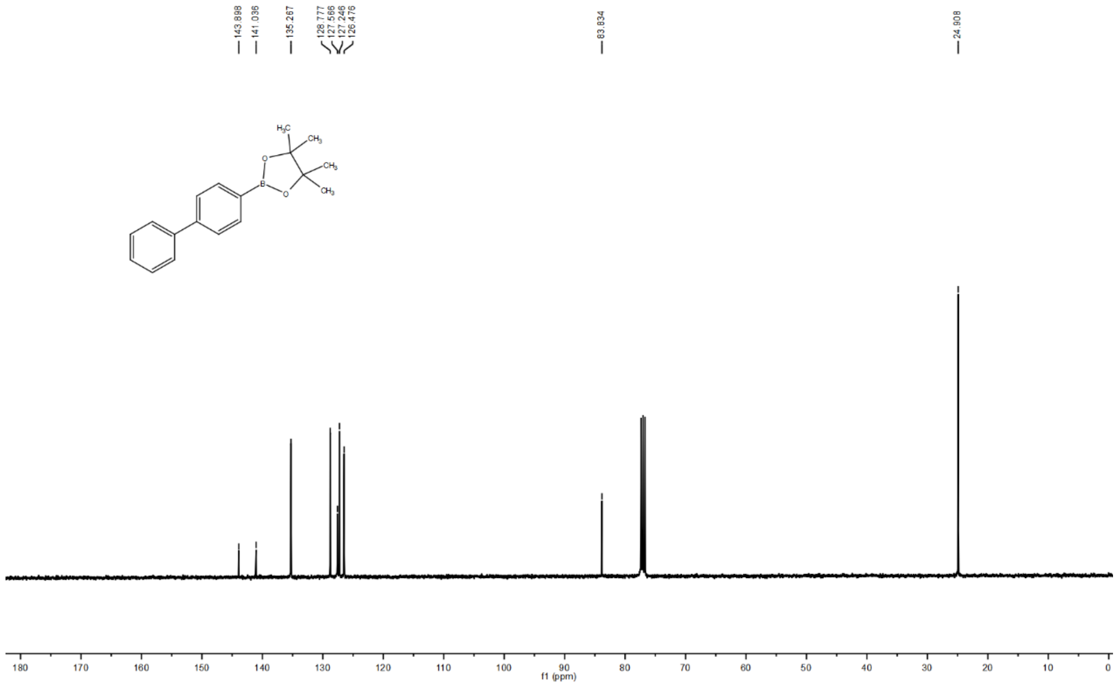

Supplementary Fig. 28.^{13}C NMR (101 MHz, 298 K, $CDCl_3$) of **2i**

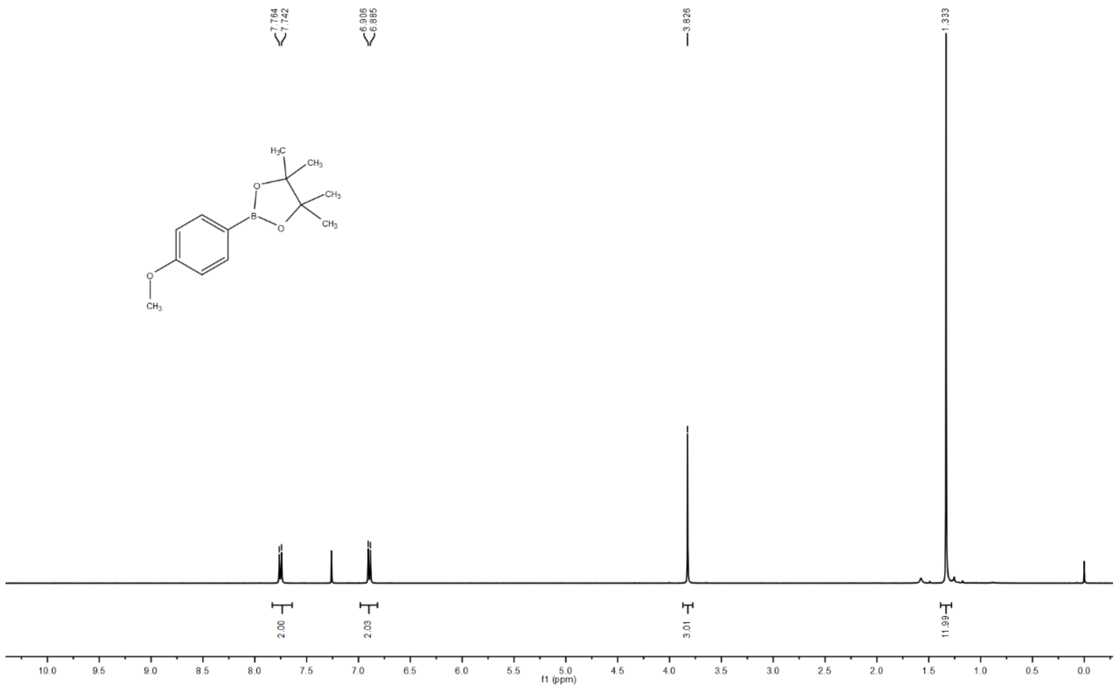

Supplementary Fig. 29. ^{1}H NMR (400 MHz, 298 K, $CDCl_3$) of **2j**

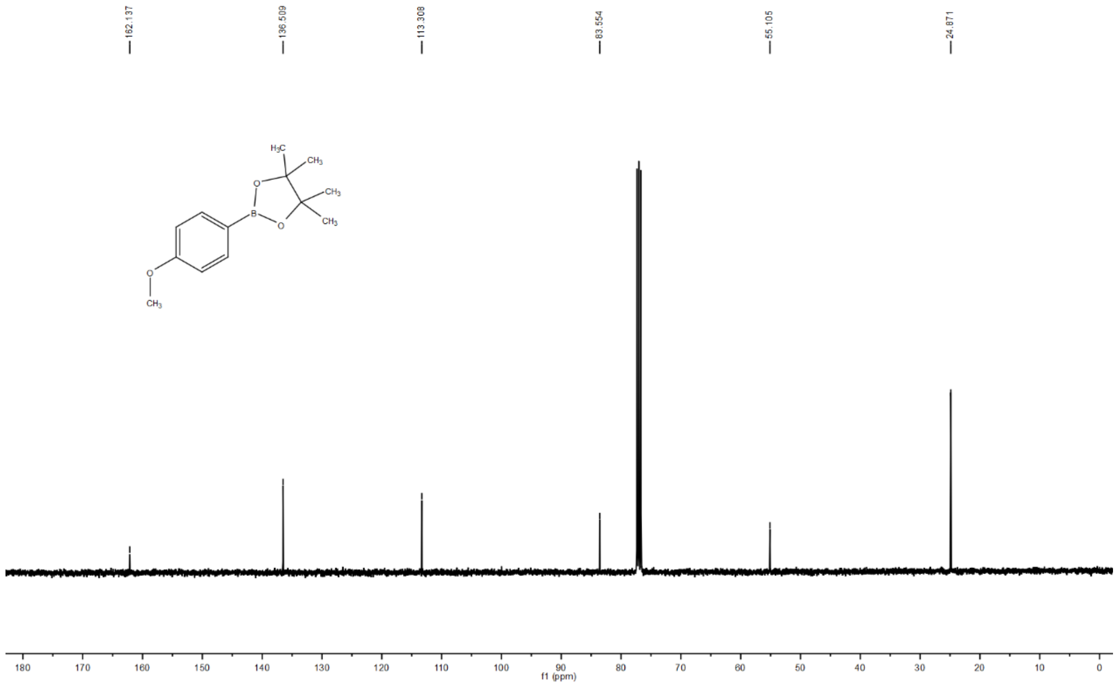

Supplementary Fig. 30. ^{13}C NMR (101 MHz, 298 K, $CDCl_3$) of **2j**

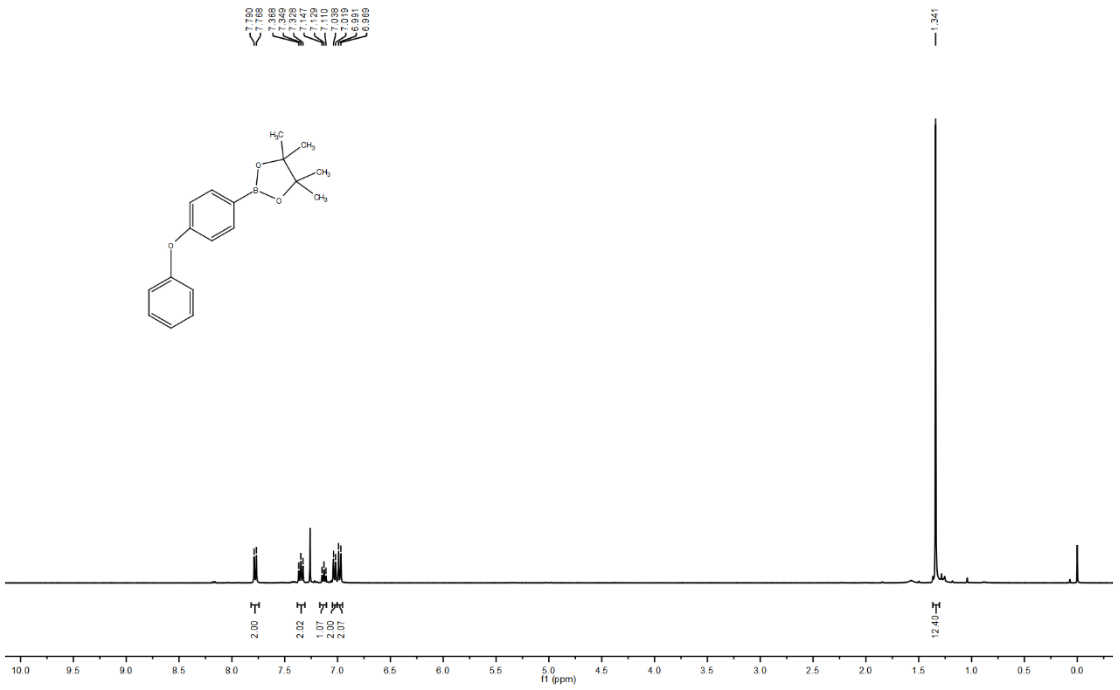

Supplementary Fig. 31.^{1}H NMR (400 MHz, 298 K, $CDCl_3$) of **2k**

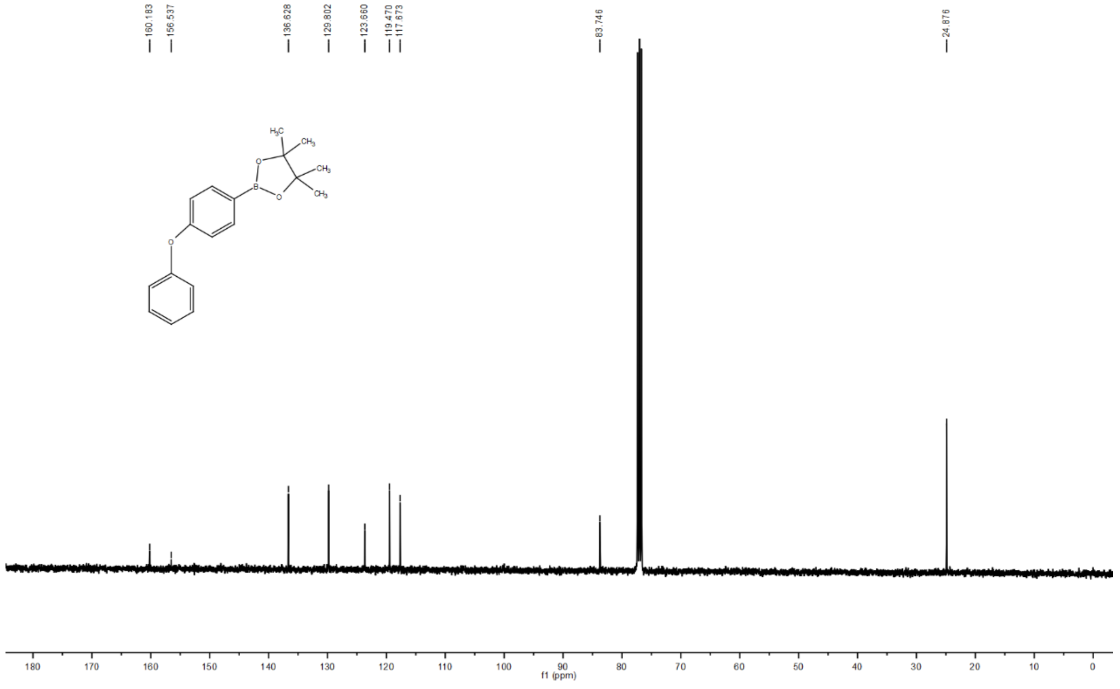

Supplementary Fig. 32.^{13}C NMR (101 MHz, 298 K, $CDCl_3$) of **2k**

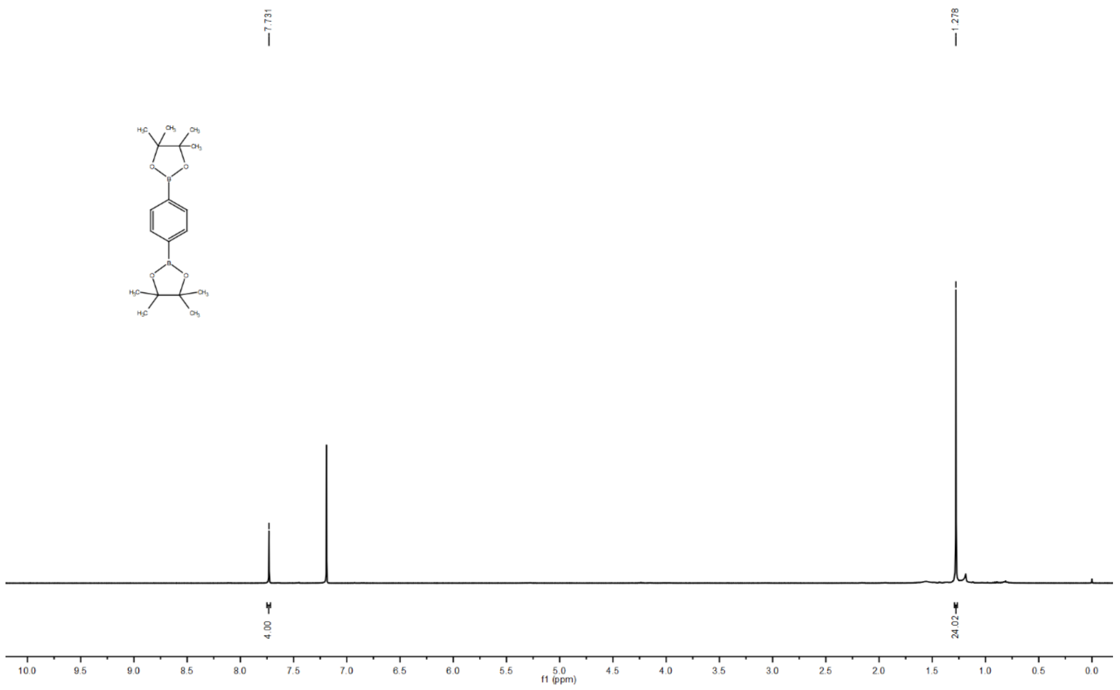

Supplementary Fig. 33.^{1}H NMR (400 MHz, 298 K, $CDCl_3$) of **2l**

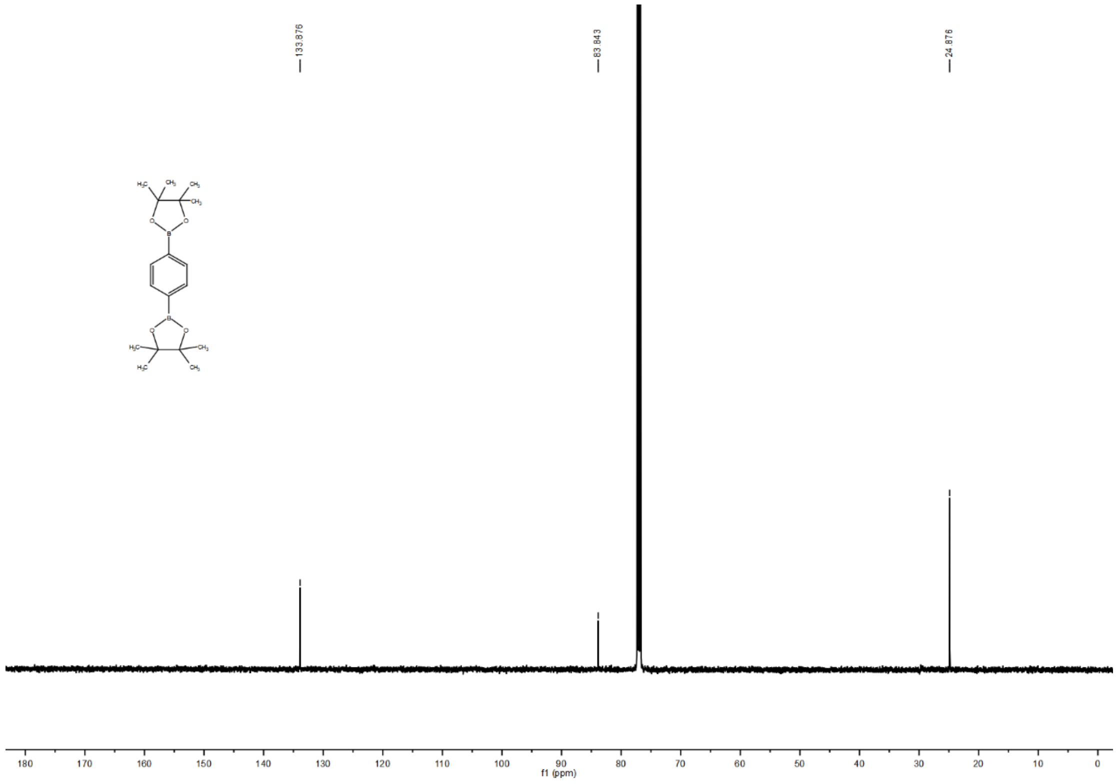

Supplementary Fig. 34.^{13}C NMR (101 MHz, 298 K, $CDCl_3$) of **2l**

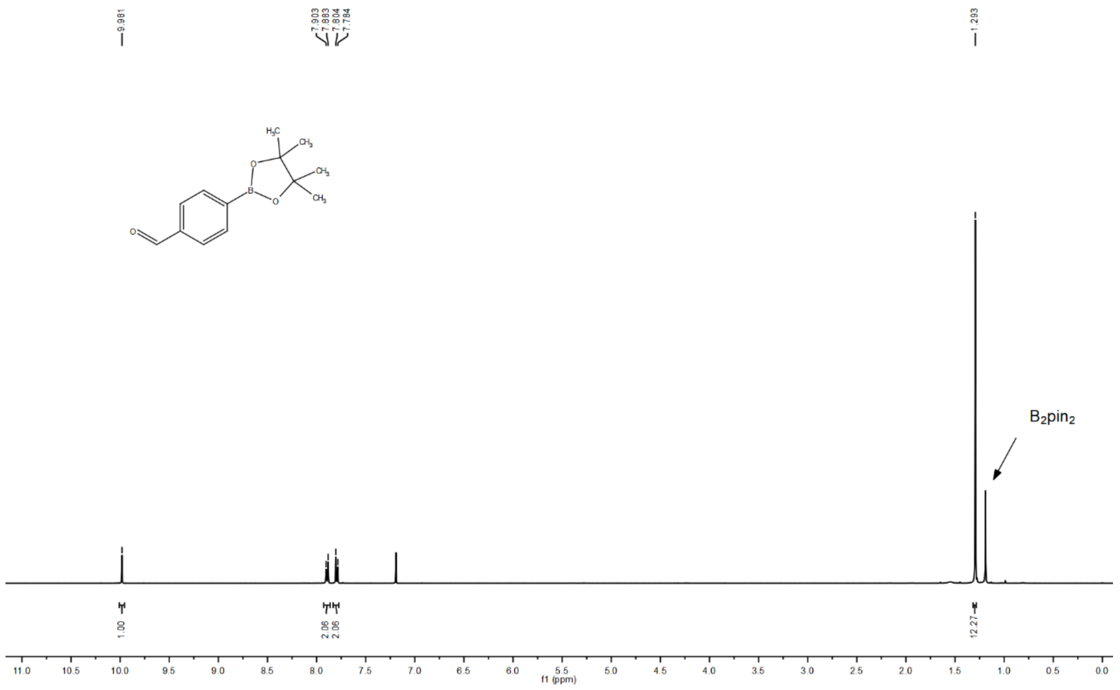

Supplementary Fig. 35.^{1}H NMR (400 MHz, 298 K, $CDCl_3$) of **2m**

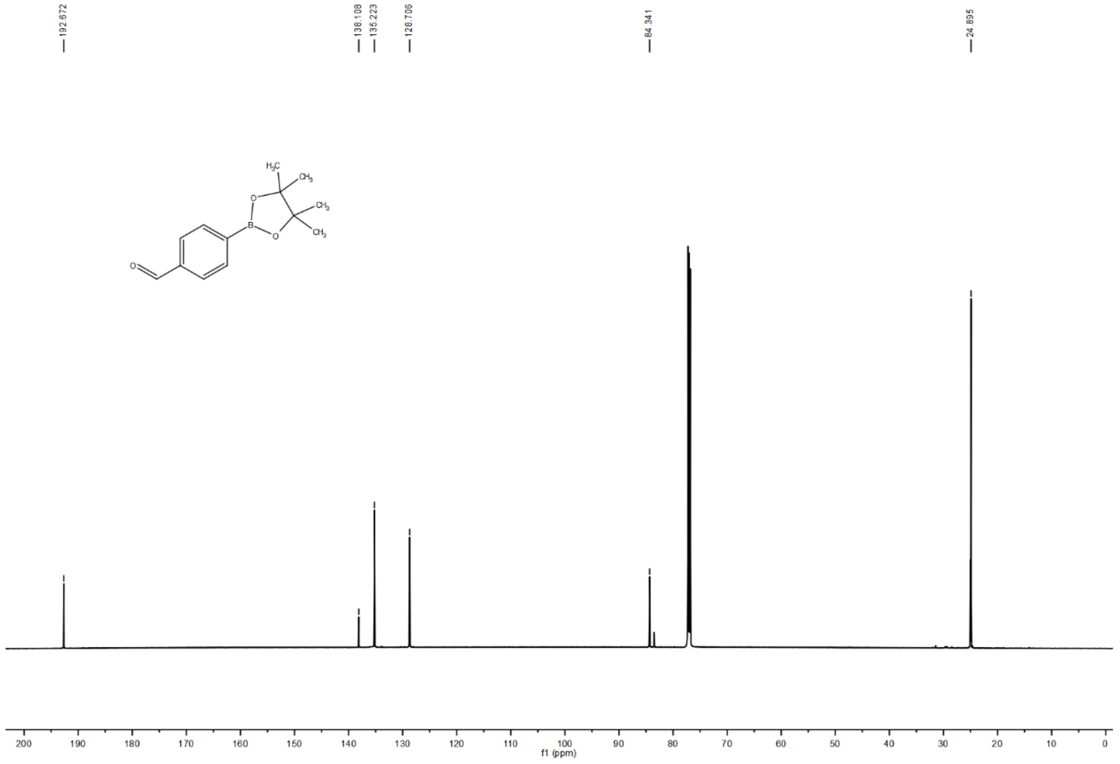

Supplementary Fig. 36.^{13}C NMR (101 MHz, 298 K, $CDCl_3$) of **2m**

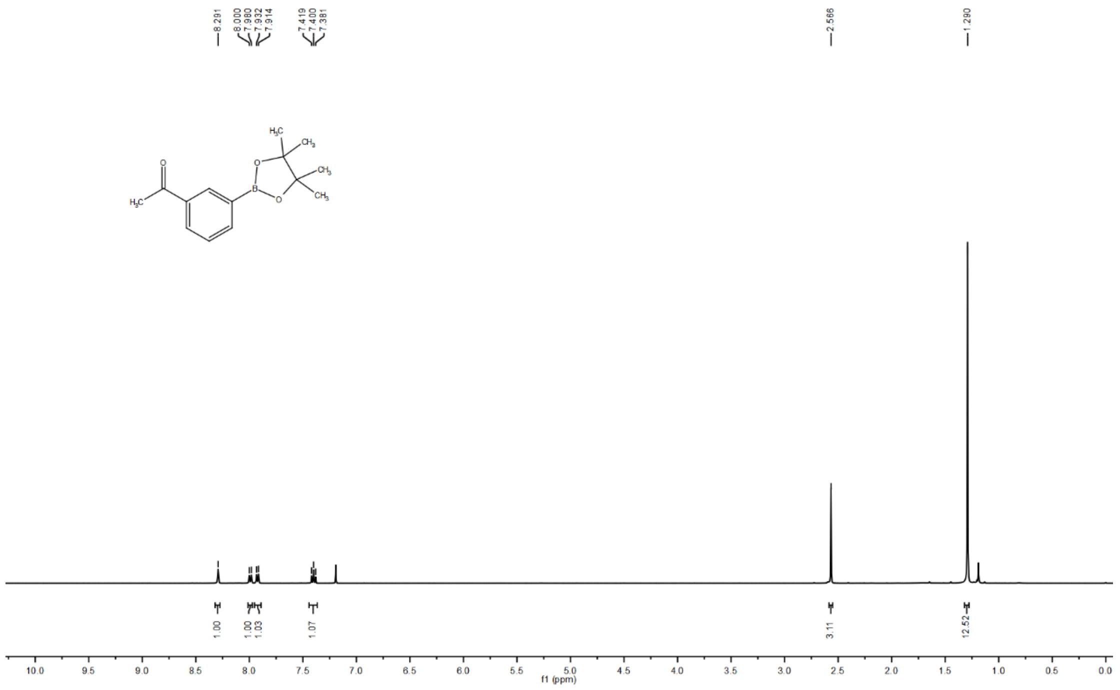

Supplementary Fig. 37.^{1}H NMR (400 MHz, 298 K, $CDCl_3$) of **2n**

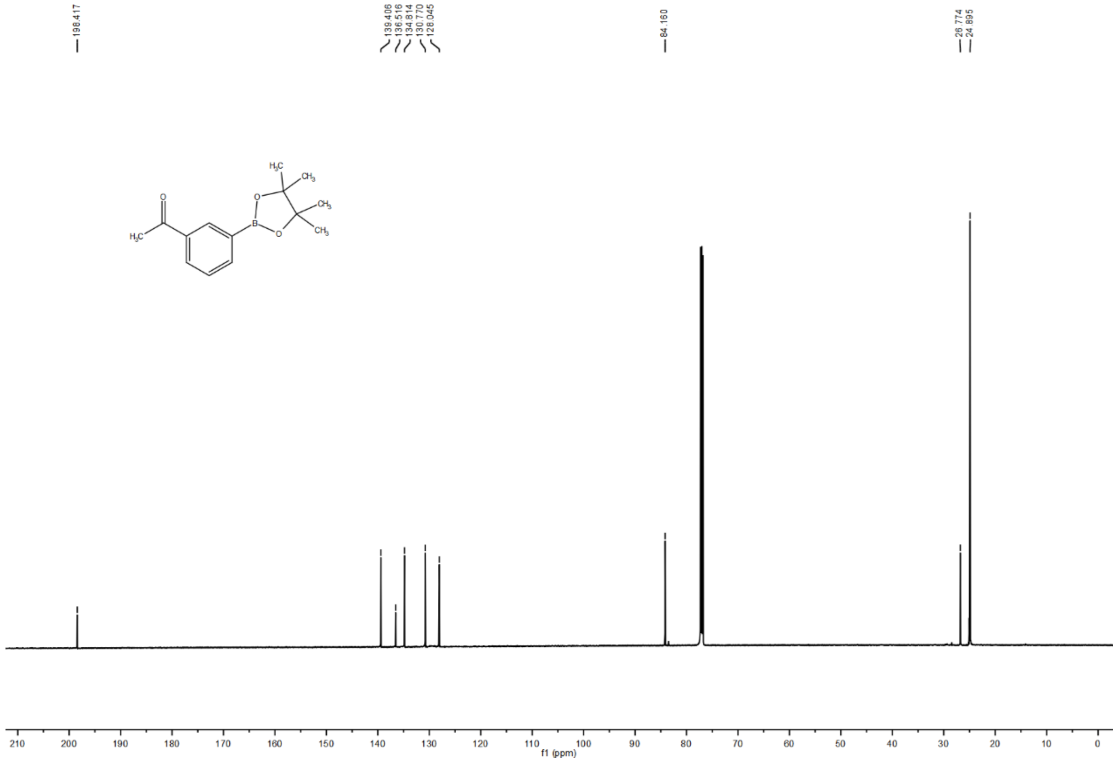

Supplementary Fig. 38.^{13}C NMR (101 MHz, 298 K, $CDCl_3$) of **2n**

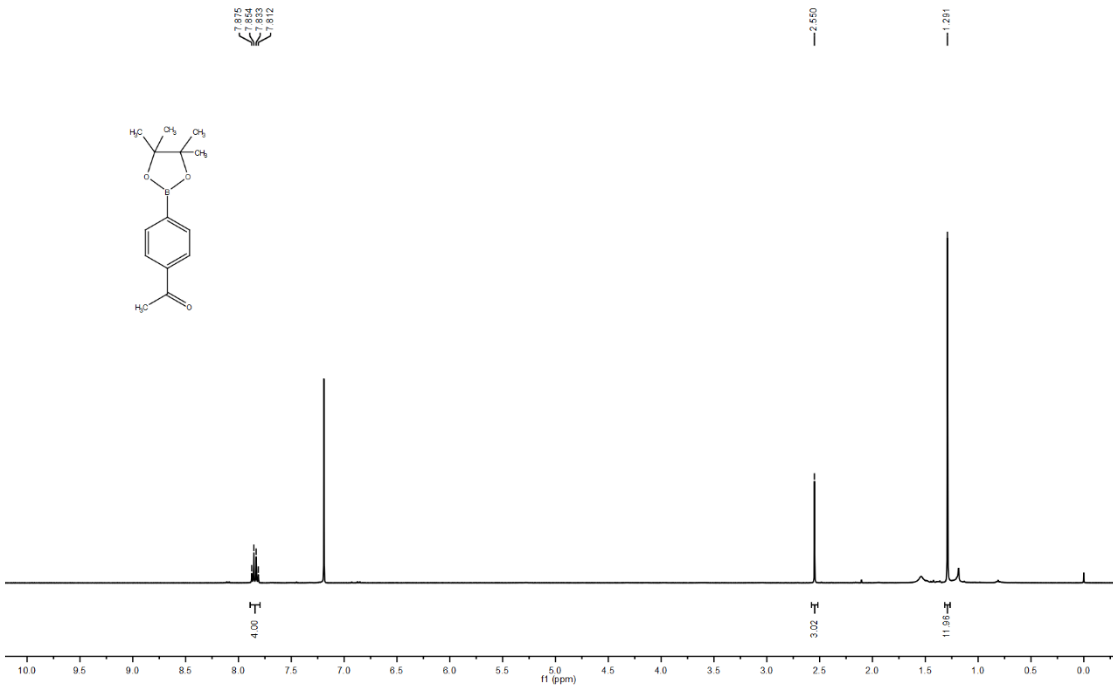

Supplementary Fig. 39. ^{1}H NMR (400 MHz, 298 K, $CDCl_3$) of **2o**

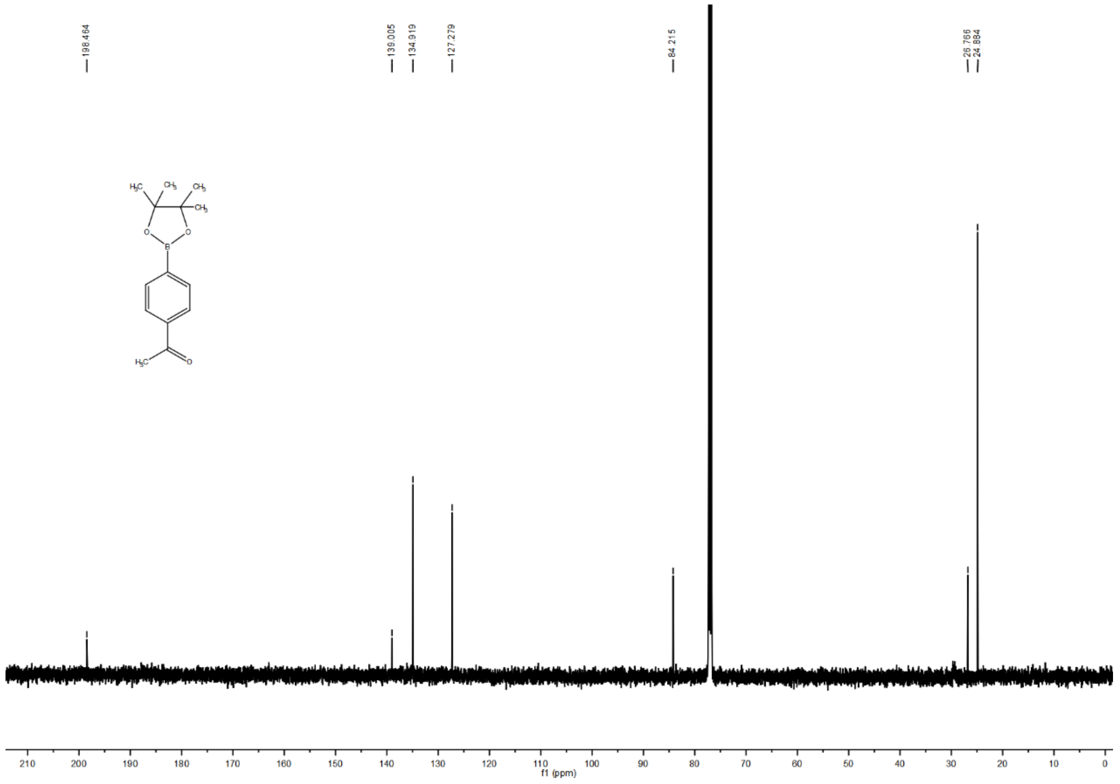

Supplementary Fig. 40. ^{13}C NMR (101 MHz, 298 K, $CDCl_3$) of **2o**

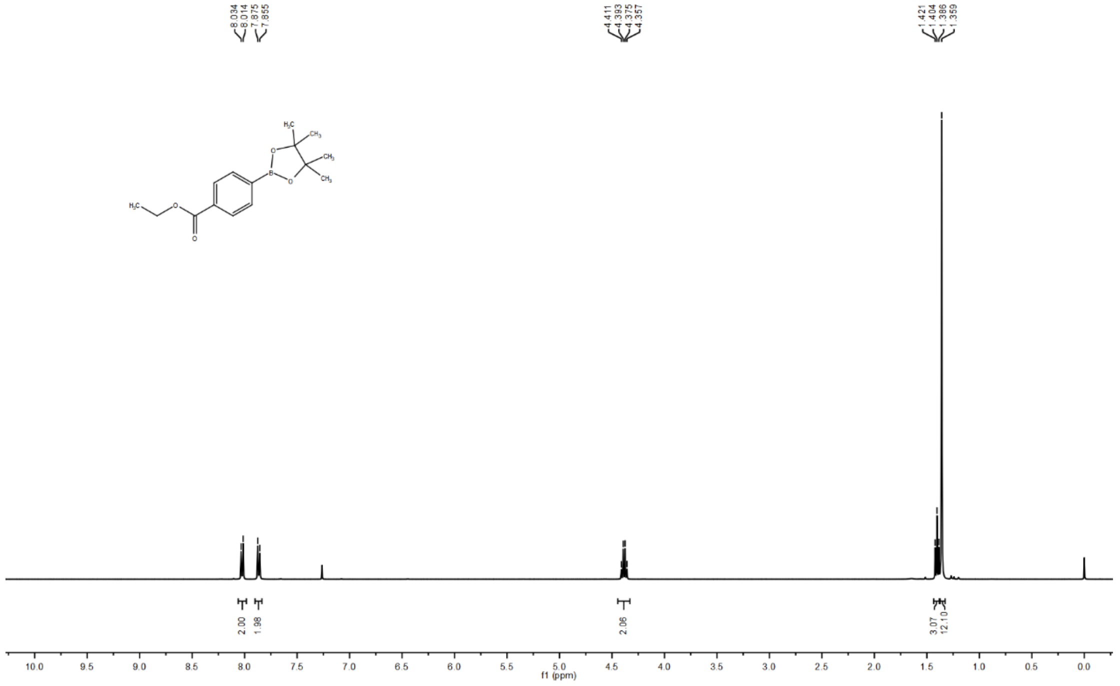

Supplementary Fig. 41.^{1}H NMR (400 MHz, 298 K, $CDCl_3$) of **2p**

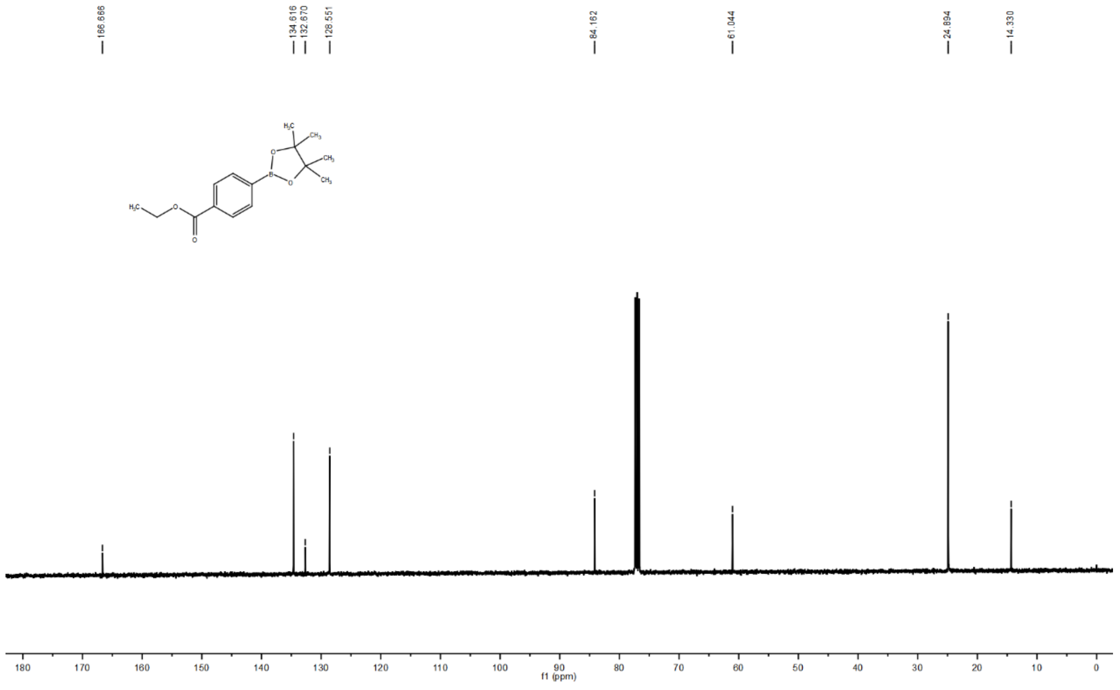

Supplementary Fig. 42.^{13}C NMR (101 MHz, 298 K, $CDCl_3$) of **2p**

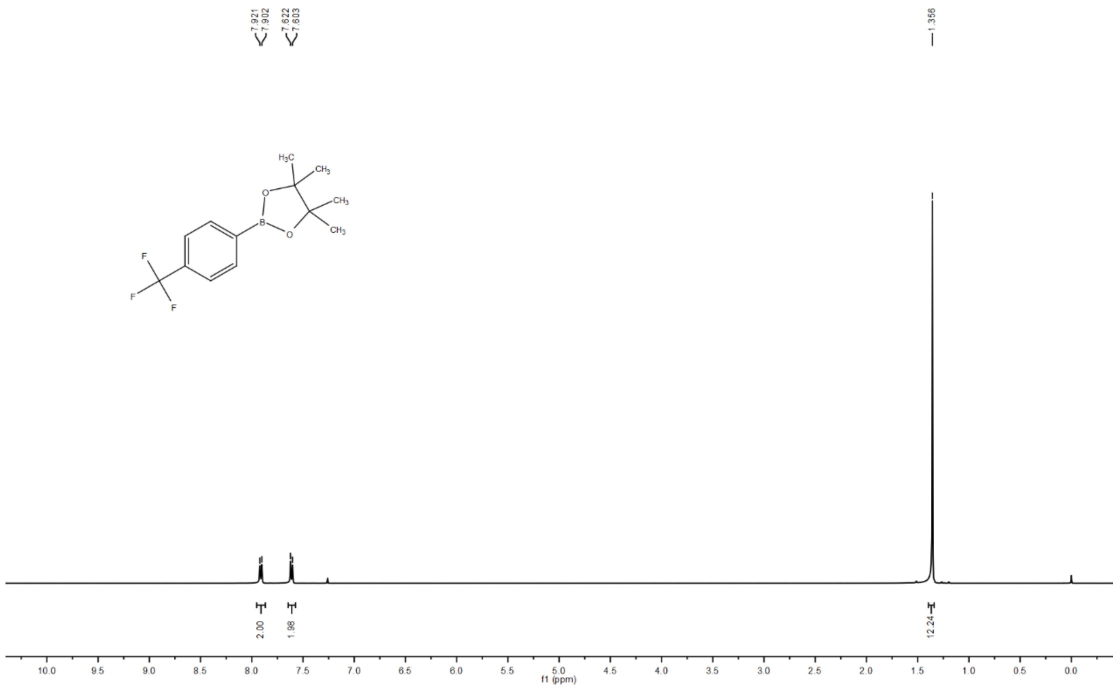

Supplementary Fig. 43.^{1}H NMR (400 MHz, 298 K, $CDCl_3$) of **2q**

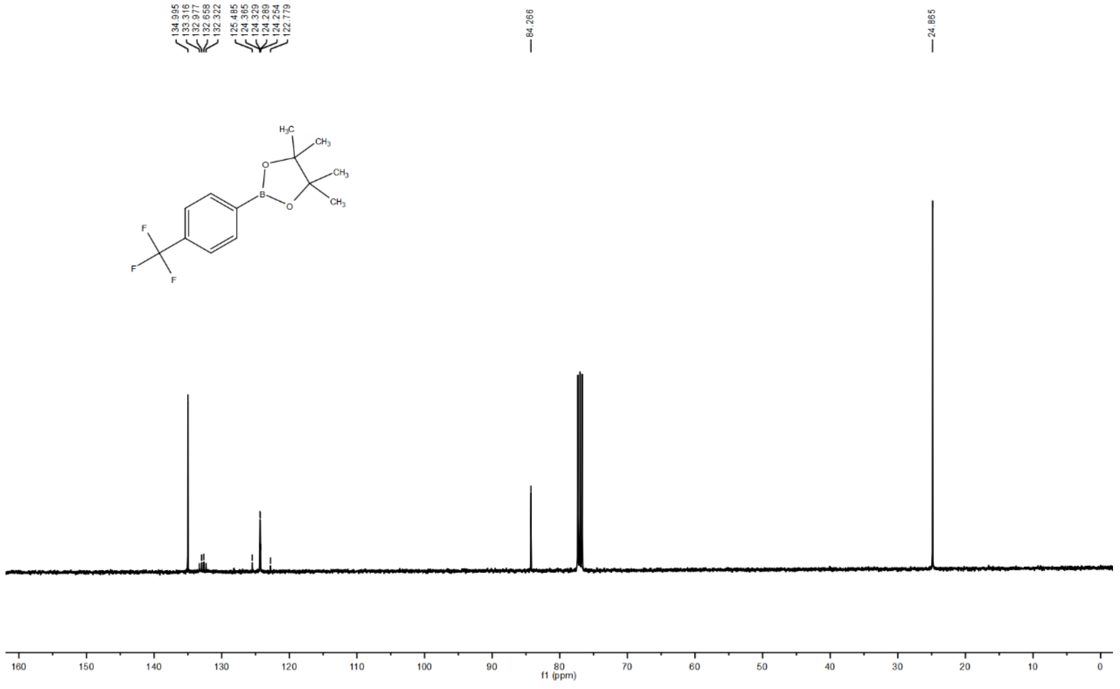

Supplementary Fig. 44.^{13}C NMR (101 MHz, 298 K, $CDCl_3$) of **2q**

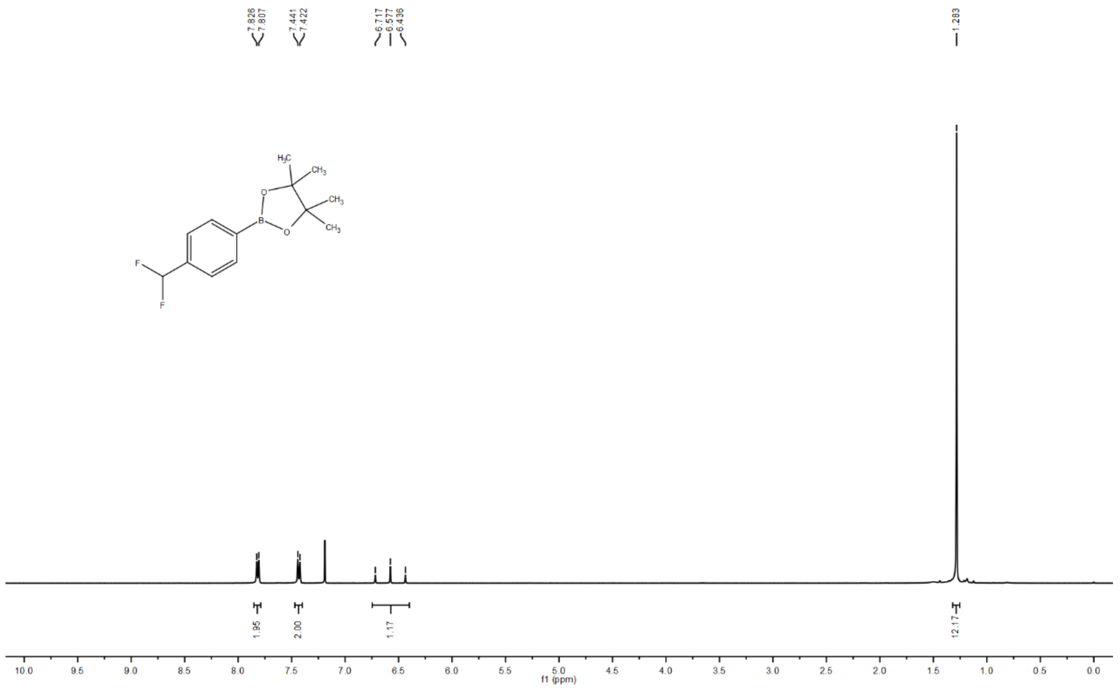

Supplementary Fig. 45.^{1}H NMR (400 MHz, 298 K, $CDCl_3$) of **2r**

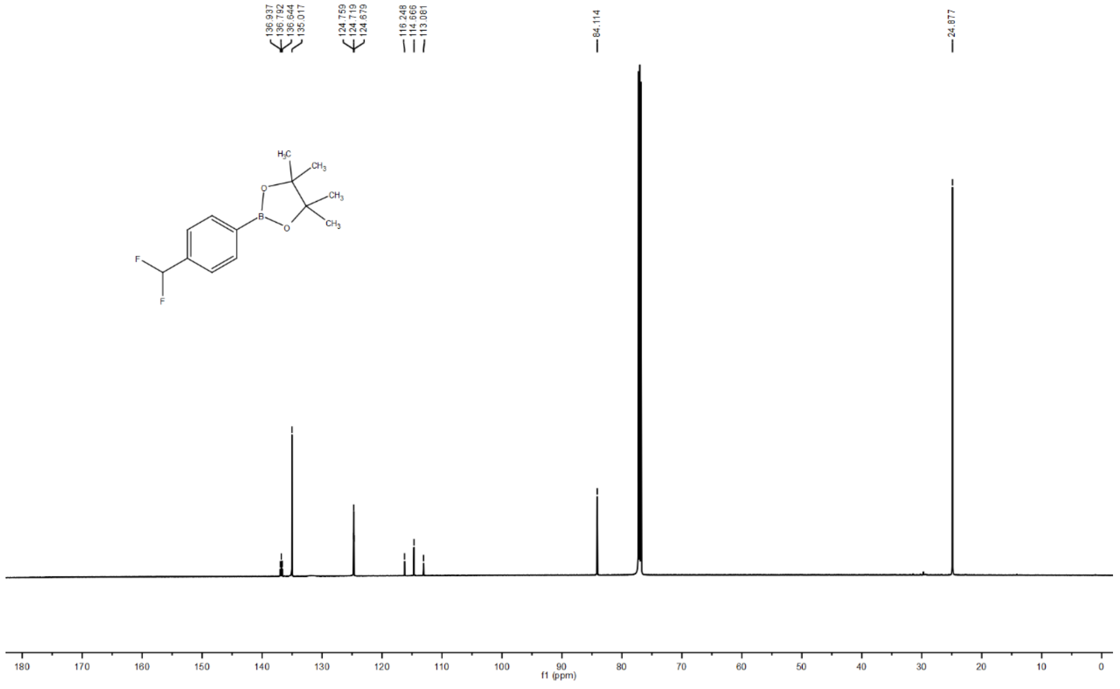

Supplementary Fig. 46.^{13}C NMR (101 MHz, 298 K, $CDCl_3$) of **2r**

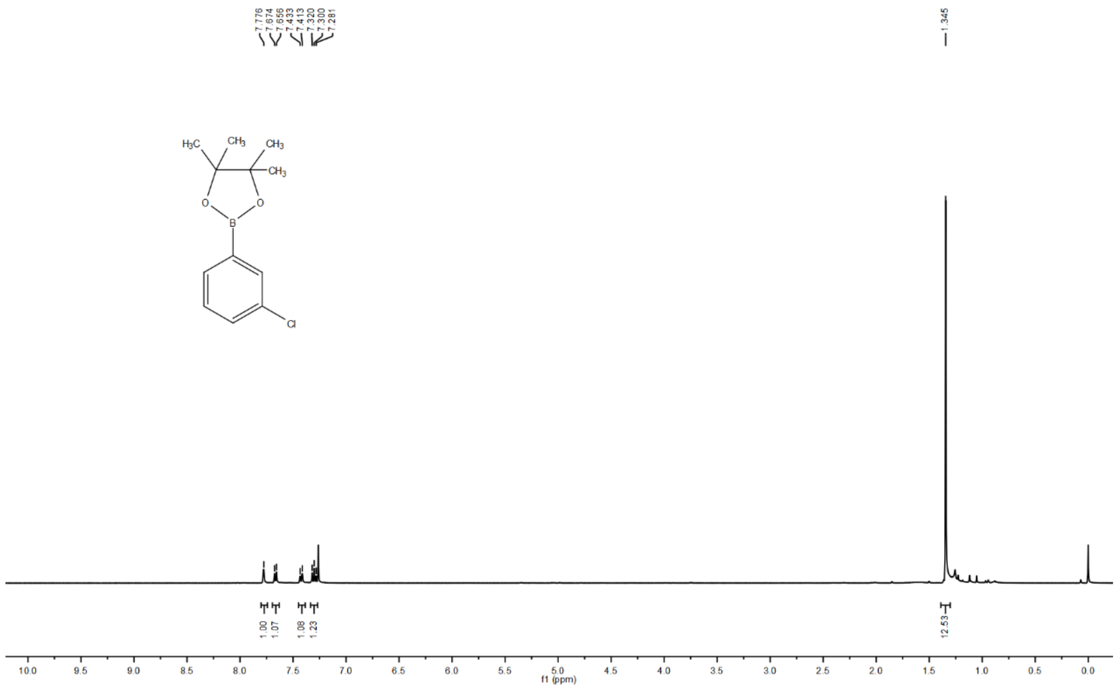

Supplementary Fig. 47.^{1}H NMR (400 MHz, 298 K, $CDCl_3$) of **2s**

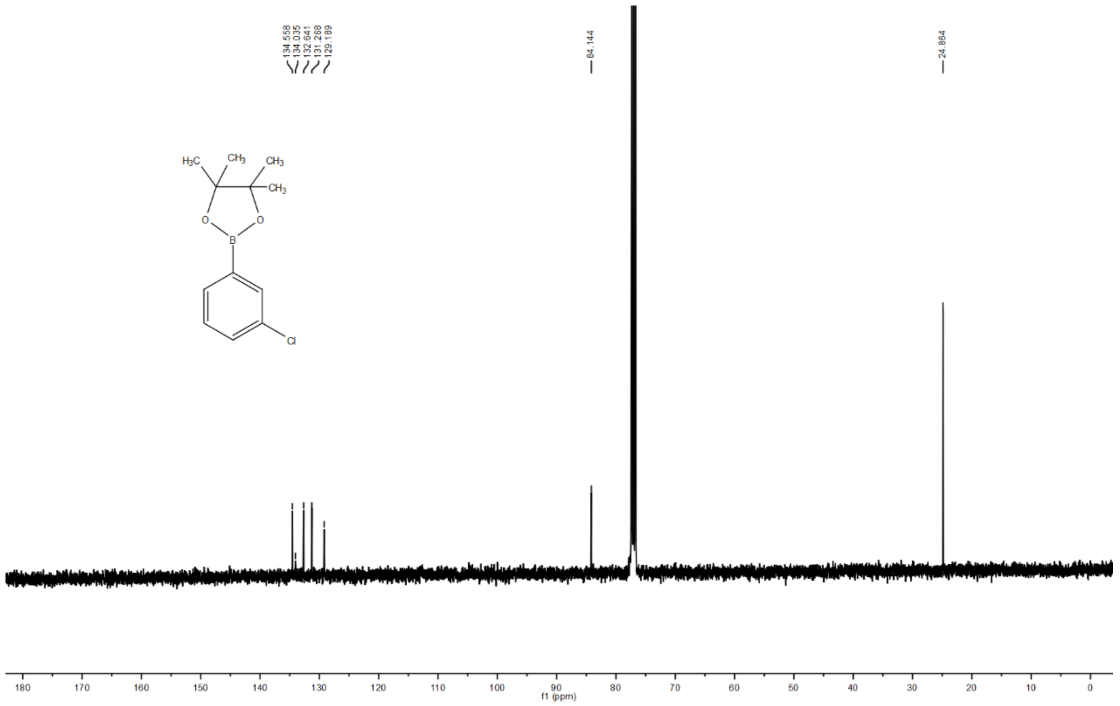

Supplementary Fig. 48.^{13}C NMR (101 MHz, 298 K, $CDCl_3$) of **2s**

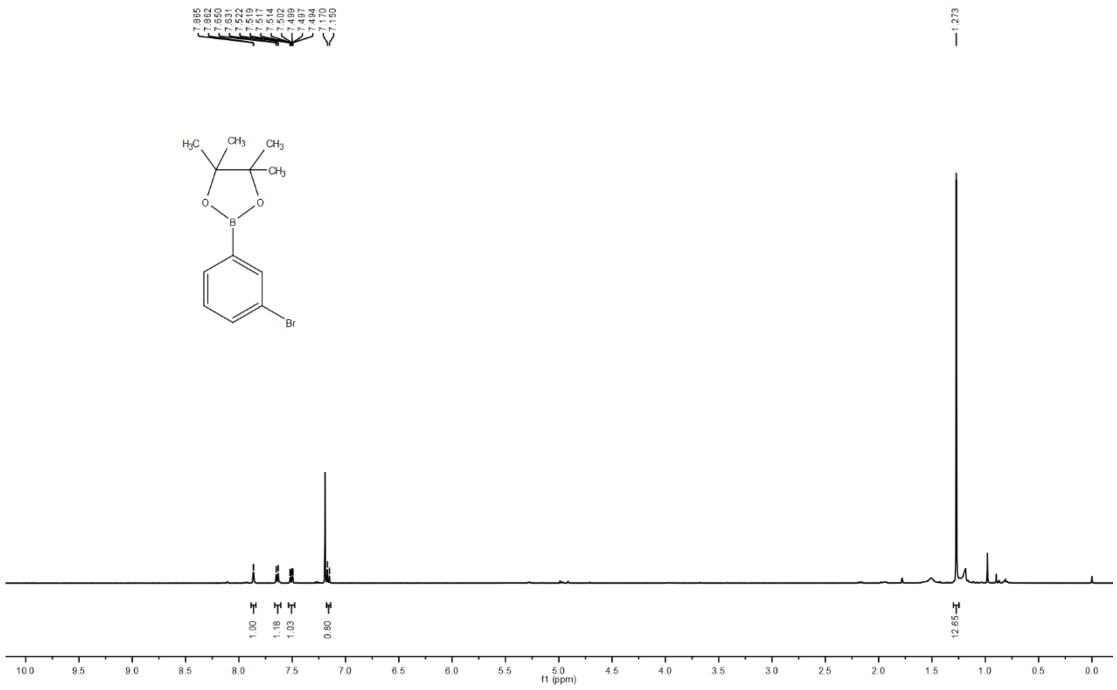

Supplementary Fig. 49.^{1}H NMR (400 MHz, 298 K, $CDCl_3$) of **2t**

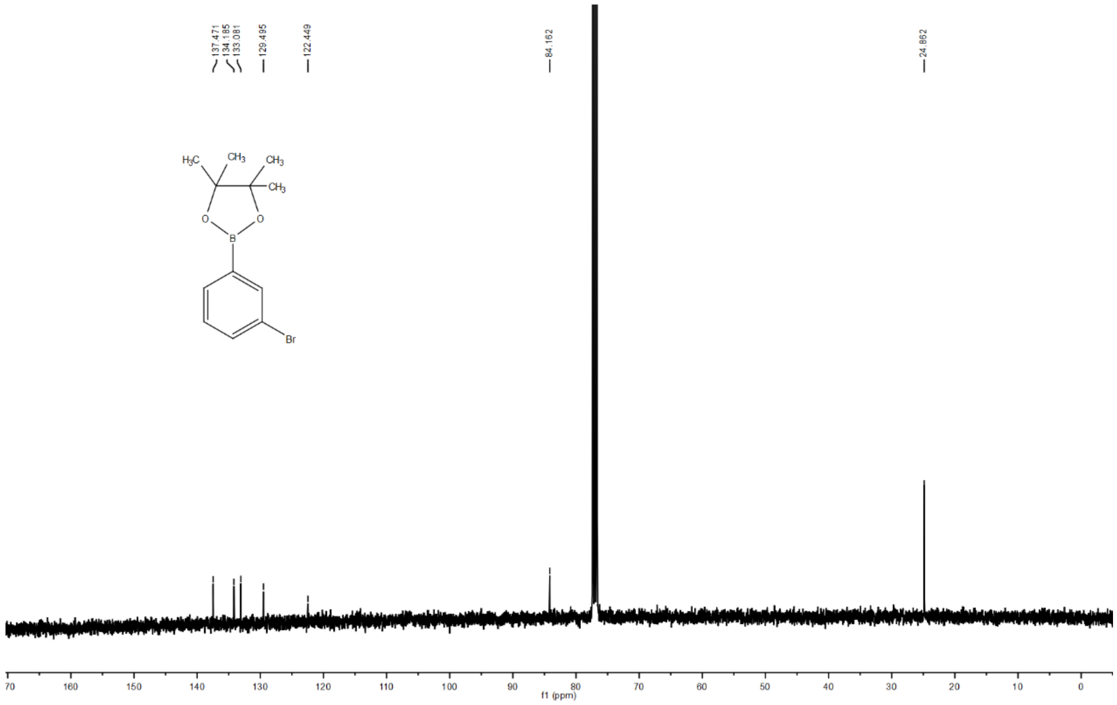

Supplementary Fig. 50.^{13}C NMR (101 MHz, 298 K, $CDCl_3$) of **2t**

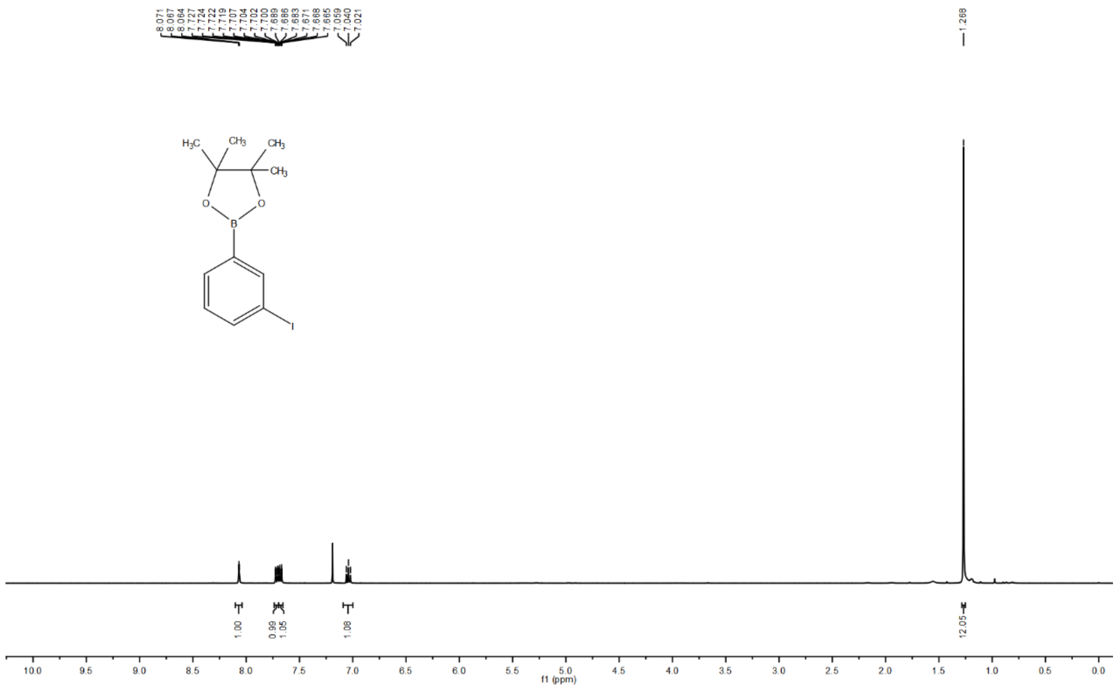

Supplementary Fig. 51.^{1}H NMR (400 MHz, 298 K, $CDCl_3$) of **2u**

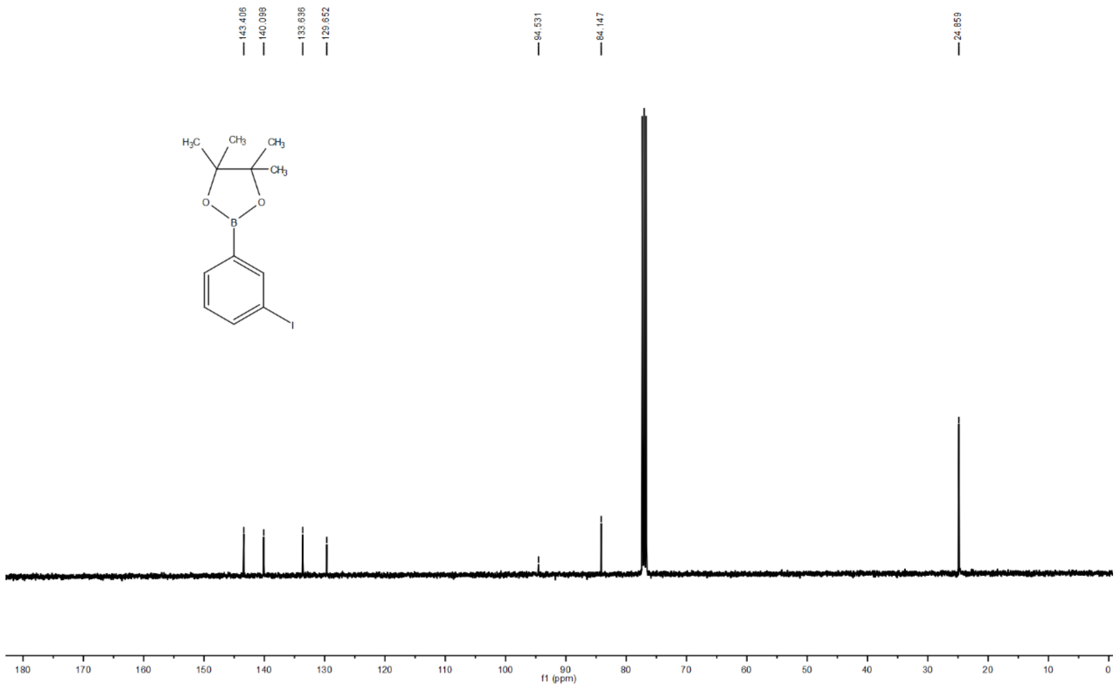

Supplementary Fig. 52.^{13}C NMR (101 MHz, 298 K, $CDCl_3$) of **2u**

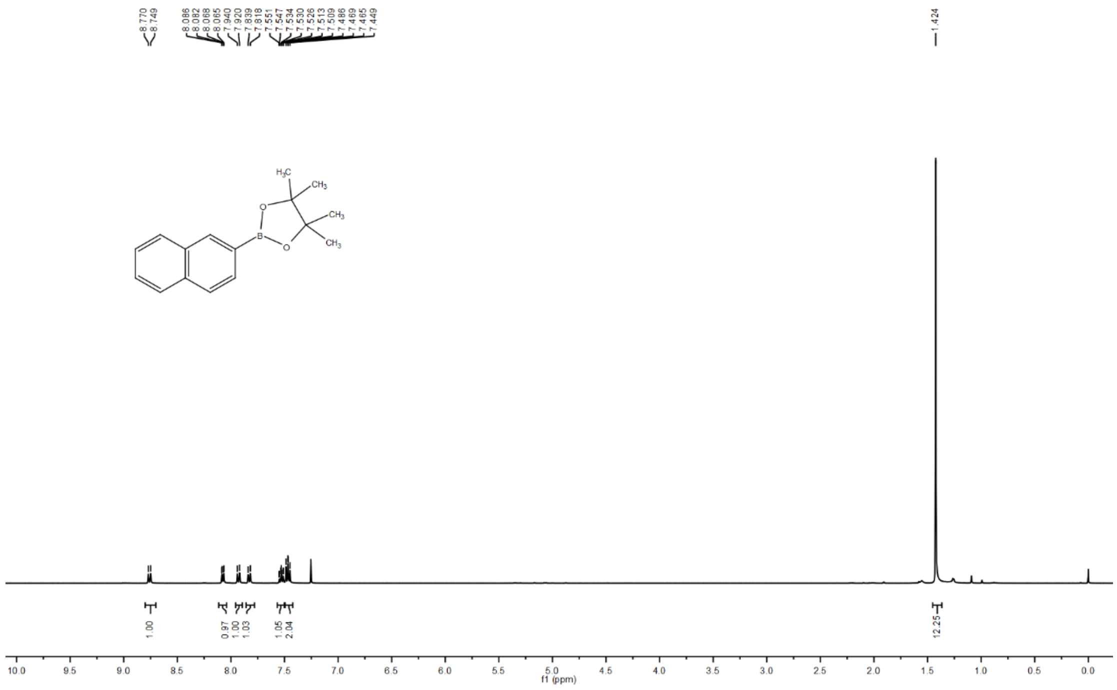

Supplementary Fig. 53. ^{1}H NMR (400 MHz, 298 K, $CDCl_3$) of **2v**

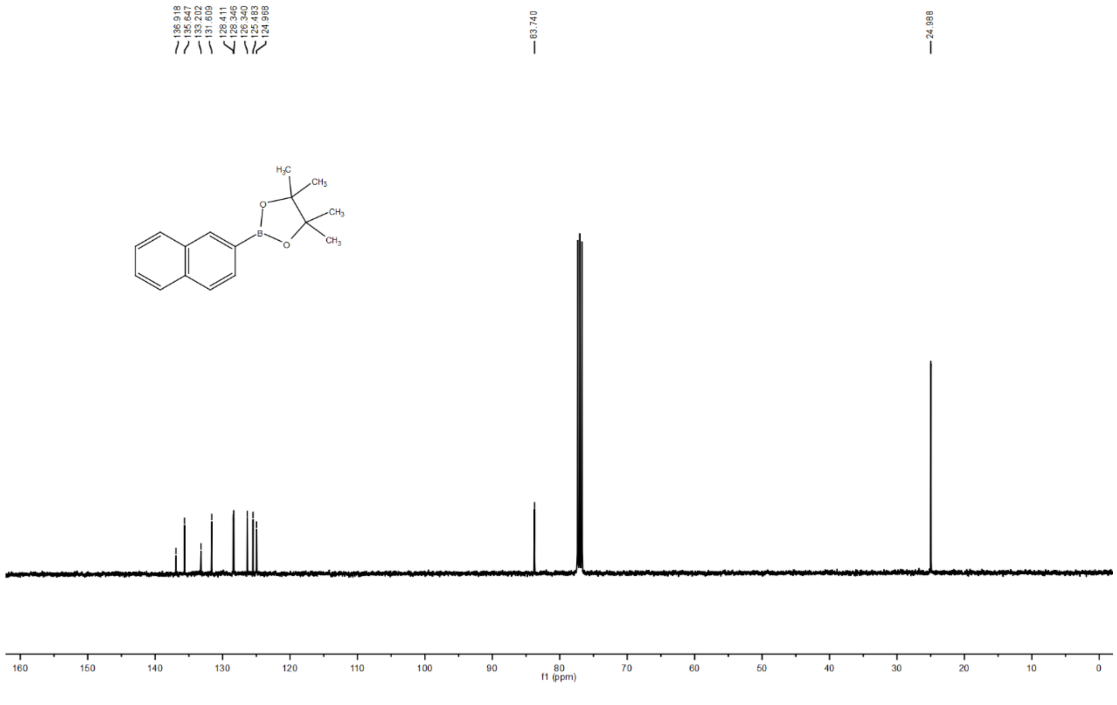

Supplementary Fig. 54. ^{13}C NMR (101 MHz, 298 K, $CDCl_3$) of **2v**

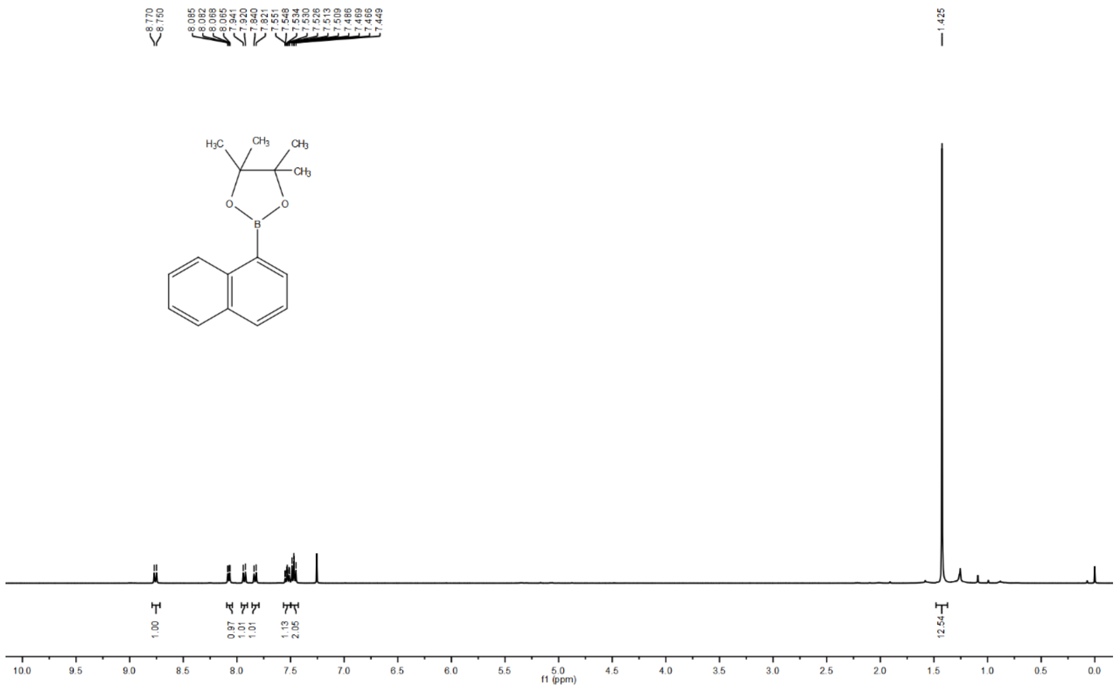

Supplementary Fig. 55.^{1}H NMR (400 MHz, 298 K, $CDCl_3$) of **2w**

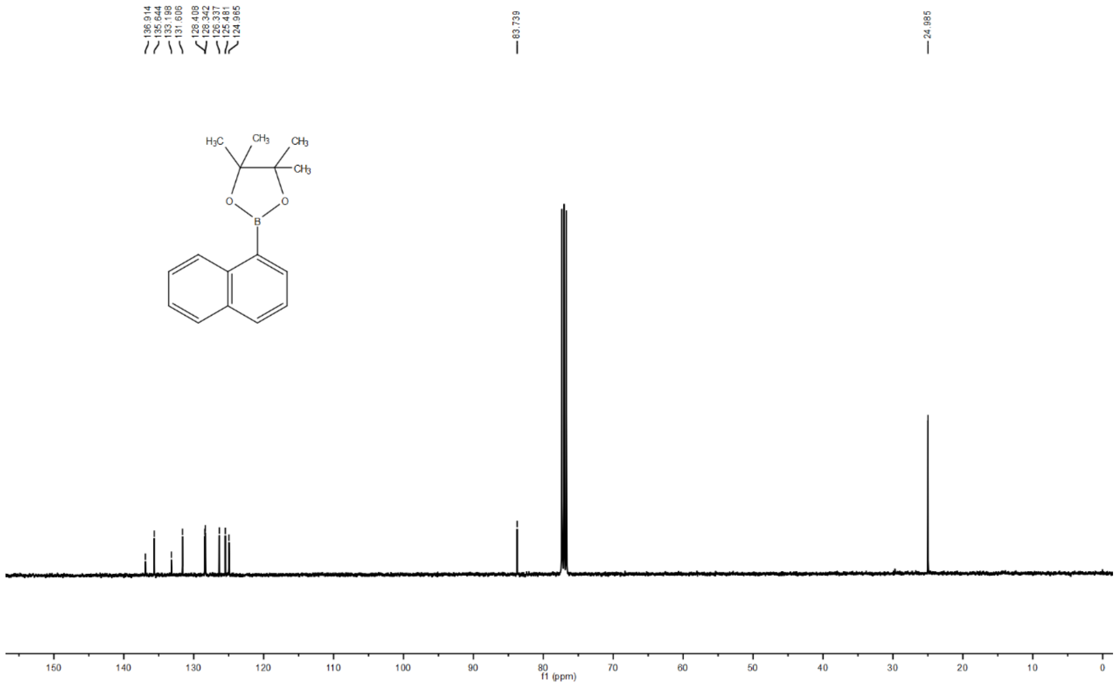

Supplementary Fig. 56.**[13]**C NMR (101 MHz, 298 K, $CDCl_3$) of **2w**

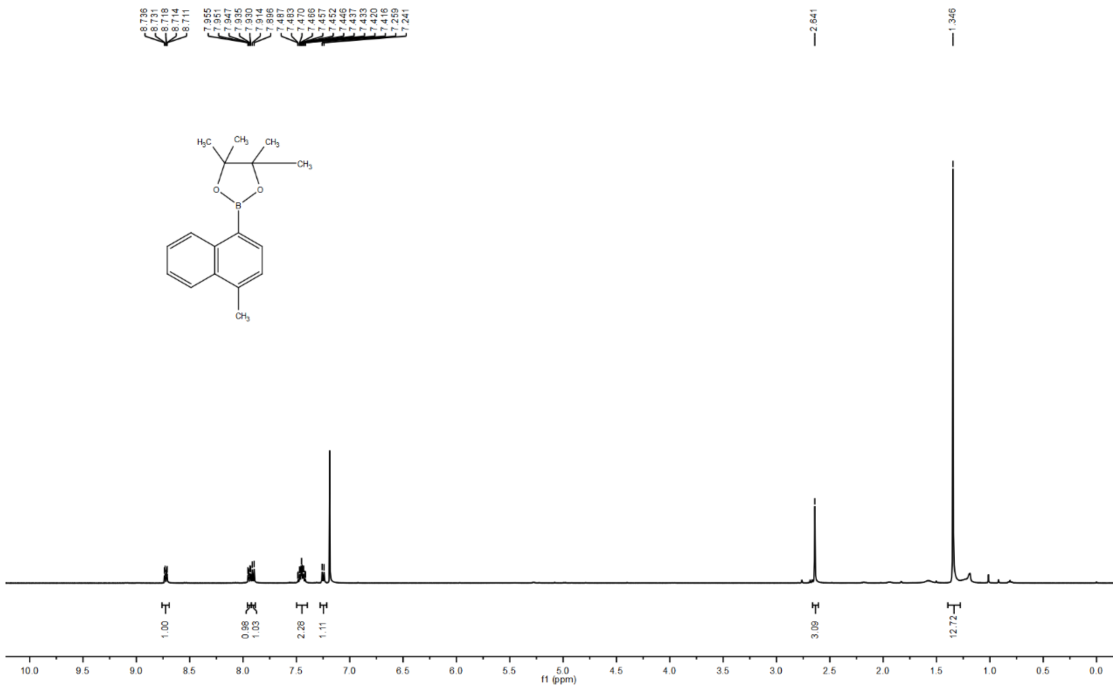

Supplementary Fig. 57.^{1}H NMR (400 MHz, 298 K, $CDCl_3$) of **2x**

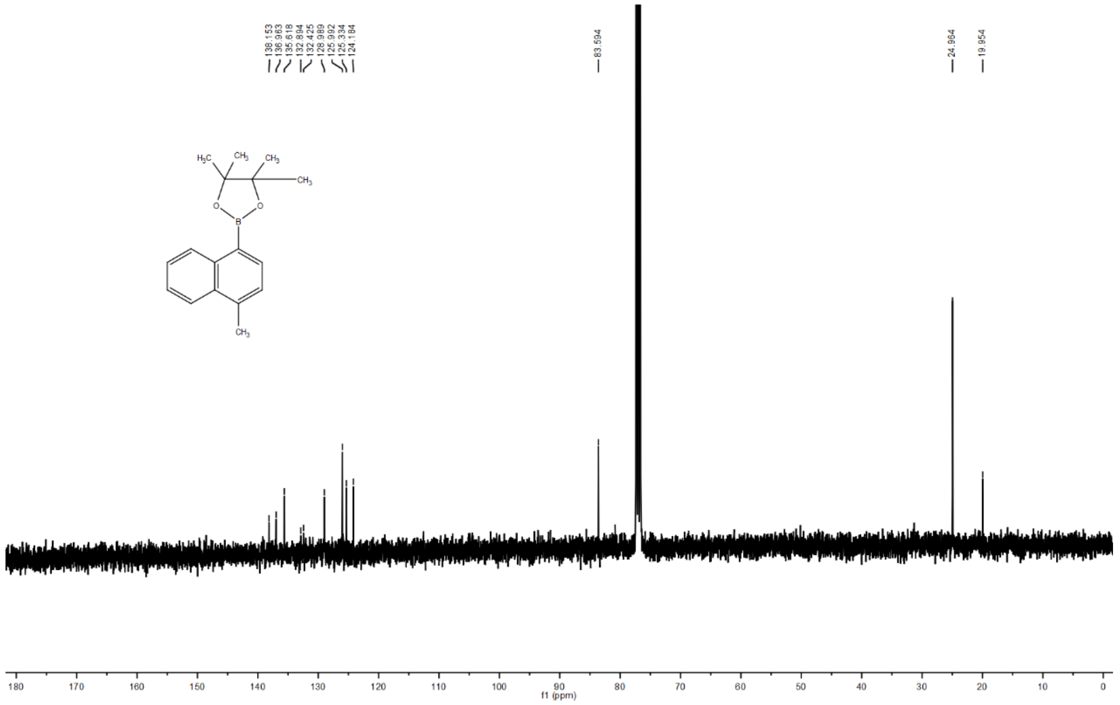

Supplementary Fig. 58.^{13}C NMR (101 MHz, 298 K, $CDCl_3$) of **2x**

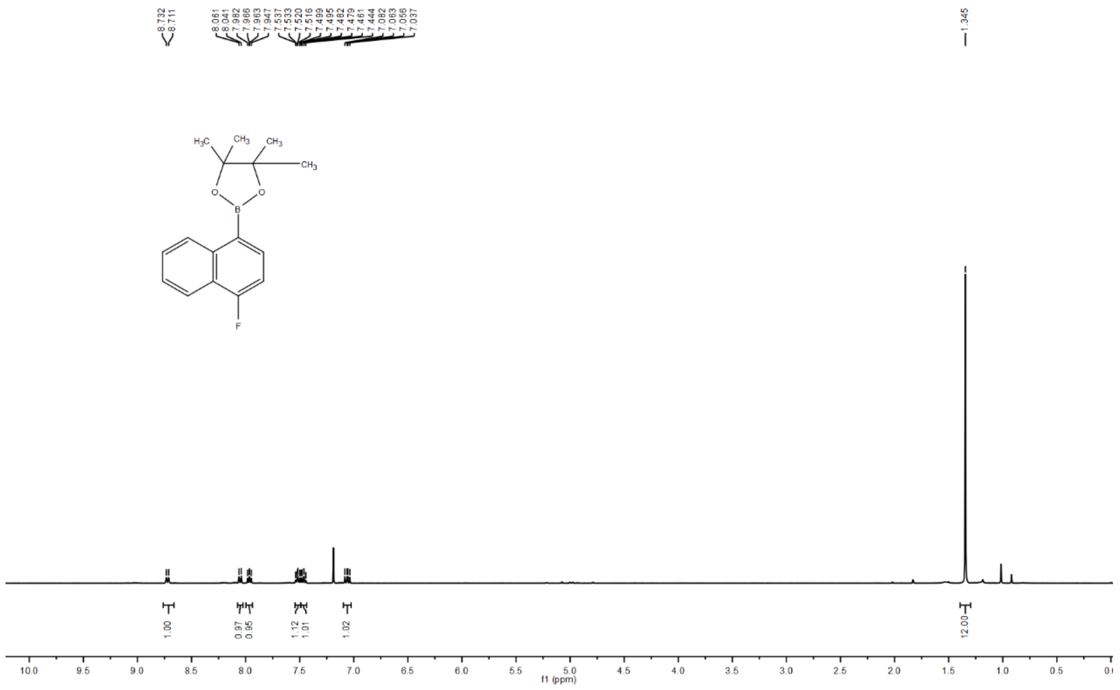

Supplementary Fig. 59.^{1}H NMR (400 MHz, 298 K, $CDCl_3$) of **2y**

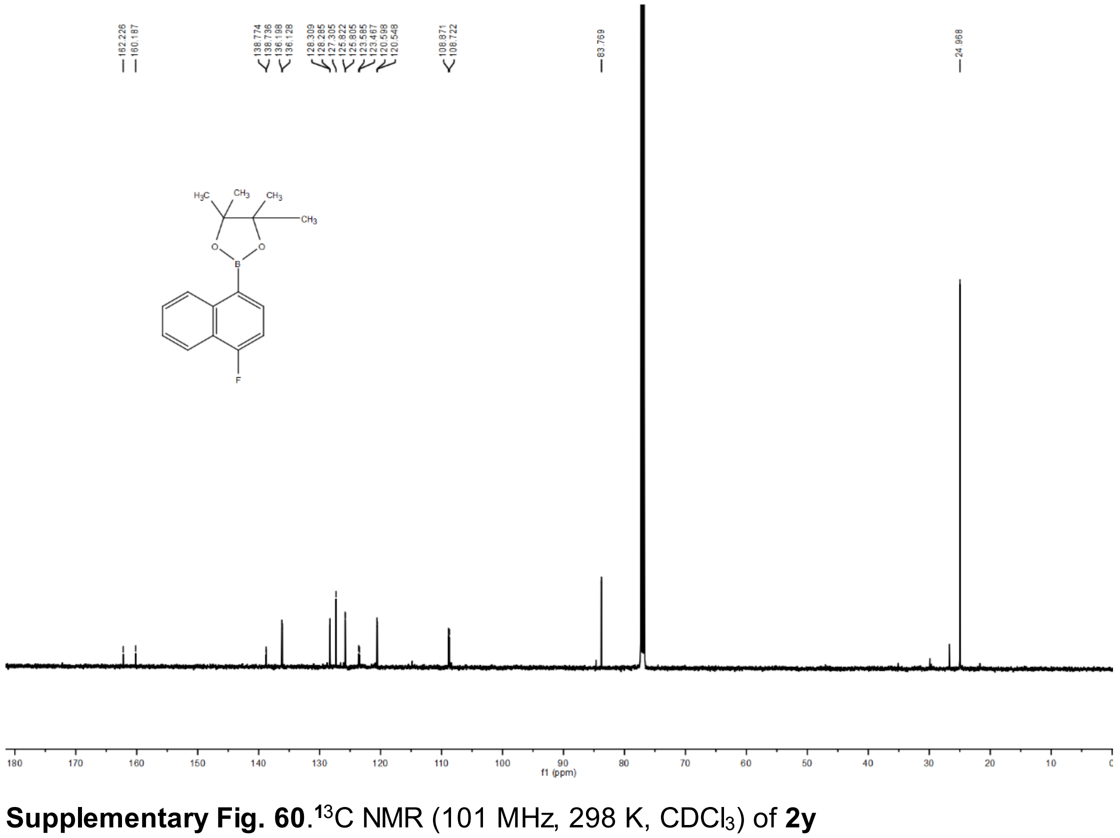

Supplementary Fig. 60.^{13}C NMR (101 MHz, 298 K, $CDCl_3$) of **2y**

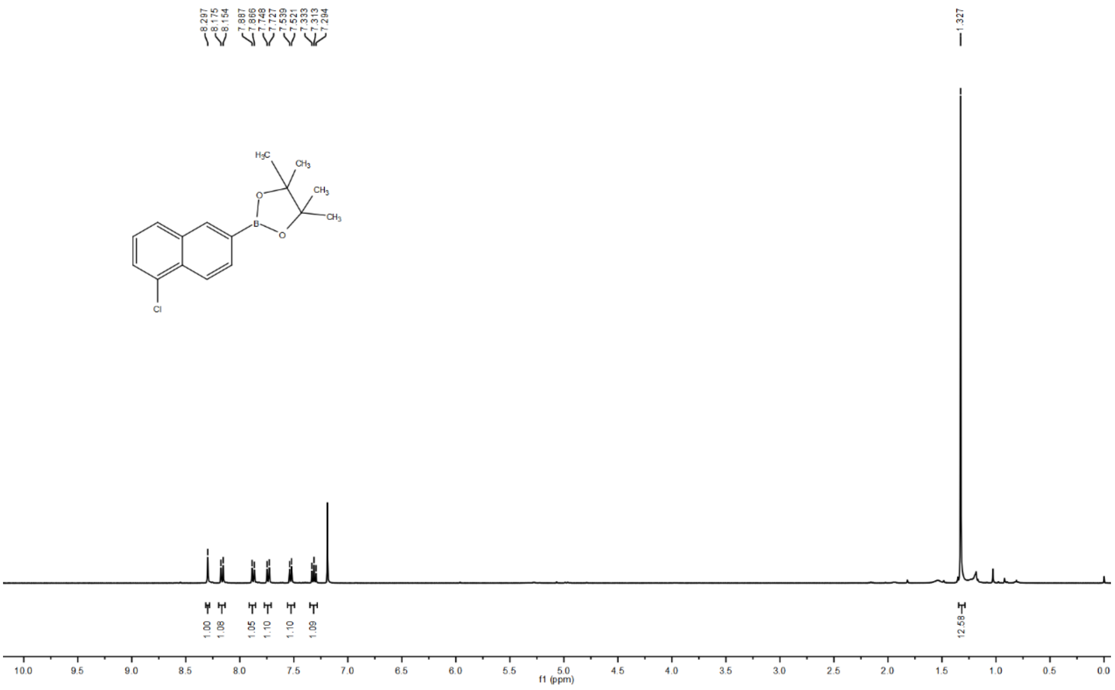

Supplementary Fig. 61.^{1}H NMR (400 MHz, 298 K, $CDCl_3$) of **2z**

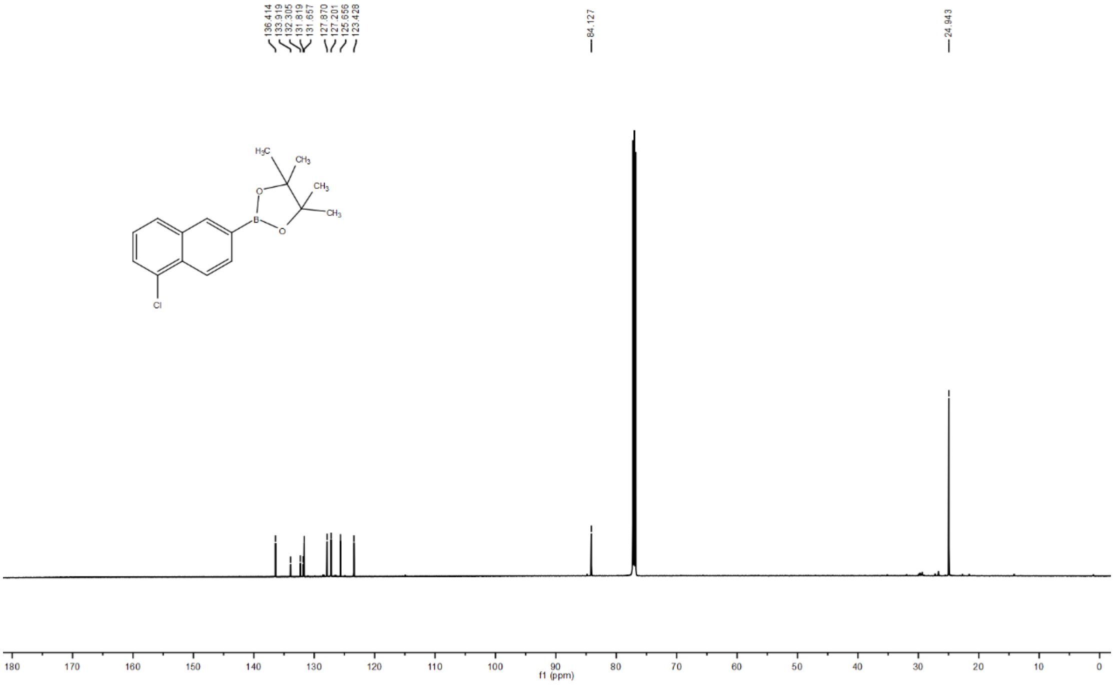

Supplementary Fig. 62.^{13}C NMR (101 MHz, 298 K, $CDCl_3$) of **2z**

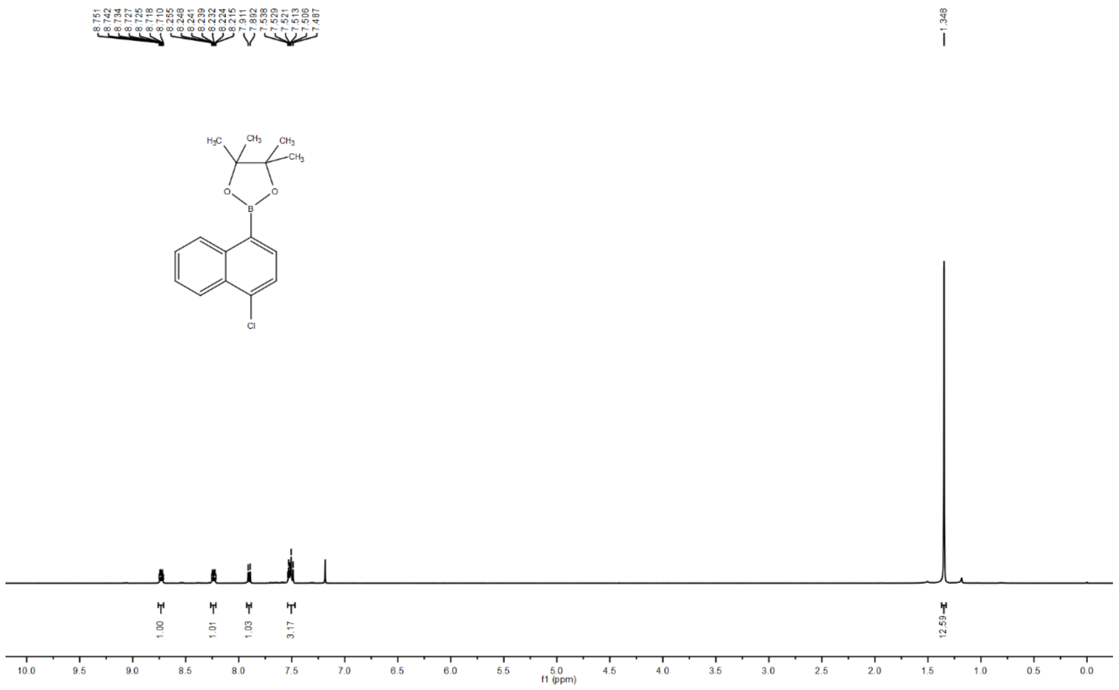

Supplementary Fig. 63.^{1}H NMR (400 MHz, 298 K, $CDCl_3$) of **2aa**

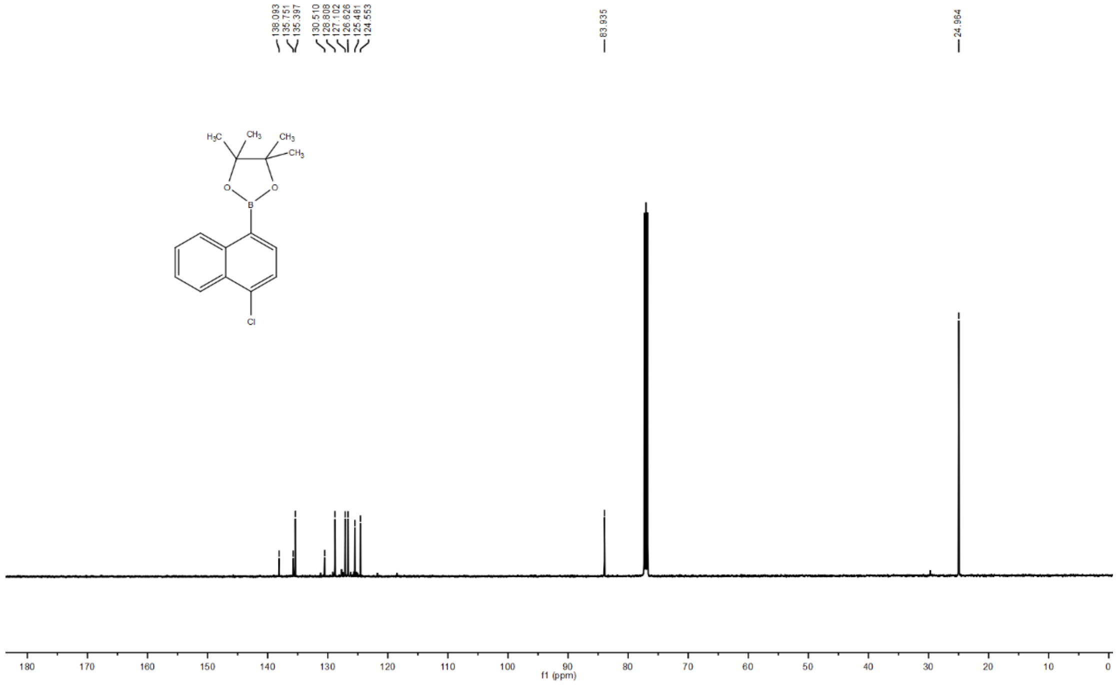

Supplementary Fig. 64.^{13}C NMR (101 MHz, 298 K, $CDCl_3$) of **2aa**

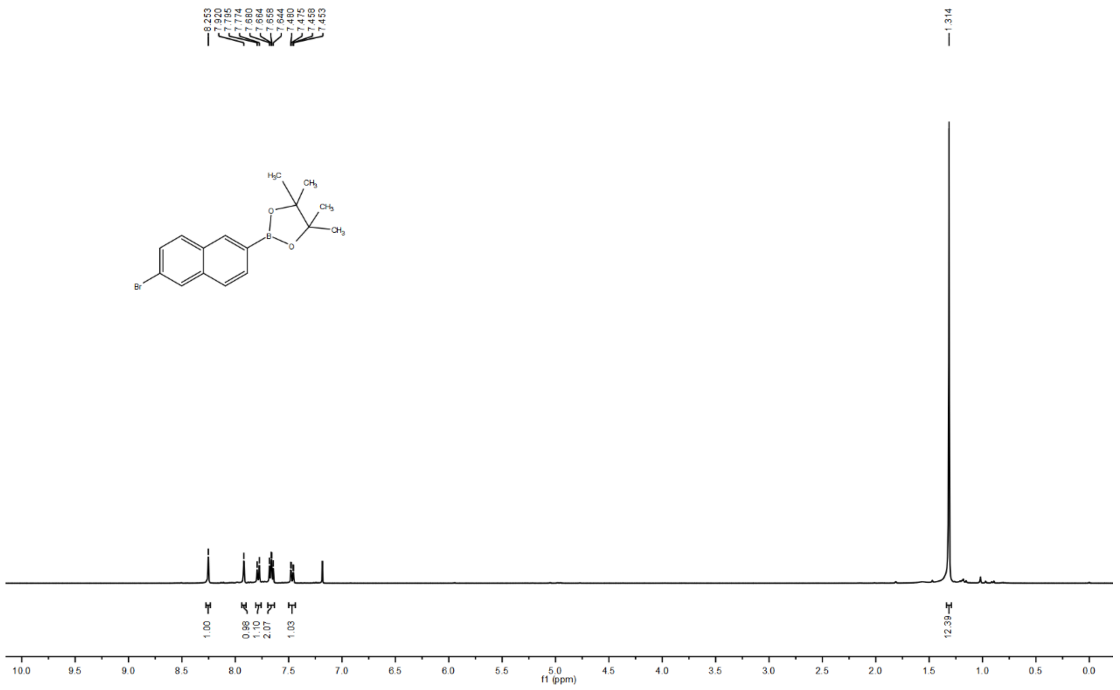

Supplementary Fig. 65.^{1}H NMR (400 MHz, 298 K, $CDCl_3$) of **2ab**

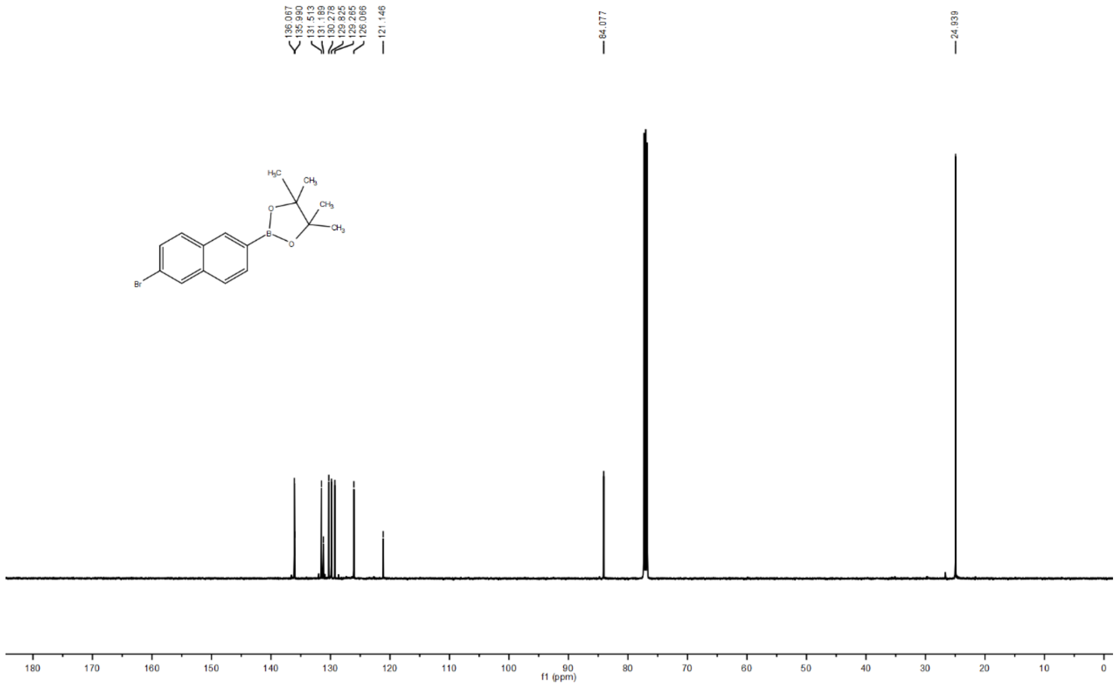

Supplementary Fig. 66.^{13}C NMR (101 MHz, 298 K, $CDCl_3$) of **2ab**

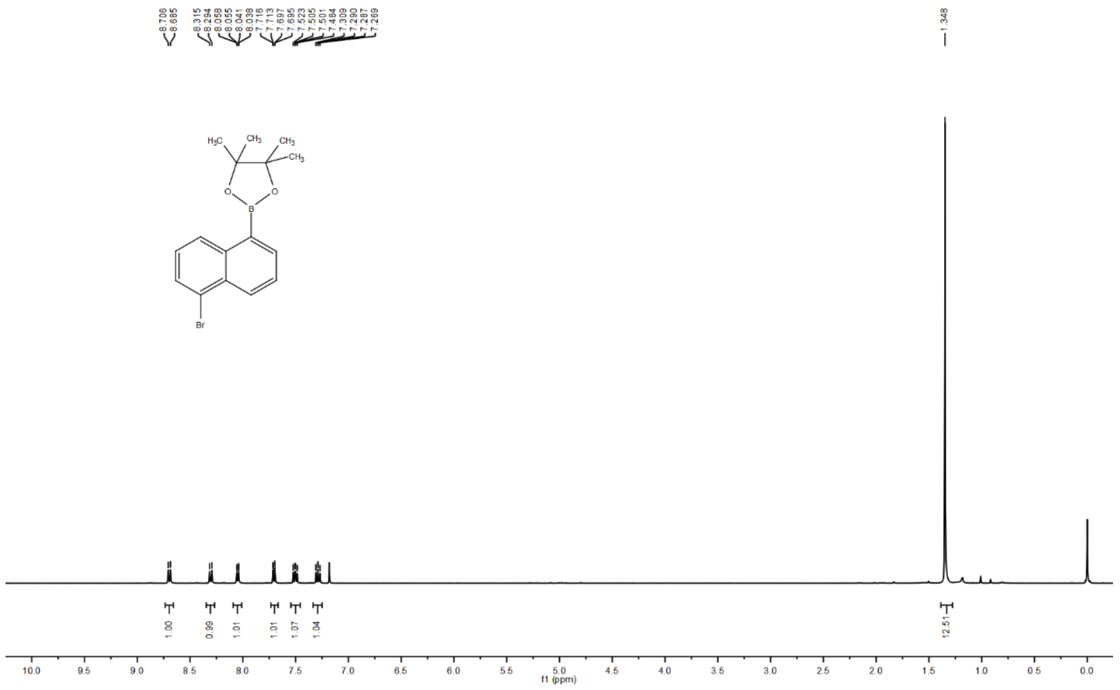

Supplementary Fig. 67.^{1}H NMR (400 MHz, 298 K, $CDCl_3$) of **2ac**

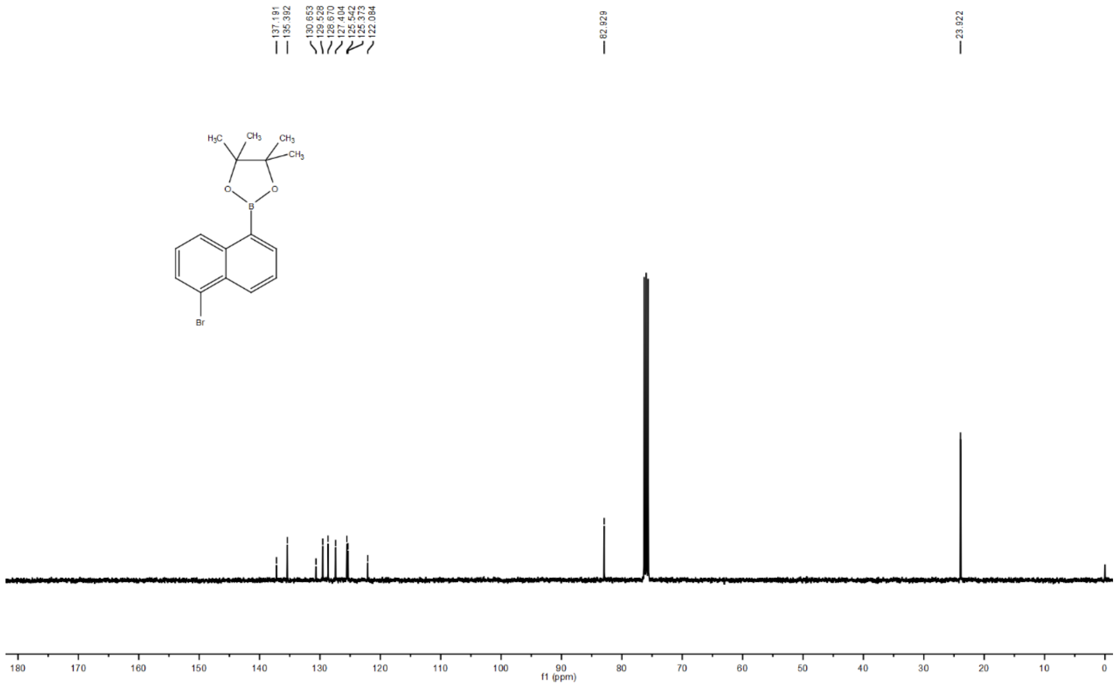

Supplementary Fig. 68.^{13}C NMR (101 MHz, 298 K, $CDCl_3$) of **2ac**

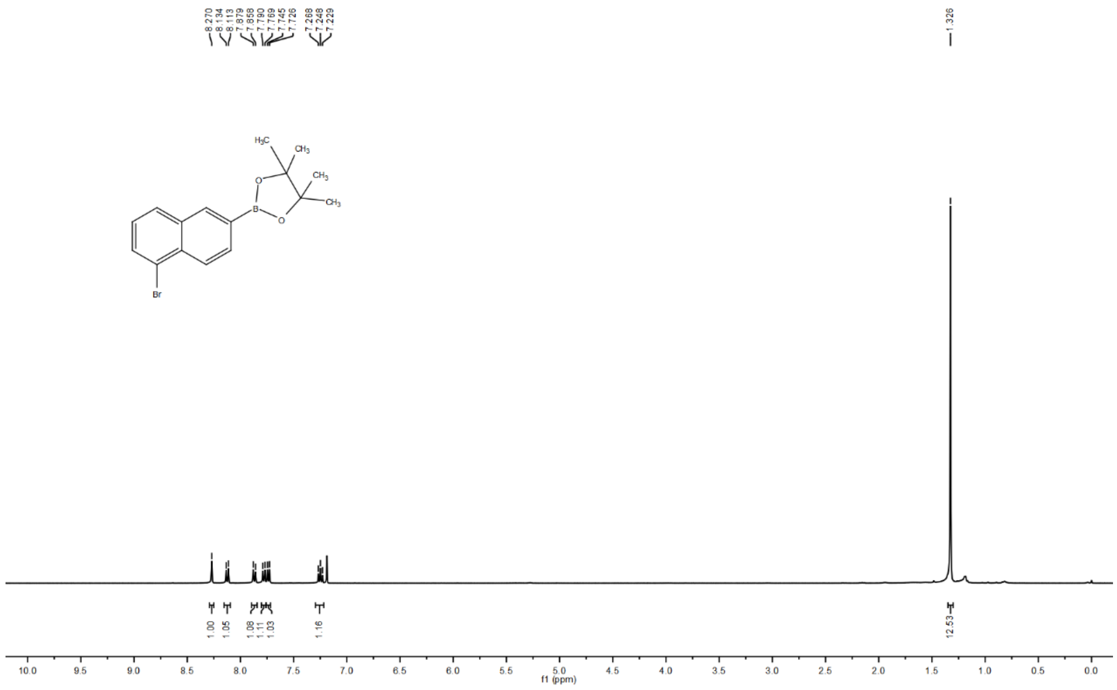

Supplementary Fig. 69.^{1}H NMR (400 MHz, 298 K, $CDCl_3$) of **2ad**

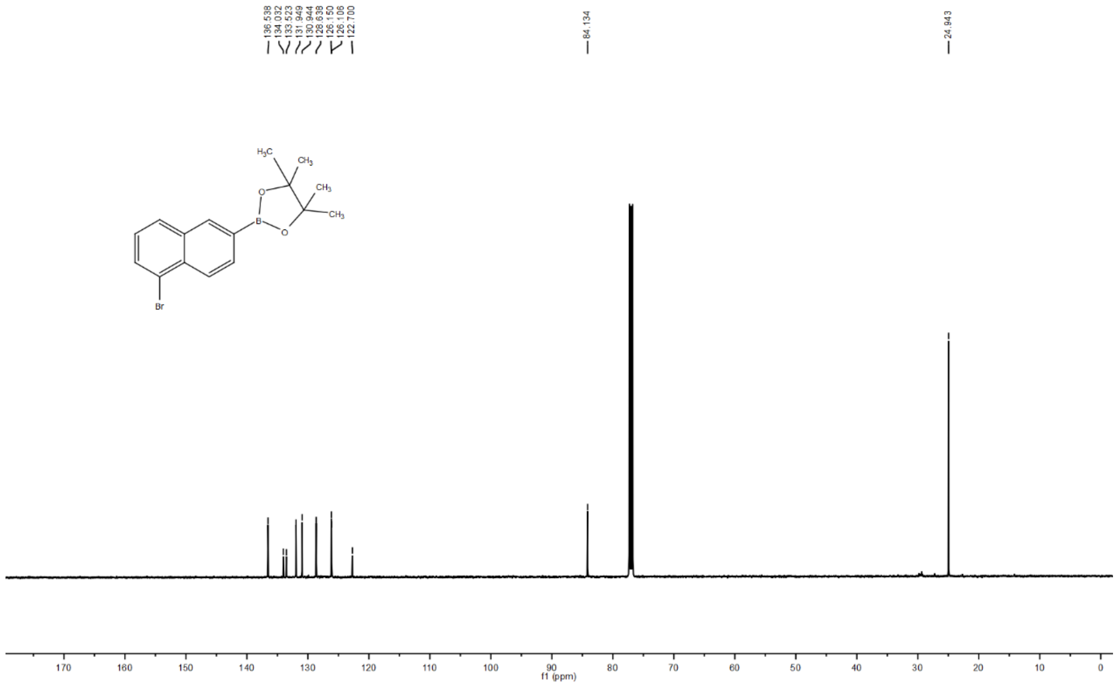

Supplementary Fig. 70.^{13}C NMR (101 MHz, 298 K, $CDCl_3$) of **2ad**

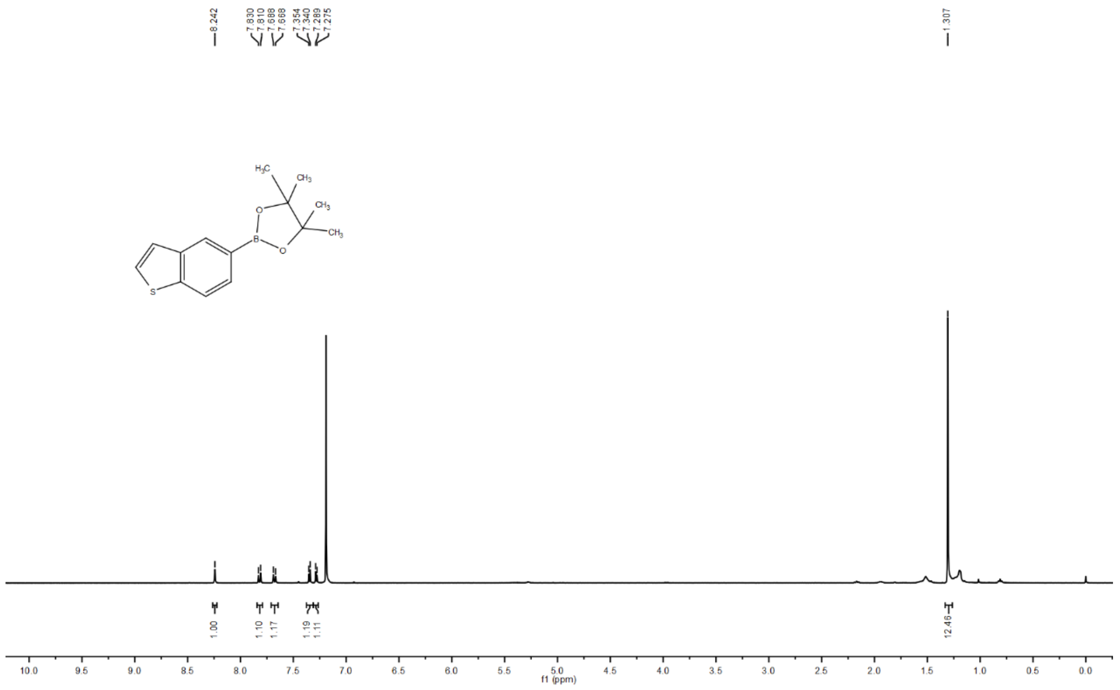

Supplementary Fig. 71.^{1}H NMR (400 MHz, 298 K, $CDCl_3$) of **2ae**

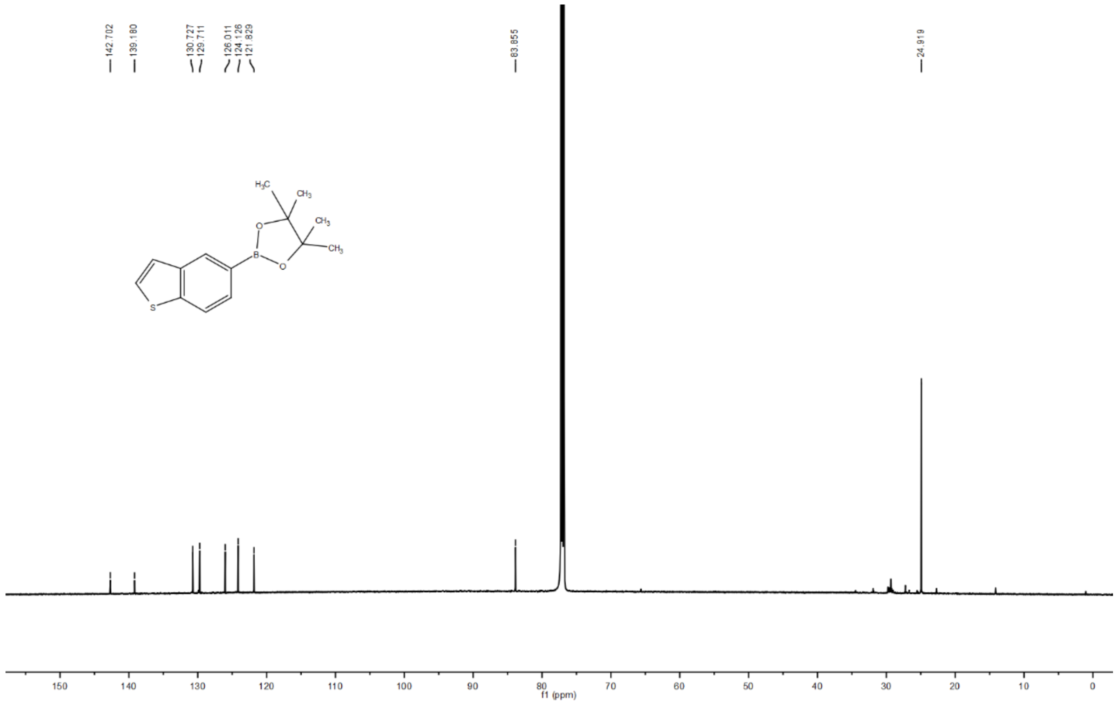

Supplementary Fig. 72.^{13}C NMR (101 MHz, 298 K, $CDCl_3$) of **2ae**

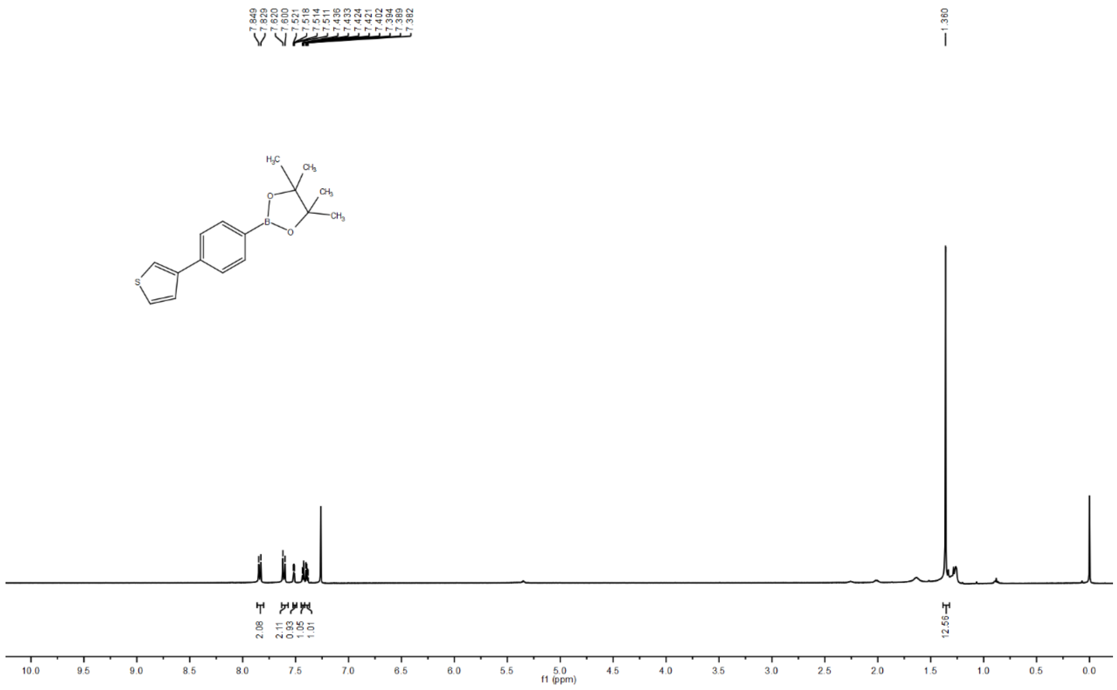

Supplementary Fig. 73.^{1}H NMR (400 MHz, 298 K, $CDCl_3$) of **2af**

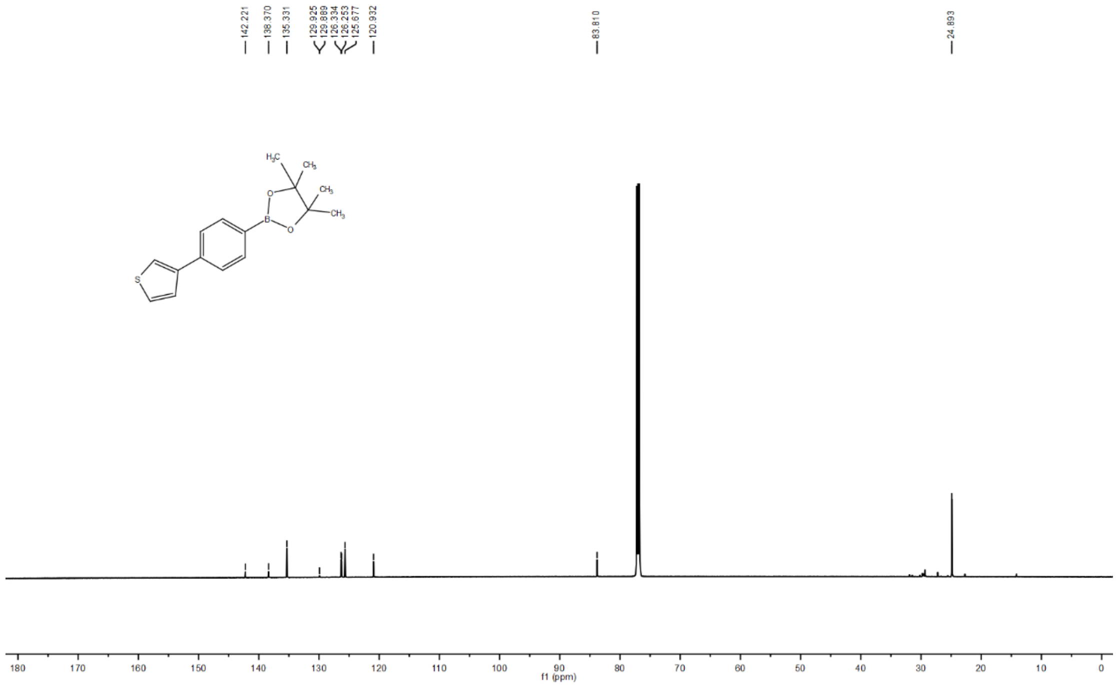

Supplementary Fig. 74.^{13}C NMR (101 MHz, 298 K, $CDCl_3$) of **2af**

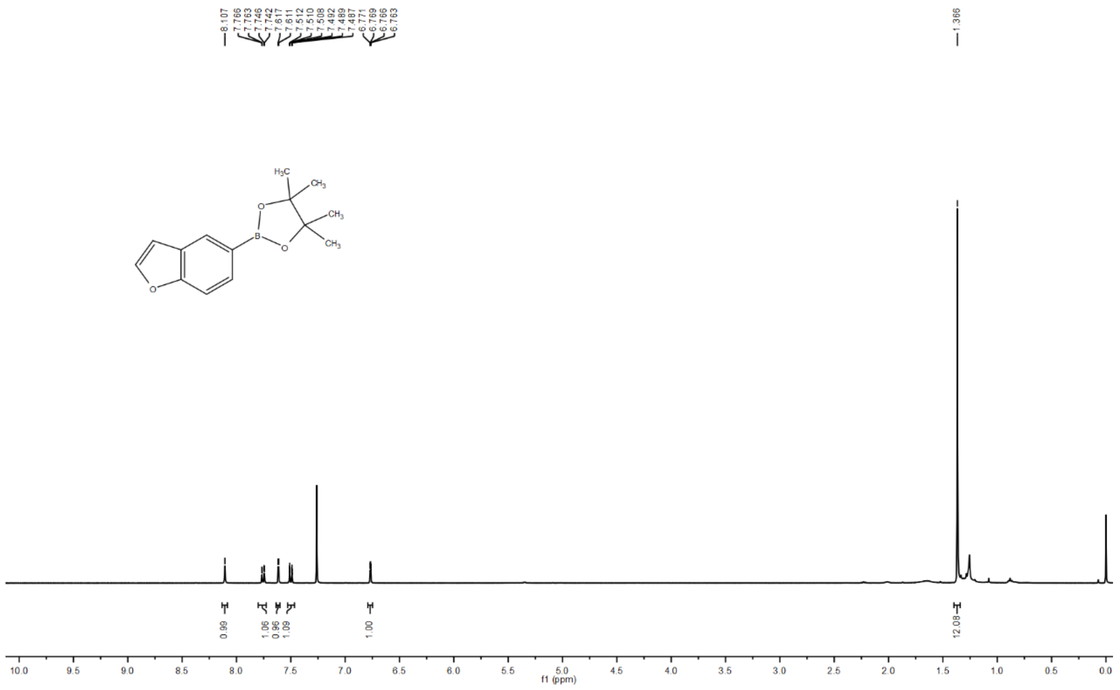

Supplementary Fig. 75.^{1}H NMR (400 MHz, 298 K, $CDCl_3$) of **2ag**

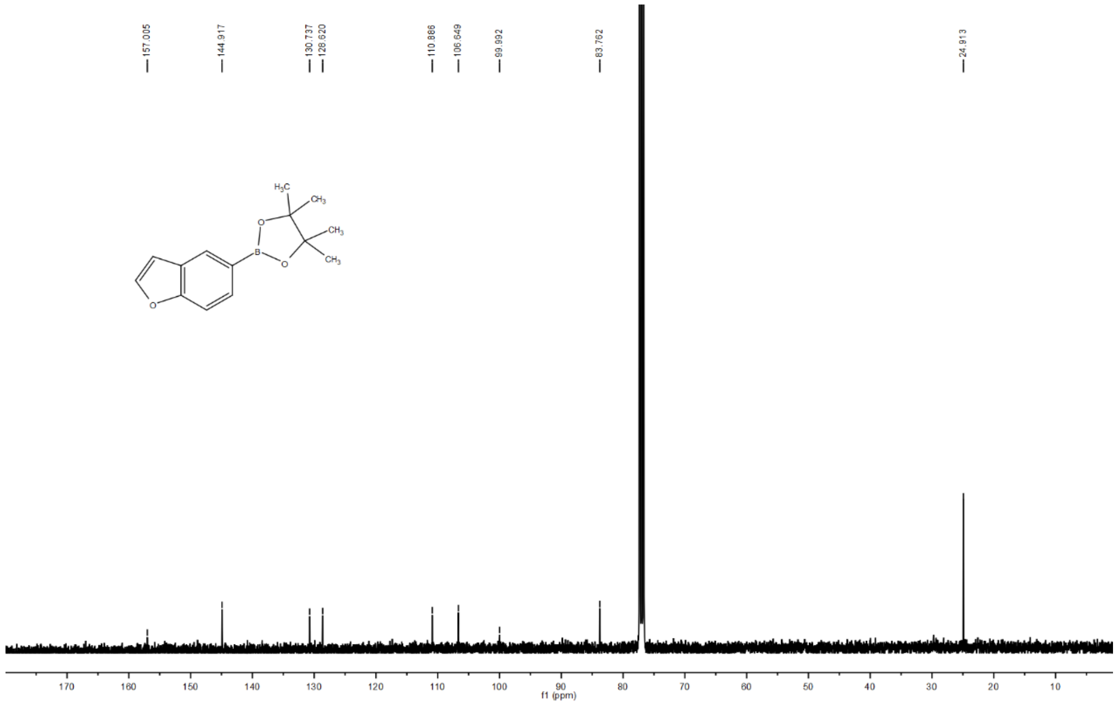

Supplementary Fig. 76.^{13}C NMR (101 MHz, 298 K, $CDCl_3$) of **2ag**

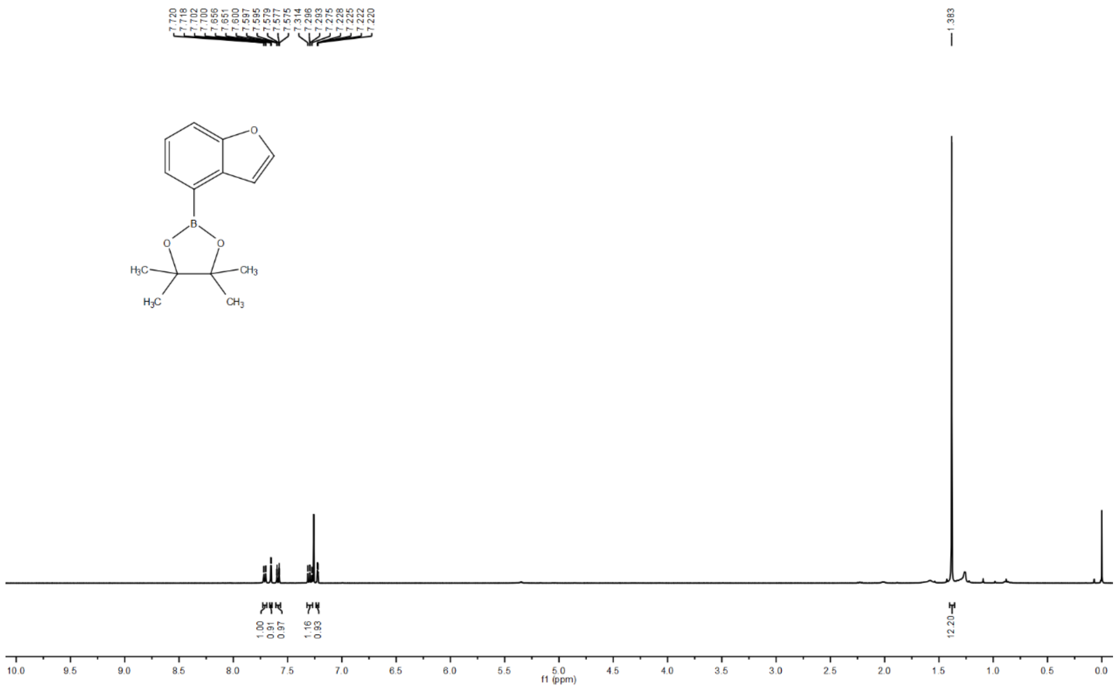

Supplementary Fig. 77.^{1}H NMR (400 MHz, 298 K, $CDCl_3$) of **2ah**

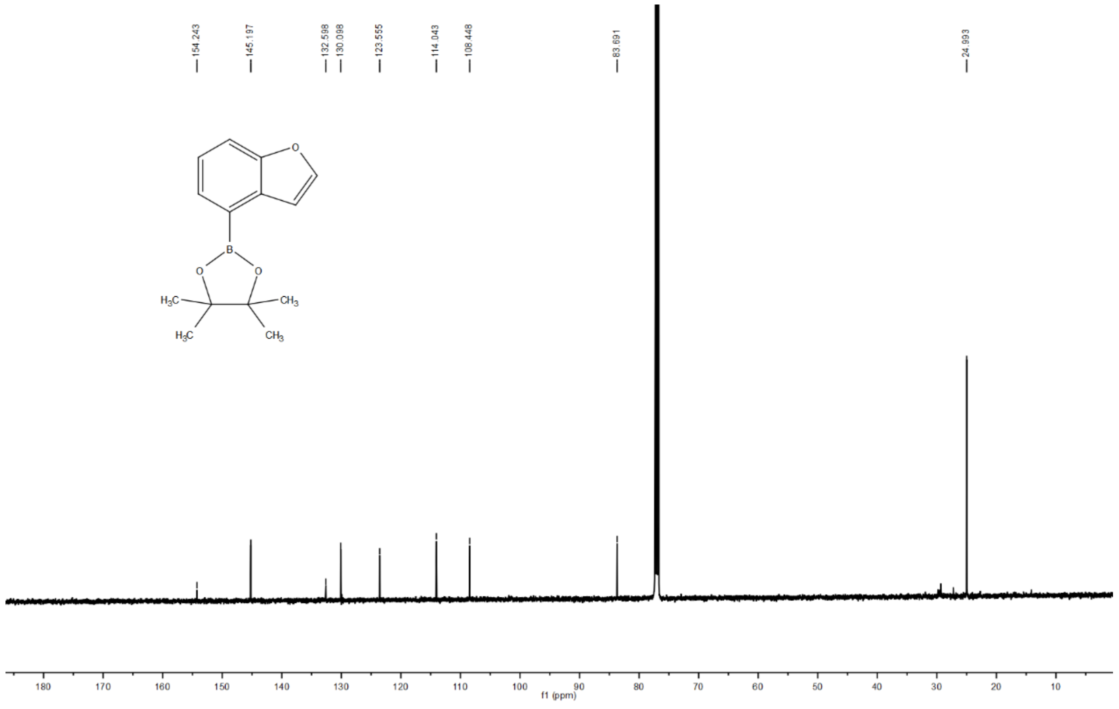

Supplementary Fig. 78.^{13}C NMR (101 MHz, 298 K, $CDCl_3$) of **2ah**

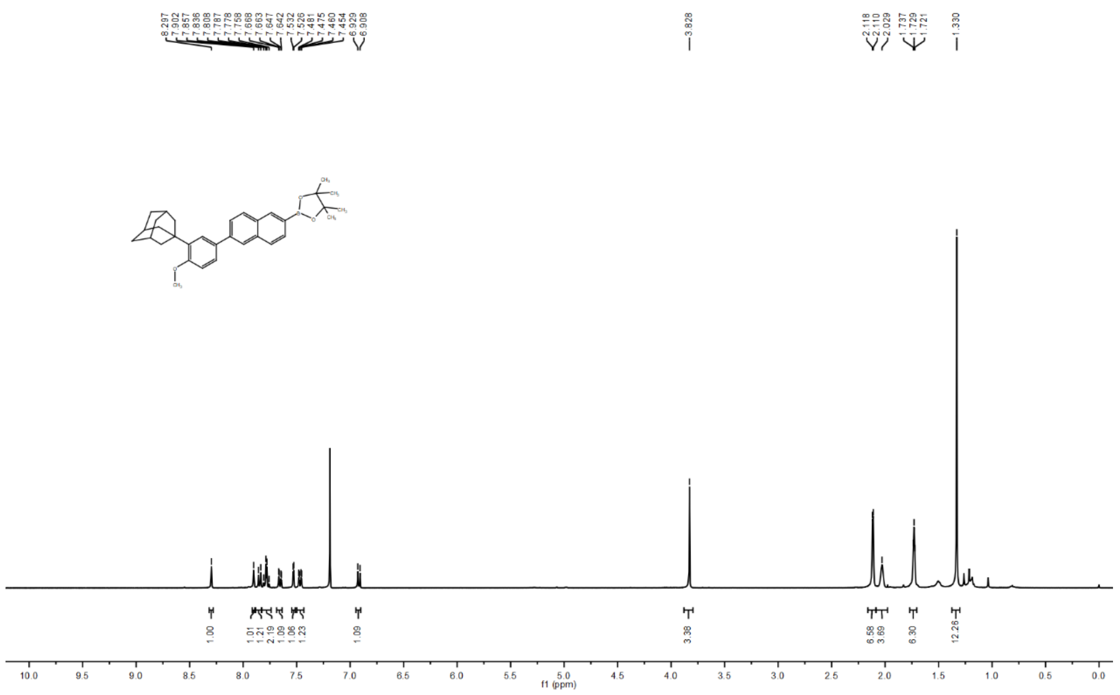

Supplementary Fig. 79.^{1}H NMR (400 MHz, 298 K, $CDCl_3$) of **2ai**

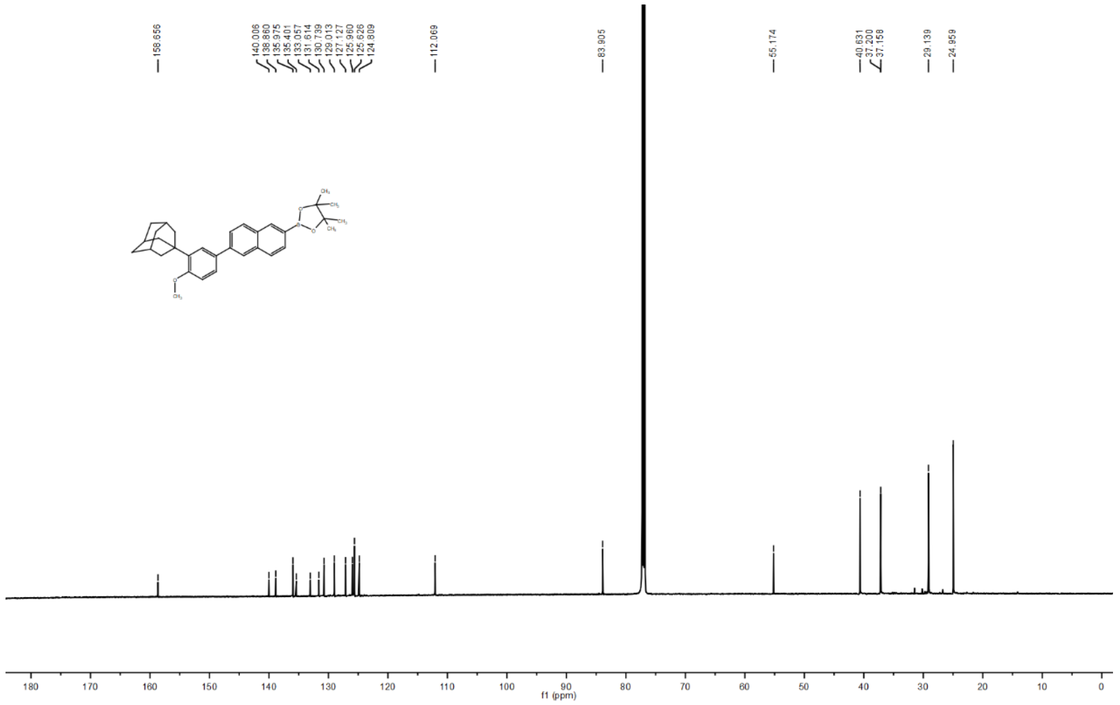

Supplementary Fig. 80.^{13}C NMR (101 MHz, 298 K, $CDCl_3$) of **2ai**

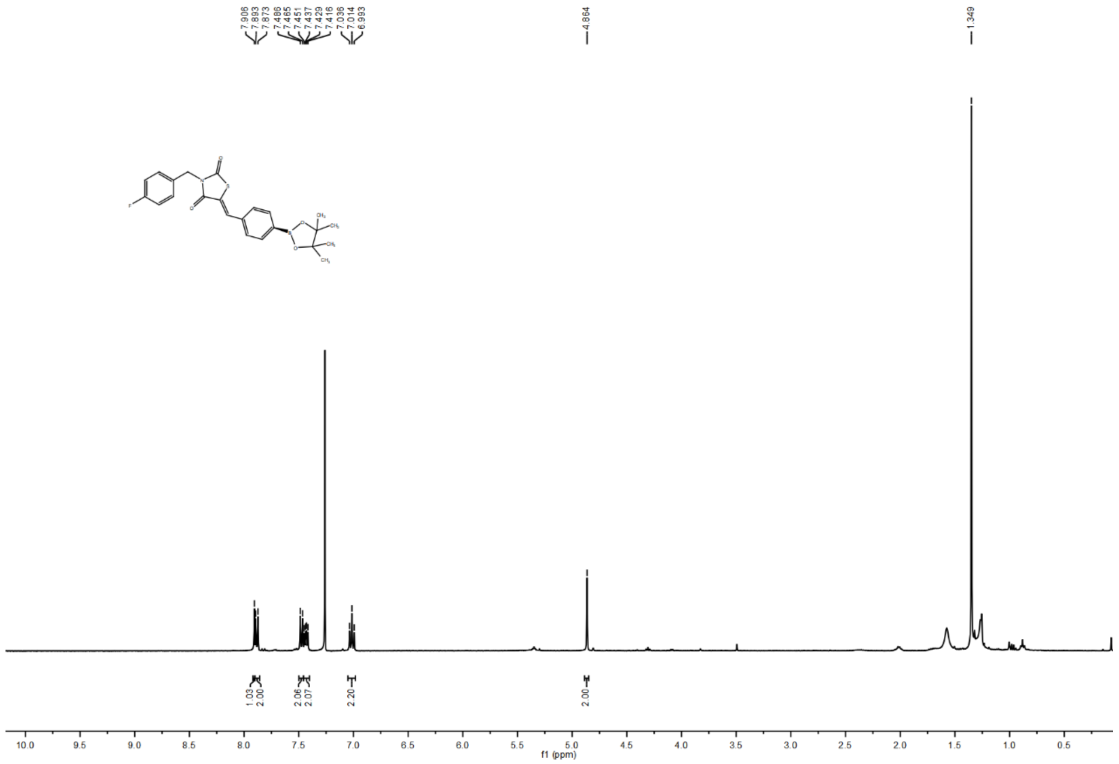

Supplementary Fig. 81.^{1}H NMR (400 MHz, 298 K, $CDCl_3$) of **2aj**

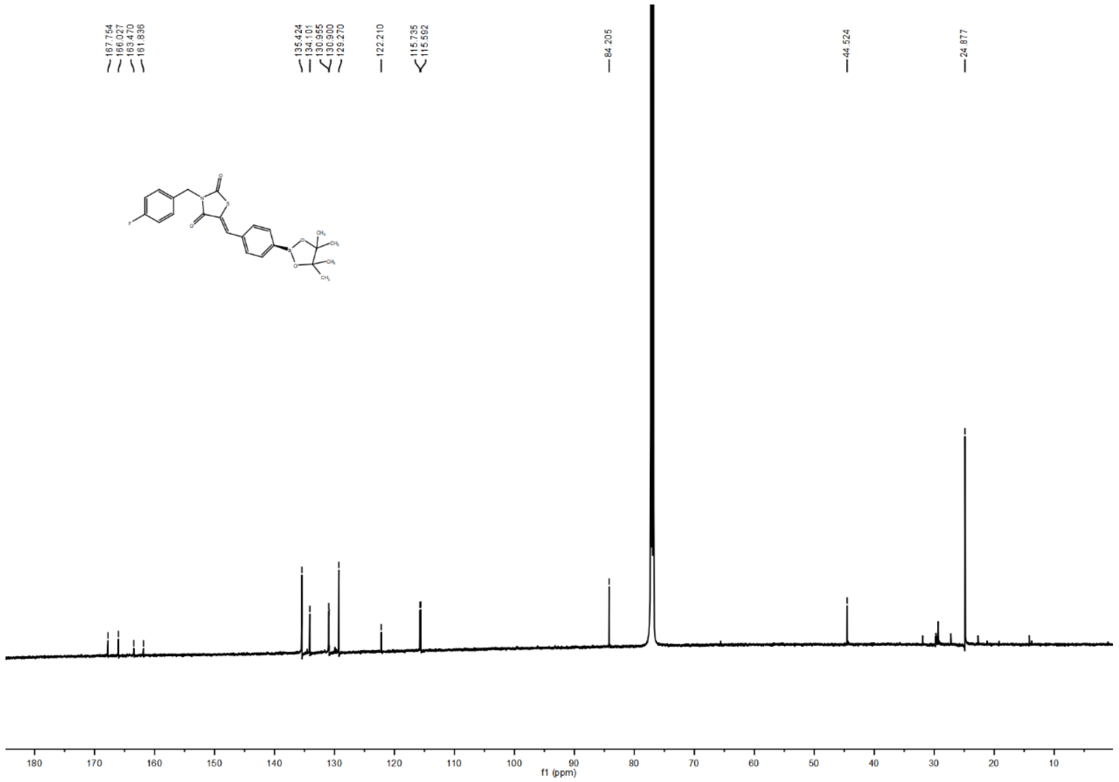

Supplementary Fig. 82.^{13}C NMR (101 MHz, 298 K, $CDCl_3$) of **2aj**

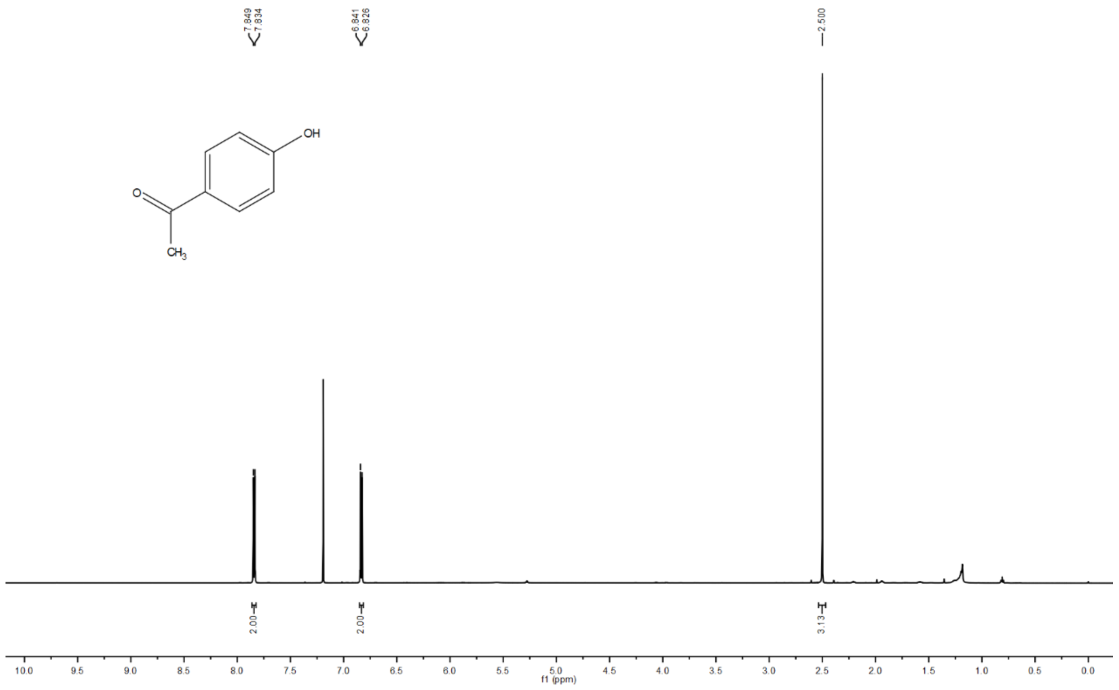

Supplementary Fig. 83.^{1}H NMR (600 MHz, 298 K, $CDCl_3$) of **2o'**

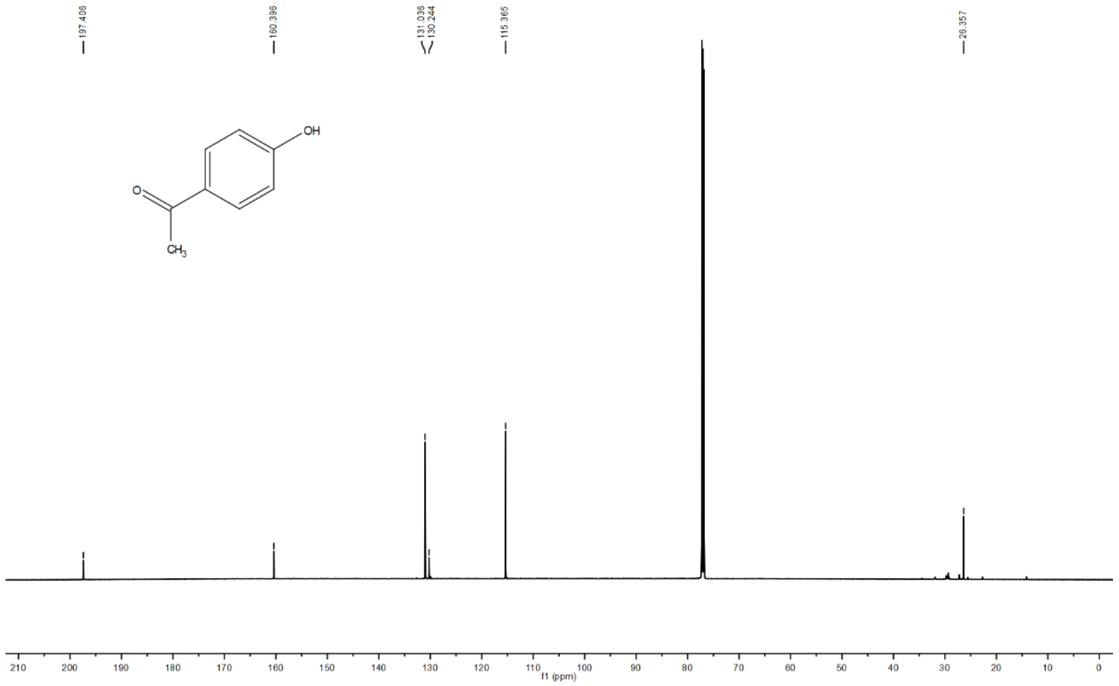

Supplementary Fig. 84.^{13}C NMR (101 MHz, 298 K, $CDCl_3$) of **2o'**

Supplementary Table 21. Cartesian coordinates of important structures

**1a**				**1b**			
C	-5.72300900	-0.06967100	0.04506700	C	-5.65618000	-0.05166300	-0.01781600
C	-4.97098400	-0.92560000	0.85090900	C	-4.92913200	-0.90118700	0.81744700
C	-3.58112200	-0.93724800	0.75224700	C	-3.53689400	-0.92198600	0.75268900
C	-2.93257500	-0.09041300	-0.15503700	C	-2.85844300	-0.09260600	-0.14821900
C	-3.69266200	0.76579400	-0.96053600	C	-3.59439800	0.75657000	-0.98268900
C	-5.08119300	0.77621500	-0.86157700	C	-4.98553300	0.77770900	-0.91886600
H	-6.80483300	-0.06167500	0.12295400	H	-6.73965500	-0.03592400	0.03273400
H	-5.46707400	-1.58363500	1.55588300	H	-5.44685800	-1.54700500	1.51849200
H	-2.99413200	-1.59950200	1.37548400	H	-2.96653200	-1.57837000	1.39763000
H	-3.18256800	1.41740200	-1.65957200	H	-3.06174900	1.39492500	-1.67724000
H	-5.66313500	1.44209600	-1.48926600	H	-5.54713800	1.43901800	-1.56988800
C	-1.43673300	-0.07118800	-0.28773300	C	-1.35128400	-0.09121300	-0.23858000
O	-0.86496300	0.67127700	-1.07442400	O	-0.77175100	0.64608000	-1.03655900
O	-0.82121100	-0.91455800	0.51867300	O	-0.75863600	-0.90901100	0.58393100
C	2.73742900	-0.26930600	0.19323200	C	2.67150300	-0.27294000	0.22032200
N	1.75884600	-1.11742700	0.36443900	N	1.71953500	-1.14727500	0.45460700
H	0.22535700	-0.90003100	0.41653000	H	0.40549500	-0.93691300	0.49938400
N	3.94734200	-0.59503400	-0.38880700	N	3.85216500	-0.60345300	-0.39755500
N	2.59367700	1.03788300	0.59319600	N	2.50556000	1.03136800	0.58718800
C	3.08161600	2.14603400	-0.22877500	C	2.98721100	2.13813900	-0.23964100
H	3.61857000	2.87349400	0.38758500	H	3.61909300	2.81464800	0.34409500
H	3.74673400	1.78782300	-1.01097100	H	3.55417300	1.76892300	-1.09118300
H	2.23520200	2.65368800	-0.70725500	H	2.13041500	2.70419500	-0.62059500
C	1.48225600	1.39863300	1.46650500	C	1.45092600	1.40019200	1.52675700
H	0.56024700	1.57145700	0.90060900	H	0.50498000	1.59177600	1.01113900
H	1.30574900	0.61142400	2.19788500	H	1.30259200	0.60737300	2.25805200
H	1.74560800	2.31800100	1.99596200	H	1.75984500	2.30861400	2.05017300
H	2.04703200	-2.08013100	0.22341500	H	2.01012500	-2.11218100	0.35022600
C	5.21157500	-0.08567100	0.14718500	C	5.13373500	-0.04201500	0.03466300
H	5.85447800	0.27306700	-0.66202600	H	5.68360100	0.37023700	-0.81642700
H	5.03617700	0.72988600	0.84464800	H	4.98372500	0.74350400	0.77162400
H	5.74057500	-0.88457400	0.68232100	H	5.74323400	-0.82898400	0.49427800
C	4.07589200	-1.86090200	-1.09839300	C	3.96212600	-1.86558300	-1.11947400
H	4.22284200	-2.71117200	-0.41665400	H	4.18072100	-2.70997100	-0.45113300
H	3.19550400	-2.04703300	-1.71499400	H	3.04469800	-2.07340600	-1.67110300
H	4.94506000	-1.80236000	-1.75689400	H	4.77970200	-1.78197700	-1.83847000

1b’				1c			
C	-5.58758400	0.03010300	-0.32088800	C	-5.71040500	-0.00840900	-0.03877700
C	-4.98759500	-1.12819400	0.17494700	C	-5.00930700	-0.93600900	0.73394100
C	-3.60326500	-1.19567900	0.30742300	C	-3.61666300	-0.98238100	0.67749500
C	-2.81097100	-0.10012000	-0.06048300	C	-2.90609700	-0.10492200	-0.14977100
C	-3.41760600	1.05980200	-0.55926200	C	-3.61818100	0.82106000	-0.92070600
C	-4.80115800	1.12427100	-0.68717300	C	-5.01041900	0.87086600	-0.86737600
H	-6.66601700	0.08019300	-0.42276700	H	-6.79388000	0.02900700	0.00427300
H	-5.59802400	-1.97844600	0.45727400	H	-5.54829100	-1.62179900	1.37951600
H	-3.13642900	-2.09350600	0.69121700	H	-3.06145800	-1.69701300	1.27279100
H	-2.79653700	1.90068400	-0.84278300	H	-3.06248300	1.49777800	-1.55881600
H	-5.26746100	2.02400700	-1.07220400	H	-5.55022000	1.59390500	-1.47017200
C	-1.32798100	-0.13286300	0.05709400	C	-1.38168700	-0.14519800	-0.21166300
O	-0.60162900	0.78344100	-0.29852900	O	-0.80203200	0.66595300	-0.96381700
O	-0.85088100	-1.25868900	0.59079600	O	-0.81435200	-1.01073800	0.52938600
C	2.72534900	-0.40982500	0.20347400	C	2.73923800	-0.27912100	0.20654300
N	1.90253800	-1.35926700	0.52243400	N	1.83654500	-1.23546800	0.40258500
H	0.14705000	-1.23274400	0.60367300	H	0.78909600	-1.05309300	0.44595000
N	3.89995500	-0.47436600	-0.44030400	N	3.93456100	-0.56247800	-0.36799000
N	2.21853300	0.89706000	0.49851700	N	2.47037400	0.98659300	0.58913400
C	2.26981400	1.96469400	-0.47955400	C	2.94398700	2.14759000	-0.16824400
H	2.91223000	2.76989800	-0.10344800	H	3.58982000	2.78031400	0.44692400
H	2.64112800	1.59728100	-1.43156300	H	3.48617500	1.83580000	-1.05793800
H	1.25475500	2.35544200	-0.58496200	H	2.07669600	2.73316600	-0.48499700
C	1.65007400	1.18084200	1.79700800	C	1.40043700	1.27791500	1.54651400
H	0.82257100	1.87931800	1.68579000	H	0.45178900	1.43816500	1.02947800
H	1.34222900	0.25827300	2.28417100	H	1.29120400	0.45700000	2.25319200
H	2.43736700	1.65170300	2.40496500	H	1.67691700	2.18031400	2.09633300
H	2.23348300	-2.28343000	0.24772500	H	2.12766300	-2.19862700	0.32425000
C	4.96360600	0.52869200	-0.28832500	C	5.17489800	0.10510700	0.03487800
H	5.13244100	1.05883000	-1.22834300	H	5.62183600	0.64450500	-0.80453300
H	4.72185800	1.24602100	0.49379900	H	4.99117100	0.80088500	0.85034000
H	5.88217300	0.01461000	0.00204900	H	5.88531400	-0.65082800	0.38321600
C	4.29453400	-1.72175700	-1.10121000	C	4.11787300	-1.77924600	-1.15691600
H	4.74124900	-2.42356700	-0.38993300	H	4.40805700	-2.63143600	-0.53029400
H	3.43596400	-2.18701100	-1.58741300	H	3.20662200	-2.02327600	-1.70261600
H	5.02956200	-1.47934800	-1.86822000	H	4.91264700	-1.60052600	-1.88306100

**1c’**				**1d**			
C	-5.36241600	0.13143900	-0.36795400	C	-5.87870700	0.08434500	0.18985000
C	-4.82729300	-1.06484600	0.11279200	C	-5.16466900	-0.91049400	0.86215800
C	-3.45069700	-1.20092700	0.26828300	C	-3.79345600	-1.02900800	0.67053600
C	-2.60291100	-0.13567500	-0.06037600	C	-3.14200200	-0.14457200	-0.19960000
C	-3.14232100	1.06235600	-0.54553100	C	-3.85641900	0.85402400	-0.87547200
C	-4.51918000	1.19489300	-0.69550400	C	-5.22726200	0.96489200	-0.67715100
H	-6.43522300	0.23469600	-0.48773000	H	-6.94842200	0.17372100	0.34228800
H	-5.48244600	-1.89067800	0.36583700	H	-5.67788000	-1.59044800	1.53162200
H	-3.02794700	-2.12551200	0.64026000	H	-3.22977800	-1.79969500	1.18371500
H	-2.47987800	1.88073500	-0.79925900	H	-3.34177400	1.53377300	-1.54514600
H	-4.93552500	2.12410900	-1.06740900	H	-5.78812400	1.73437600	-1.19405900
C	-1.12621600	-0.27643300	0.09661900	C	-1.70304100	-0.26645700	-0.40172300
O	-0.36719700	0.61760600	-0.34407000	O	-1.01656900	0.43718400	-1.20670100
O	-0.68750700	-1.33163300	0.68749300	O	-0.94137500	-1.08450500	0.19156300
C	2.48724700	-0.39861400	0.15932000	C	2.94805800	-0.25454300	0.20110400
N	1.77896400	-1.44021700	0.47680100	N	2.06424400	-1.25502900	0.32498900
H	0.50117600	-1.38603700	0.62246500	H	1.06541100	-1.08550900	0.30359400
N	3.65429000	-0.39612100	-0.49819300	N	4.18868100	-0.51401000	-0.24920200
N	1.91830400	0.87192000	0.50608200	N	2.58405200	0.99584200	0.53919700
C	2.04748400	2.03312900	-0.36093900	C	3.09832800	2.17891300	-0.16043400
H	2.76744800	2.73674600	0.06943700	H	3.69824700	2.80120600	0.50778300
H	2.35533700	1.73426400	-1.35847200	H	3.69582600	1.88871800	-1.02155100
H	1.07293000	2.52041300	-0.40260400	H	2.24572500	2.76324500	-0.51594700
C	1.46967700	1.10481500	1.87061000	C	1.44802400	1.25061500	1.42951900
H	0.67182500	1.84524700	1.86042800	H	0.52883400	1.42107000	0.86103700
H	1.13535400	0.17767900	2.32777800	H	1.30516000	0.41851600	2.11716200
H	2.31936600	1.49942100	2.44436600	H	1.66802500	2.14710200	2.01185500
H	2.22440000	-2.34046400	0.33424200	H	2.37016400	-2.21625400	0.31292100
C	4.70372800	0.61795300	-0.30435300	C	5.36695700	0.22617200	0.21724000
H	4.88563500	1.15949400	-1.23432900	H	5.82678800	0.78587400	-0.60045900
H	4.44378100	1.31535300	0.48699700	H	5.10067100	0.90932200	1.02004000
H	5.61940800	0.10389700	-0.00459500	H	6.09301100	-0.49349800	0.60388700
C	4.10194700	-1.62560800	-1.16021100	C	4.48396000	-1.73494300	-1.00337000
H	4.58487500	-2.30501900	-0.45026200	H	4.76456500	-2.55852900	-0.33782000
H	3.25865300	-2.12751600	-1.63548100	H	3.62809200	-2.02834800	-1.61025600
H	4.82326000	-1.34856800	-1.92841900	H	5.32155700	-1.52735000	-1.67038800

**1d’**				**1d-TS**			
C	-5.29244000	0.24481900	-0.47588200	C	0.00000000	0.00000000	-2.56737000
C	-4.82858000	-1.03253600	-0.14681400	C	-0.00248300	1.21153800	-1.87652900
C	-3.47219500	-1.24709400	0.06928200	C	0.00000000	1.22945200	-0.47478400
C	-2.57447400	-0.17711700	-0.04190000	C	0.00000000	0.00000000	0.14175300
C	-3.03800100	1.10169000	-0.38229500	C	0.00000000	-1.22945200	-0.47478400
C	-4.39845800	1.31021800	-0.58937300	C	0.00248300	-1.21153800	-1.87652900
H	-6.35133800	0.40758000	-0.64313000	H	0.00000000	0.00000000	-3.65145800
H	-5.52668100	-1.85699700	-0.05688900	H	-0.00222700	2.15328200	-2.41446300
H	-3.10031400	-2.23020100	0.33078400	H	0.00482700	2.15607900	0.08226500
H	-2.33788600	1.92345700	-0.46831200	H	-0.00482700	-2.15607900	0.08226500
H	-4.76150500	2.30066400	-0.83864100	H	0.00222700	-2.15328200	-2.41446300
C	-1.12226700	-0.43014500	0.20694700	C	0.00000000	0.00000000	2.10765700
O	-0.32983700	0.34951400	-0.44691700	O	-0.15163400	-1.14586500	2.40246000
O	-0.72886000	-1.33213000	0.96286500	O	0.15163400	1.14586500	2.40246000
C	2.51023100	-0.35674300	0.18761600				
N	1.97593600	-1.50610700	0.57081600				
H	0.96525400	-1.54622100	0.80070900				
N	3.61755400	-0.30523200	-0.55213800				
N	1.83984500	0.80479500	0.55471500				
C	1.89156000	2.01953200	-0.25755600				
H	2.61007500	2.73252300	0.15513900				
H	2.14397600	1.78090400	-1.28723400				
H	0.90013900	2.47123500	-0.23472800				
C	1.37722100	0.99445800	1.93365600				
H	0.39365800	1.46312600	1.91846300				
H	1.32411200	0.04721400	2.46112000				
H	2.08006800	1.65930400	2.44777300				
H	2.49539500	-2.36824100	0.48795300				
C	4.59414200	0.79421000	-0.48208800				
H	4.60378300	1.35902100	-1.41558300				
H	4.38331000	1.45377400	0.35447700				
H	5.57913800	0.35117800	-0.32301700				
C	4.08670400	-1.49994500	-1.26518300				
H	4.70682500	-2.12674600	-0.61685600				
H	3.24034900	-2.07436600	-1.63994200				
H	4.68721800	-1.16854200	-2.11188700				

1e-TS				1e-TS-3			
B	0.60942000	-0.94800200	0.07993500	C	-1.57430300	3.84463900	-0.28374400
O	1.37142900	-0.97177000	1.22323600	C	-1.16822200	3.18156600	-1.44444300
O	1.35216400	-1.19286100	-1.05047000	C	-0.22567400	2.15957100	-1.36887300
C	2.78120500	-1.01740600	0.82845200	C	-0.09110400	2.45591000	1.02179300
C	2.69832200	-1.58950900	-0.63576200	C	-1.03137300	3.48017400	0.95018800
B	-1.07169700	-0.70012000	0.05809900	H	-2.31058800	4.63943400	-0.34080500
O	-1.84400500	-0.56482400	1.18807500	H	-1.58752300	3.46330500	-2.40467600
O	-1.82426200	-0.69808300	-1.09377100	H	0.08054600	1.64152500	-2.27097500
C	-3.21111300	-0.24414200	0.77238000	H	0.32131300	2.16933100	1.98306100
C	-3.23662400	-0.75507500	-0.71732200	H	-1.34536300	3.99306400	1.85323600
C	0.07374000	1.84035700	-0.16916800	B	1.42007700	0.65759800	-0.05294500
C	0.25605200	2.49945000	1.02237800	O	2.06192300	0.31075600	1.12120400
C	0.54934600	2.23160400	-1.39718800	O	2.03780200	0.13144300	-1.17050500
C	1.00159500	3.68668700	0.96967200	C	3.29735500	-0.37840700	0.76517300
H	-0.13748300	2.12246000	1.95927100	C	3.01304700	-0.85241400	-0.71266100
C	1.29297800	3.42183800	-1.42635200	B	-0.72311000	-0.70921100	0.11324600
H	0.37874200	1.65159500	-2.29687000	O	-1.45172600	-0.76714900	1.25361600
C	1.51547400	4.13859200	-0.24821800	O	-1.38221100	-1.02617900	-1.02677200
H	1.18229900	4.24736200	1.88121400	C	-2.87246300	-0.90234700	0.82831600
H	1.69756600	3.77831200	-2.36822400	C	-2.72544200	-1.51610100	-0.61579400
H	2.09478600	5.05483600	-0.27895400	C	0.32895700	1.77358300	-0.13455600
C	-3.36867600	1.27264200	0.89825000	C	4.21904400	-0.80377000	-1.64228100
H	-4.38741000	1.58432200	0.65585900	H	5.01218000	-1.46113400	-1.27556200
H	-3.15502800	1.56695000	1.92842700	H	3.92700500	-1.14643100	-2.63821900
H	-2.67347800	1.80129000	0.24440800	H	4.61609100	0.20727000	-1.73470600
C	-4.03233700	0.11517200	-1.68098300	C	4.41792400	0.66050500	0.86038000
H	-3.98447000	-0.31258700	-2.68555700	H	4.43079400	1.07353900	1.87178000
H	-5.08249700	0.15877900	-1.37937000	H	5.39478000	0.21491900	0.65774100
H	-3.63805200	1.13046500	-1.72546700	H	4.25500500	1.48312800	0.16045200
C	-3.66105300	-2.21968300	-0.85103800	C	3.53504800	-1.50428200	1.76294300
H	-4.72434500	-2.34788900	-0.63499800	H	4.41214900	-2.09130600	1.47681700
H	-3.47533900	-2.54913700	-1.87598000	H	3.71680200	-1.08248400	2.75470700
H	-3.08659400	-2.86154200	-0.17936900	H	2.67426100	-2.16971900	1.83052500
C	-4.18066800	-0.95160600	1.71003100	C	2.34669300	-2.22801200	-0.79242700
H	-4.08428200	-0.53735200	2.71673000	H	1.99818500	-2.39359500	-1.81439100
H	-5.21160400	-0.80157900	1.37772100	H	3.04910000	-3.02310600	-0.53156200
H	-3.98266800	-2.02224900	1.76369200	H	1.48320900	-2.29250600	-0.12739400
C	3.70264200	-0.99469800	-1.61380400	C	-2.64865900	-3.04088100	-0.62881200
H	4.72502700	-1.20088100	-1.28522500	H	-3.61763400	-3.48550100	-0.38966000
H	3.56503300	-1.44509200	-2.60003400	H	-2.35454600	-3.37267200	-1.62702700
H	3.57703000	0.08355000	-1.71220300	H	-1.90860900	-3.40911300	0.08490300
C	3.30511800	0.41924700	0.89031700	C	-3.58859300	-1.78796000	1.83355800
H	3.14863000	0.80997400	1.89814300	H	-3.63042900	-1.28412200	2.80213300
H	4.37429900	0.45650100	0.66825000	H	-4.61414500	-1.97891100	1.50505800
H	2.77734400	1.06917500	0.19153600	H	-3.08055700	-2.74304900	1.96686100
C	3.53427300	-1.89464800	1.81954500	C	-3.45200200	0.51038600	0.83375900
H	4.57401000	-2.02053800	1.50503900	H	-4.51507800	0.49797800	0.58137400
H	3.53237600	-1.41959400	2.80382600	H	-3.33942900	0.93610300	1.83286700
H	3.07660200	-2.87921000	1.91752700	H	-2.92987700	1.16020800	0.12959100
C	2.73889500	-3.11821800	-0.69571800	C	-3.74019700	-1.01550100	-1.63033200
H	2.48840000	-3.43922500	-1.70953000	H	-3.55331000	-1.48535900	-2.59893400
H	3.73229700	-3.50028200	-0.44885900	H	-4.75349400	-1.27889300	-1.31409700
H	2.01257900	-3.56111800	-0.01054400	H	-3.68221400	0.06525000	-1.75807300

1e-TS'				2			
B	0.47265000	-0.57824000	0.31622300	C	-4.49186600	0.00001200	-0.00003100
O	0.65115800	-0.43902900	1.77416200	C	-3.79319800	-1.19772200	-0.16283700
O	1.20531200	-1.81128700	-0.04501700	C	-2.39942900	-1.19476400	-0.16164600
C	1.65225800	-1.36062900	2.21131100	C	-1.67844400	-0.00001000	0.00004000
C	1.53033500	-2.51696800	1.15422400	C	-2.39941900	1.19475600	0.16168800
B	-1.20289400	-0.61157400	-0.15210200	C	-3.79318800	1.19773500	0.16281300
O	-2.16413000	-1.35144500	0.52060400	H	-5.57682000	0.00002000	-0.00005800
O	-1.73480400	-0.10011200	-1.33397200	H	-4.33517000	-2.12893800	-0.28935400
C	-3.44661600	-1.21182700	-0.15166500	H	-1.86008800	-2.12785800	-0.28676400
C	-3.01899300	-0.73478700	-1.58800600	H	-1.86006900	2.12784000	0.28684200
N	1.23769900	0.67850000	-0.37466000	H	-4.33515200	2.12895900	0.28930800
C	2.17151900	0.79039700	-1.29531400	B	-0.12478900	-0.00001800	0.00008700
N	2.28232500	-0.09452800	-2.32272500	O	0.63168100	-1.12394800	-0.22051900
N	3.09092600	1.80706400	-1.24649200	O	0.63166500	1.12395100	0.22056600
C	3.57813800	-0.58863100	-2.77688800	C	2.02319900	-0.78268800	0.08502500
C	1.13265500	-0.83996300	-2.82314400	C	2.02318200	0.78269800	-0.08503200
C	3.56842500	2.47234900	-2.45899500	C	2.27809500	-1.23422600	1.52442500
C	3.27330700	2.58060800	-0.02298600	H	2.07119500	-2.30410100	1.60156300
H	3.71643000	-0.40642800	-3.84774700	H	3.31678600	-1.06220400	1.81557600
H	4.38892300	-0.11151100	-2.23005900	H	1.62592300	-0.70997800	2.22673200
H	3.63209800	-1.66871100	-2.59945600	C	2.93257200	-1.53338300	-0.87685400
H	1.10000300	-1.84660500	-2.40021000	H	3.97246200	-1.22730300	-0.73399700
H	0.20988500	-0.32818100	-2.56259000	H	2.86445800	-2.60688500	-0.68403200
H	1.21246600	-0.90045000	-3.91359400	H	2.65486900	-1.35616300	-1.91595700
H	4.66175900	2.46684700	-2.50013800	C	2.93264300	1.53342400	0.87677400
H	3.17907700	1.97887600	-3.34649400	H	3.97250000	1.22725700	0.73392500
H	3.22560700	3.51332300	-2.46567800	H	2.86464000	2.60693400	0.68394200
H	2.55474500	3.40788600	0.05121700	H	2.65494100	1.35624900	1.91589300
H	3.17124600	1.94114400	0.85238200	C	2.27802800	1.23418800	-1.52444900
H	4.28074300	3.00249200	-0.02595200	H	2.07064000	2.30395600	-1.60171000
H	1.06859400	1.52807500	0.14917100	H	3.31683600	1.06259900	-1.81543900
C	-1.32197600	2.04935000	0.90018500	H	1.62617400	0.70955600	-2.22678000
C	-0.90024800	2.30330400	2.18442700				
C	-1.78838400	2.98253100	0.00375600				
C	-0.97139200	3.63778300	2.61475300				
H	-0.51258700	1.51023700	2.81321900				
C	-1.84885400	4.31114500	0.45275000				
H	-2.09338900	2.70677900	-0.99936700				
C	-1.44325800	4.63036000	1.75137000				
H	-0.65389700	3.89636500	3.62029000				
H	-2.21170000	5.08884000	-0.21228300				
H	-1.49340500	5.65897300	2.09186300				
C	2.81691000	-3.30883800	0.92439900				
H	3.15884700	-3.78182900	1.85050200				
H	2.63774800	-4.09809000	0.18806500				
H	3.61412600	-2.66936700	0.54244200				
C	0.38095800	-3.48434900	1.48032300				
H	0.21928200	-4.13529700	0.61608400				
H	0.60548600	-4.11414700	2.34636200				
H	-0.54338400	-2.93487700	1.66293000				
C	3.03039400	-0.67826900	2.17443500				
H	3.31454800	-0.42397000	1.15326000				
H	2.97781800	0.24683500	2.75533900				
H	3.81193500	-1.30970700	2.60786900				
C	1.33810600	-1.77632900	3.64796700				
H	2.00537200	-2.57685200	3.98319000				
H	1.47250300	-0.92156600	4.31810800				
H	0.30604500	-2.11598800	3.74170600				
C	-2.74853000	-1.89208800	-2.55593600				
H	-3.67017300	-2.40683000	-2.83868200				
H	-2.28558600	-1.49409400	-3.46245700				
H	-2.06069800	-2.61813100	-2.11671200				
C	-3.95321400	0.28121300	-2.23369300				
H	-3.56134000	0.57001700	-3.21277500				
H	-4.94854900	-0.14830200	-2.37969800				
H	-4.04891300	1.18186100	-1.62715100				
C	-4.25222200	-0.16551700	0.62355400				
H	-5.25772300	-0.04745000	0.21124500				
H	-4.34184000	-0.49033300	1.66314100				
H	-3.75050400	0.80304700	0.61773900				
C	-4.16611100	-2.55567300	-0.11089600				
H	-4.41464700	-2.80591000	0.92380900				
H	-5.09748200	-2.51410600	-0.68326000				
H	-3.54442300	-3.35709600	-0.51090100				

**1f**				**1f'**			
B	-0.00009500	1.97708900	0.00013900	C	-2.17271200	-0.10559100	-0.50509900
O	-1.08308200	1.26275500	0.38394600	N	-0.94793300	-0.75416000	-0.82696500
O	1.08297900	1.26293900	-0.38376100	N	-3.08154500	-0.83757100	0.30201500
C	-0.78710300	-0.15965100	0.04316200	N	-2.17413300	1.27824200	-0.25684900
C	0.78710900	-0.15956700	-0.04329600	C	-2.06730700	1.78565800	1.11554300
C	-1.36910900	-1.03831400	1.13776500	H	-2.57110700	2.75562200	1.18630000
H	-2.45871600	-0.96027900	1.13015100	H	-2.55722300	1.09217100	1.79949400
H	-1.10184300	-2.08429400	0.96322000	H	-1.02167700	1.90430500	1.42842500
H	-1.01255300	-0.74623500	2.12529900	C	-1.53480600	2.12901000	-1.24589300
C	1.47534200	-0.42996900	1.29248200	H	-0.44615900	2.19915000	-1.10993600
H	1.35141000	-1.47333300	1.59183500	H	-1.73513700	1.75054400	-2.25021100
H	2.54335300	-0.22635900	1.18942400	H	-1.94762600	3.14113000	-1.17455400
H	1.08064200	0.21062000	2.08382100	H	-1.04418100	-1.54727400	-1.44682700
C	1.36948600	-1.03802300	-1.13788800	C	-4.48353200	-0.43916100	0.19208700
H	2.45910100	-0.96017400	-1.12962100	H	-5.03582100	-0.80524200	1.06140700
H	1.10196200	-2.08402000	-0.96381600	H	-4.56262500	0.64646100	0.16169600
H	1.01359100	-0.74558100	-2.12554100	H	-4.95492600	-0.84721800	-0.71781900
C	-1.47559600	-0.42990300	-1.29253000	C	-2.91962900	-2.28255200	0.37623500
H	-1.35182200	-1.47326200	-1.59196600	H	-3.16279000	-2.78777100	-0.57526900
H	-2.54357200	-0.22622600	-1.18924000	H	-1.89928000	-2.54284900	0.65582700
H	-1.08102500	0.21070500	-2.08391500	H	-3.59413900	-2.67156800	1.14231900
				B	0.37863700	-0.45139600	-0.43730600
				O	1.46320500	-1.11358100	-0.99192600
				O	0.74818100	0.48987600	0.50070000
				C	2.62684900	-0.76451700	-0.18495900
				C	2.19968600	0.62354800	0.42414800
				C	2.49589600	1.80273600	-0.50575600
				H	2.00108900	2.69478000	-0.11516300
				H	3.56842200	2.00253600	-0.56369500
				H	2.12028500	1.61828000	-1.51468500
				C	3.85275200	-0.71833700	-1.08612700
				H	4.72390400	-0.36325500	-0.52873900
				H	4.07364800	-1.72196300	-1.45838100
				H	3.69581000	-0.06506300	-1.94460400
				C	2.73579200	0.90414900	1.82086800
				H	3.82925000	0.91096900	1.81593500
				H	2.39082300	1.88542200	2.15665500
				H	2.39277600	0.16009900	2.54001000
				C	2.78480300	-1.85884700	0.87416100
				H	2.88334400	-2.82434200	0.37229900
				H	3.67500500	-1.69678000	1.48639200
				H	1.91258500	-1.90367500	1.53080300

complex2				1a-1			
B	0.28138400	0.49764000	-0.26624200	C	-2.79576600	0.06006700	0.13036200
O	0.46950400	1.56204500	-1.27687800	N	-1.63229500	0.55561700	0.45112000
O	0.87192100	1.02928300	0.97629100	N	-2.98023100	-1.30416100	0.11741600
C	1.34036900	2.56267100	-0.73705100	N	-3.89234200	0.82405100	-0.21925200
C	1.09111500	2.43123000	0.81026800	C	-5.23419900	0.49187300	0.26579600
B	-1.37615200	0.01078600	-0.21332300	H	-5.96465500	0.57314400	-0.54443700
O	-2.39128600	0.71328900	0.41861300	H	-5.26271200	-0.51902700	0.66524600
O	-1.88788600	-1.13442900	-0.81513400	H	-5.52542600	1.18373800	1.06634100
C	-3.59586100	-0.10497700	0.43413000	C	-3.70425800	2.24051100	-0.50595200
C	-3.34363800	-1.08558400	-0.76506300	H	-3.62072100	2.84579500	0.40808400
N	1.21037600	-0.75599600	-0.75271200	H	-2.81319000	2.39434700	-1.11590100
C	1.99027600	-1.59665200	-0.10720700	H	-4.56858000	2.59701400	-1.07020200
N	1.75315300	-1.94015500	1.18970300	H	-1.65246900	1.55665900	0.61346400
N	3.08961700	-2.15502100	-0.70510200	C	-3.74329600	-1.95911100	-0.94611400
C	2.83191500	-2.01542700	2.17174900	H	-4.42118900	-2.70758500	-0.52530400
C	0.41063400	-1.87834400	1.75745200	H	-4.32560000	-1.23537200	-1.51133800
C	3.50214700	-3.53128200	-0.42762800	H	-3.05911400	-2.46306500	-1.64060700
C	3.62637500	-1.60179400	-1.94325600	C	-1.96474000	-2.15737800	0.72365300
H	2.82760200	-2.98284300	2.68377100	H	-1.11068500	-2.32359500	0.05550900
H	3.79956700	-1.87268800	1.69513000	H	-1.59931200	-1.71322700	1.64840400
H	2.69643900	-1.22510600	2.91866700	H	-2.41982700	-3.12412500	0.95242200
H	0.20753300	-0.90158200	2.20541600	C	5.76400900	-0.50023500	-0.25224700
H	-0.32768700	-2.07768900	0.98361500	C	5.41650200	0.85013800	-0.18016900
H	0.32909300	-2.65432000	2.52385800	C	4.08160500	1.21914100	-0.03869300
H	4.54344200	-3.56633000	-0.09354200	C	3.08155200	0.24217600	0.03090100
H	2.86983200	-3.97397200	0.33857500	C	3.43520200	-1.11076700	-0.04262800
H	3.40974900	-4.13134900	-1.33985300	C	4.77168900	-1.47905800	-0.18297600
H	3.12350900	-2.01301300	-2.82876600	H	6.80400000	-0.78817500	-0.36178900
H	3.53226400	-0.51702400	-1.95129200	H	6.18552700	1.61288000	-0.23399600
H	4.68720100	-1.85444500	-2.00583400	H	3.79893600	2.26325200	0.01948000
H	1.30468400	-0.78242800	-1.76033900	H	2.66223400	-1.86661500	0.01119500
C	2.79317300	2.23739200	-1.12224800	H	5.03905500	-2.52861500	-0.23838600
H	3.12403200	1.31333100	-0.64841100	C	1.65238200	0.67619900	0.18447500
H	2.84760400	2.10710200	-2.20677200	O	0.79247400	-0.32442000	0.22560700
H	3.48129900	3.03984200	-0.83937300	O	1.33517200	1.85564800	0.26368400
C	0.95575900	3.91695900	-1.33103100	H	-0.19746800	0.02286700	0.32406400
H	1.51462400	4.72947700	-0.85569400				
H	1.18430900	3.93008800	-2.40119700				
H	-0.11131300	4.10941800	-1.21385900				
C	2.27349500	2.85407800	1.68125000				
H	2.53903600	3.90107000	1.50340000				
H	2.00952200	2.74747000	2.73770300				
H	3.15007900	2.23311200	1.49098200				
C	-0.17131000	3.18342800	1.26262200				
H	-0.39479100	2.89748400	2.29423200				
H	-0.03819700	4.26889100	1.22817600				
H	-1.02908200	2.90519200	0.64932400				
C	-3.63192400	-0.81089000	1.79367400				
H	-3.60838500	-0.05610500	2.58335800				
H	-4.53945100	-1.40797800	1.91363900				
H	-2.76414500	-1.46172300	1.92359300				
C	-4.81101200	0.80155900	0.28262800				
H	-5.72766600	0.20953900	0.20514000				
H	-4.89771900	1.44826200	1.15969400				
H	-4.72971900	1.43800500	-0.59884100				
C	-3.81573000	-0.52760500	-2.11102000				
H	-3.43473700	-1.16654600	-2.91157800				
H	-4.90646600	-0.50644800	-2.17752000				
H	-3.43391800	0.48272900	-2.27403500				
C	-3.87794400	-2.49857900	-0.56608300				
H	-4.96394100	-2.48594700	-0.43562200				
H	-3.64928700	-3.10570300	-1.44606100				
H	-3.42815700	-2.98030400	0.30265100				

1a-2				1a-3			
C	-1.36051500	-0.15257200	0.38876000	C	1.19983500	-0.41890300	-0.28537600
O	-0.91297200	-0.95025000	1.19632500	O	0.73611500	-0.99213700	-1.25381400
H	0.35999000	0.63489500	0.03321800	H	-0.50367500	-0.26759800	0.60373900
N	2.06783700	-1.62314900	0.26644600	N	-3.63829200	-0.86877200	-1.27202900
C	2.62365800	-0.48632600	0.04778400	C	-3.06045800	-0.22909600	-0.31780600
H	1.15825400	-1.50773400	0.71288500	H	-3.53183700	-1.87330200	-1.17189200
N	2.05470400	0.74511600	0.52159800	N	-2.21045200	-0.81899700	0.67482000
N	3.75057400	-0.37993100	-0.74552700	N	-3.24499400	1.13615700	-0.18176500
O	-0.59557800	0.72910900	-0.25838900	O	0.45583300	-0.02770600	0.75525500
C	-2.80561400	-0.06885400	0.03237000	C	2.64684100	-0.08369200	-0.13861100
C	-3.28481700	0.86897500	-0.89133400	C	3.14113900	0.58331800	0.98966300
C	-3.69727900	-0.95964400	0.64280100	C	3.52476500	-0.45185800	-1.16553700
C	-4.64248000	0.91259800	-1.19824600	C	4.49919400	0.87692300	1.08619300
H	-2.59662700	1.55728200	-1.36462600	H	2.46438000	0.86936600	1.78431400
C	-5.05289600	-0.91332000	0.33360200	C	4.88090700	-0.15732100	-1.06595300
H	-3.31624200	-1.68213600	1.35409400	H	3.13265700	-0.96652300	-2.03422600
C	-5.52719100	0.02289800	-0.58729800	C	5.37002100	0.50746100	0.06026700
H	-5.01026000	1.63928100	-1.91389700	H	4.87798500	1.39368800	1.96087000
H	-5.73924700	-1.60569500	0.80786900	H	5.55616100	-0.44464900	-1.86402800
H	-6.58398200	0.05836200	-0.82843000	H	6.42715300	0.73707700	0.13771800
C	2.38114000	2.02040000	-0.13544600	C	-1.89563500	-2.23993100	0.45646200
H	1.52303100	2.68973900	-0.02871200	H	-1.16465700	-2.54515200	1.20717700
H	3.24975500	2.52055300	0.30653200	H	-2.77873500	-2.88377100	0.56086300
H	2.56546200	1.85736100	-1.19594300	H	-1.44799500	-2.37784900	-0.52705900
C	1.91150300	0.88062800	1.98118600	C	-2.63407900	-0.59087300	2.07571800
H	1.14926300	1.63261400	2.19842400	H	-1.79957400	-0.82070600	2.74110900
H	1.59363300	-0.06273900	2.41988700	H	-2.93199600	0.44314400	2.22956900
H	2.85511000	1.19657900	2.44491200	H	-3.48026500	-1.23934000	2.33551000
C	4.92156900	0.34103000	-0.23709500	C	-2.13178100	2.01419500	0.18853300
H	4.65054700	1.02643700	0.56123900	H	-1.53509300	1.60243200	0.99675100
H	5.65008600	-0.37006700	0.17387100	H	-1.47100800	2.20481900	-0.66827800
H	5.40174200	0.90639900	-1.04045200	H	-2.53805700	2.96812600	0.52978800
C	4.10615600	-1.53713700	-1.56077600	C	-4.18459300	1.76175200	-1.10897600
H	4.54035700	-2.34885300	-0.96240700	H	-3.77206700	1.84188400	-2.12367500
H	3.22510300	-1.92122800	-2.07334100	H	-5.10484900	1.18228500	-1.16172400
H	4.84121000	-1.21823400	-2.30407900	H	-4.41488800	2.76425300	-0.74268700

Supplementary References

1. Candish, L.; Teders, M.; Glorius, F. Transition-Metal-Free, Visible-Light-Enabled Decarboxylative Borylation of Aryl N-Hydroxyphthalimide Esters. *J. Am. Chem. Soc.* **139**, 7440-7443 (2017).
2. Jin, S. F.; Dang, H. T.; Haug, G. C.; He, R.; Nguyen, V. D.; Nguyen, V. T.; Arman, H. D.; Schanze, K. S.; Larionov, O. V. Visible Light-Induced Borylation of C-O, C-N, and C-X Bonds. *J. Am. Chem. Soc.* **142,** 1603-1613 (2020).

3. Liu, C. W.; Ji, C. L.; Hong, X.; Szostak, M. Palladium-Catalyzed Decarbonylative Borylation of Carboxylic Acids: Tuning Reaction Selectivity by Computation. *Angew. Chem. Int. Ed.* **57**, 16721-16726 (2018).
4. Guerrand, H. D. S.; Marciasini, L. D.; Jousseaume, M.; Vaultier, M.; Pucheault, M. Borylation of Unactivated Aryl Chlorides under Mild Conditions by Using Diisopropylaminoborane as a Borylating Reagent. *Chem. Eur. J.* **20**, 5573-5579 (2014).
5. Clary, J. W.; Rettenmaier, T. J.; Snelling, R.; Bryks, W.; Banwell, J.; Wipke, W. T.; Singaram, B. Hydride as a Leaving Group in the Reaction of Pinacolborane with Halides under Ambient Grignard and Barbier Conditions. One-Pot Synthesis of Alkyl, Aryl, Heteroaryl, Vinyl, and Allyl Pinacolboronic Esters. *J. Org. Chem.* 76, 9602-9610 (2011).
6. Wollenburg, M.; Moock, D.; Glorius, F. Hydrogenation of Borylated Arenes. *Angew. Chem. Int. Ed.* **58**, 6549-6553 (2019).
7. Chow, W. K.; Yuen, O. Y.; So, C. M.; Wong, W. T.; Kwong, F. Y. Carbon-Boron Bond Cross-Coupling Reaction Catalyzed by -PPh2 Containing Palladium-Indolylphosphine Complexes. *J. Org. Chem.* **77,** 3543-3548 (2012).
8. Bacauanu, V.; Cardinal, S.; Yamauchi, M.; Kondo, M.; Fernandez, D. F.; Remy, R.; MacMillan, D. W. C. Metallaphotoredox Difluoromethylation of Aryl Bromides. *Angew. Chem. Int. Ed.* **57**, 12543-12548 (2018).
9. Labre, F.; Gimbert, Y.; Bannwarth, P.; Olivero, S.; Dunach, E.; Chavant, P. Y. Application of Cooperative Iron/Copper Catalysis to a Palladium-Free Borylation of Aryl Bromides with Pinacolborane. *Org. Lett.* **16**, 2366-2369 (2014).
10. Qiu, D.; Jin, L.; Zheng, Z. T.; Meng, H.; Mo, F. Y.; Wang, X.; Zhang, Y.; Wang, J. B. Synthesis of Pinacol Arylboronates from Aromatic Amines: A Metal-Free Transformation. *J. Org. Chem.* **78**, 1923-1933 (2013).
11. Larsen, M. A.; Wilson, C. V.; Hartwig, J. F. Iridium-Catalyzed Borylation of

Primary Benzylic C-H Bonds without a Directing Group: Scope, Mechanism, and Origins of Selectivity. *J. Am. Chem. Soc.* **137**, 8633-8643 (2015).

12. de Toledo, I.; Grigolo, T.; Bennett, J. M.; Elkins, J. M.; Pilli, R. A. Modular Synthesis of Di- and Trisubstituted Imidazoles from Ketones and Aldehydes: A Route to Kinase Inhibitors. *J. Org. Chem.* **84**, 14187-14201 (2019).
13. Prazeres, V. F. V.; Sanchez-Sixto, C.; Castedo, L.; Lamb, H.; Hawkins, A. R.; Riboldi-Tunnicliffe, A.; Coggins, J. R.; Lapthorn, A. J.; Gonzalez-Bello, C. Nanomolar competitive inhibitors of Mycobacterium tuberculosis and Streptomyces coelicolor type II dehydroquinase. *Chemmedchem* **2,** 194-207 (2007).
14. Choi, G. J.; Zhu, Q. L.; Miller, D. C.; Gu, C. J.; Knowles, R. R. Catalytic alkylation of remote C-H bonds enabled by proton-coupled electron transfer. *Nature* **539**, 268-271 (2016).
15. Kumar, K.; Gupta, B. D. Synthesis, characterization, CV, and X-ray structures of aryl cobaloximes. *J. Organomet. Chem.* **695**, 2233-2239 (2010).
16. Kuhn, H. J., Braslavsky, S. E. & Schmidt, R. Chemical actinometry (IUPAC Technical Report). *Pure Appl. Chem.* **76**, 2105–2146 (2004).
17. Montalti, M., Credi, A., Prodi, L. & Gandolfi, M. T. Chemical Actinometry. Handbook of Photochemistry, 3rd Ed. 601–616. (Taylor & Francis Group, Boca Raton, 2016).
18. Cismesia, M. A. & Yoon, T. P. Characterizing chain processes in visible light photoredox catalysis. *Chem. Sci.* **6,** 5426–5434 (2015).
19. Hatchard, C. G.; Parker, C. A. A New Sensitive Chemical Actinometer .2. Potassium Ferrioxalate as a Standard Chemical Actinometer. *Proc. Roy. Soc. (London),* **A235**, 518-536 (1956).
20. V. S. Bagotzky. Fundamentals of electrochemistry (2nd edition). Wiley, 2006. Russian Journal of Electrochemistry, 43(11), 1326–1327.
